# Multienzymatic biotransformation of flavokawain B by entomopathogenic filamentous fungi: structural modifications and pharmacological predictions

**DOI:** 10.1186/s12934-024-02338-9

**Published:** 2024-02-24

**Authors:** Paweł Chlipała, Tomasz Tronina, Monika Dymarska, Monika Urbaniak, Ewa Kozłowska, Łukasz Stępień, Edyta Kostrzewa-Susłow, Tomasz Janeczko

**Affiliations:** 1 Department of Food Chemistry and Biocatalysis, Wrocław University of Environmental and Life Sciences, Wrocław, Norwida 25, 50-375 Poland; 2 Institute of Plant Genetics, Polish Academy of Sciences, Poznań, Strzeszyńska 34, 60-479 Poland

## Abstract

**Background:**

Flavokawain B is one of the naturally occurring chalcones in the kava plant (*Piper methysticum*). It exhibits anticancer, anti-inflammatory and antimalarial properties. Due to its therapeutic potential, flavokawain B holds promise for the treatment of many diseases. However, due to its poor bioavailability and low aqueous solubility, its application remains limited. The attachment of a sugar unit impacts the stability and solubility of flavonoids and often determines their bioavailability and bioactivity. Biotransformation is an environmentally friendly way to improve the properties of compounds, for example, to increase their hydrophilicity and thus affect their bioavailability. Recent studies proved that entomopathogenic filamentous fungi from the genera *Isaria* and *Beauveria* can perform *O*-methylglycosylation of hydroxyflavonoids or *O*-demethylation and hydroxylation of selected chalcones and flavones.

**Results:**

In the present study, we examined the ability of entomopathogenic filamentous fungal strains of *Beauveria bassiana*, *Beauveria caledonica*, *Isaria farinosa*, *Isaria fumosorosea*, and *Isaria tenuipes* to transform flavokawain B into its glycosylated derivatives. The main process occurring during the reaction is *O*-demethylation and/or hydroxylation followed by 4-*O*-methylglycosylation. The substrate used was characterized by low susceptibility to transformations compared to our previously described transformations of flavones and chalcones in the cultures of the tested strains. However, in the culture of the *B. bassiana* KCh J1.5 and BBT, *Metarhizium robertsii* MU4, and *I. tenuipes* MU35, the expected methylglycosides were obtained with high yields. Cheminformatic analyses indicated altered physicochemical and pharmacokinetic properties in the derivatives compared to flavokawain B. Pharmacological predictions suggested potential anticarcinogenic activity, caspase 3 stimulation, and antileishmanial effects.

**Conclusions:**

In summary, the study provided valuable insights into the enzymatic transformations of flavokawain B by entomopathogenic filamentous fungi, elucidating the structural modifications and predicting potential pharmacological activities of the obtained derivatives. The findings contribute to the understanding of the biocatalytic capabilities of these microbial cultures and the potential therapeutic applications of the modified flavokawain B derivatives.

**Supplementary Information:**

The online version contains supplementary material available at 10.1186/s12934-024-02338-9.

## Background

Flavokawain B (1-(2ʹ-hydroxy-4ʹ,6ʹ-dimethoxyphenyl)-3-phenyl-prop-2-en-1-on) (FB1) is one of the naturally occurring chalcones in the kava plant (*Piper methysticum*) [1]. Kava is a perennial shrub from the Piperaceae (pepper) family, which is native to the ethnogeographic regions of Melanesia, Micronesia and Polynesia [2]. Since the 1990s, kava has been popularized amongst Western countries due to its sedative, anti-stress and anxiolytic properties [3, 4]. Across European and American markets, kava is sold in tablet or capsule form to treat anxiety disorders [5, 6]. The primary active constituents responsible for the pharmacological effects of kava are known as kavalactones and kavapyrones [7]. Kava chalcone- flavokawain B (FB1) exhibits anti-malarial [8], anti-inflammatory [9] and anti-angiogenic [10] properties; it also weakens the progression of gastric cancer [11]. Compound FB1 substantially slows down the development of colon cancer, inducing mitochondria-dependent apoptosis, which is characterized by the release of cytochrome c [12]. It also plays a crucial role in melanoma cells’ ability to execute and produce ROS-modulated apoptotic and autophagic cell death. Flavokawain B (FB1) shows the potential to inhibit tumor growth in nude mice with xenografts [13] and may represent a novel therapeutic option for patients with synovial sarcoma [14]. This chalcone exhibits toxicity towards various breast cancer cell lines [10, 15] and has been shown to inhibit the growth of human osteosarcoma cells through G2/M cell cycle arrest [16]. However, flavokawain B (FB1), like other chalcones, has poor water solubility and bioavailability, which reduces its in vivo biological effects [17–19]. Microbial glycosylation and methylation is a known method of improving the stability, water solubility, and bioavailability of chalcones [20]. In vivo studies have shown that glycosylation may significantly improve the bioavailability of quercetin (flavonol); however, the impact of glycosylation strongly depends on the type of sugar attached [21]. For the two quercetin glycosides isoquercetin (glucoside) and rutin (rutoside), the relative total bioavailability was 148% and 23%, respectively, compared with quercetin aglycone [22]. Certain glycosides, such as quercetin-3-*O*-glucoside (isoquercetin), are substrates for the small intestinal brush border enzyme—lactase-phlorizin hydrolase (EC 3.2.1.108) [23]. The glucoside form of quercetin can be enzymatically converted to aglycone quercetin, and thus be absorbed mainly in the small intestine. This results in relatively high bioavailability of quercetin after oral administration of isoquercetin [24].

Utilization of whole-cell biocatalysts provides a convenient approach to execute enzymatic cascades encompassing multiple reactions, while simultaneously providing the necessary cofactors required for these intricate biotransformations [25]. Cell coverings protect and stabilize enzymes, enabling them to be used in challenging reaction conditions [26]. Using whole cells in biocatalysis obviates the requirement for cell lysis and the subsequent purification of enzymes, resulting in a significant reduction in catalyst costs. The presence of the intact cell enables the internal supply and regeneration of expensive cofactors, eliminating the need for external supplementation and further reducing costs. Furthermore, whole-cell biocatalysts are generally simpler to prepare, costs of maintaining the cultures are typically feasible, and cells can often be employed repeatedly, enhancing cost-effectiveness [26, 27].

The main aim of this study was to investigate the ability of entomopathogenic filamentous fungi of the genera *Beauveria*, *Isaria* and *Metarhizium* to perform biotransformation of flavokawain B (FB1) and characterize products of this process. Studies have indicated that entomopathogenic fungal strains such as *B. bassiana*, *B. caledonica*, *Isaria farinosa*, and *I. fumosorosea* may be effectively used as biocatalysts in biotransformations of chalcones [20], flavones [28–30], flavanones [29–31] and steroids [32–35]. *B. bassiana*, an ascomycete fungus, has garnered attention as a versatile pathogenic agent, effectively targeting a wide spectrum of insect species. This remarkable fungus has been harnessed on a commercial scale as an environmentally friendly insecticide. The hallmark of its biological potential lies in the synthesis of a rich array of versatile enzymes and secondary metabolites, including non-peptides and polyketides. Notably, some of these metabolites, such as oxalic acid, demonstrate potent pathogenic and virulent properties, rendering them appealing for applications across diverse sectors, ranging from industrial to pharmaceutical and agricultural realms [36]. *B. bassiana* exhibits a noteworthy capability for glycosylation of flavonoid compounds such as quercetin [37], warfarin [38], cannflavin B [39] and xanthohumol [40] as well as hydroxylation of steroids such as 1-dehydrotestosterone, testosterone, 19-nortestosterone, 17α-methyltestosterone and progesterone [32]. In addition, *I. farinosa* and *I. fumosorosea* strains show the ability to biotransform 2’-hydroxy-2-methylchalcone [41], 2’-methylflavone [30], methoxyflavones [42, 43] and steroids [44, 45]. *Metarhizium* strains can also be used as biocatalysts in the bioconversion of quercetin [46] and steroids [35, 45].

## Results and discussion

In light of the recent findings on the enzymatic modification of flavonoids within the milieu of the entomopathogenic microbial cultures, we decided to employ three distinct strains of *Beauveria bassiana* (namely KCh J1.5, J1 and BBT) and three strains from the genus *Isaria* (specifically, *I. tenuipes* MU35, *I. fumosorosea* KCh J2 and *I. farinosa* KCh KW 1.1) as well as a strain of *Beauveria caledonica* (designated as KCh J3.3) and *Metarhizium robertsii* (designated as MU4), formerly known as *Metarhizium anisopliae*, to catalyze biotransformations of (1-(2ʹ-hydroxy-4ʹ,6ʹ-dimethoxyphenyl)-3-phenyl-prop-2-en-1-on) – flavokawain B (FB1) [29–31, 47–51]. In this study we decided to determine whether entomopathogenic filamentous fungi transform flavokawain B (FB1) as in case of flavones. Flavokawain B (FB1) was obtained through chemical synthesis, purified by crystallization from ethanol, and its structure was confirmed spectroscopically (Additional file 1: Figures S1–S7) according to the procedure described in earlier publications [42, 52].

### Biotransformation of FB1 in the cultures of entomopathogenic filamentous fungi

Biotransformation of the substrate (FB1) in the cultures of selected entomopathogenic, filamentous fungal strains led to obtaining seven identified derivatives.

The products were extracted from the cultures using ethyl acetate then purified with preparative thin-layer chromatography (pTLC). The structures of the purified products were determined using nuclear magnetic resonance spectroscopy (NMR), and retention times (Rt) were determined with ultra-high performance liquid-chromatography (UHPLC) data obtained during the screening procedure. All selected strains except *I. fumosorosea* KCh J2 and *I. farinosa* KCh KW1.1 (> 99.9% of the unconverted substrate in the reaction mixture after 10 days of incubation) showed the ability to perform biotransformation of the substrate (FB1). The best conversion rate was observed in *B. bassiana* KCh BBT and *B. bassiana* KCh J1.5 (0.5% and 1.6%, respectively, of the remaining substrate in the reaction mixture after 10 days and less than 5% of unreacted substrate after 3 days of biotransformation) (Table 1). Based on TLC and HPLC analyses, it was observed that the resulting products were characterized by significantly higher polarity than the substrate (FB1). This indicated that the main products, akin to the previously described biotransformations of flavonoid compounds [41, 42], are most likely the result of 4-*O*-methylglycosylation.

**Table 1:** Composition of the reaction mixture [%] after 1, 3, 7 and 10 days obtained during biotransformation of flavokawain B (FB1) by HPLC

Strain	Compound	Retention time by HPLC [min]	Time of biotransformation [days]			
			1	3	7	10
*Metarhizium robertsii* MU4	FB1	9.578	33.6	10.4	4.6	4.3
	FB2	9.075	0.8	0.6	0.6	1.2
	FB3	5.795	44.4	68.2	75.0	75.8
	FB4	4.641	2.0	2.3	2.5	2.5
	FB5	4.566	7.7	15.8	15.8	16.1
	u.c	-	11.5	2.7	1.5	0.1
*Isaria tenuipes* MU35	FB1	9.578	89.0	75.5	63.4	53.5
	FB2	9.075	4.1	6.2	12.1	9.8
	FB3	5.795	6.9	18.3	24.5	36.7
*Isaria fumosorosea* KCh J2	FB1	9.578	> 99.9	> 99.9	> 99.9	> 99.9
*Isaria farinosa* KCh KW1.1	FB1	9.578	> 99.9	> 99.9	> 99.9	> 99.9
*Beauveria bassiana* KCh BBT	FB1	9.578	76.7	2.3	0.9	0.5
	FB3	5.795	2.0	3.3	4.7	3.8
	FB4	4.641	2.2	16.0	16.0	20.0
	FB6	8.905	1.8	1.7	0.6	0.5
	FB7	5.729	10.5	43.0	43.5	32.5
	FB8	5.629	3.6	20.3	22.4	27.5
	u.c	-	3.2	13.4	11.9	15.2
*Beauveria bassiana* KCh J1.5	FB1	9.578	13.6	4.6	1.5	1.6
	FB3	5.795	2.4	0.0	0.0	0.0
	FB4	4.641	13.6	16.7	16.3	19.9
	FB6	8.905	0.8	0.1	0.05	0.0
	FB7	5.729	31.2	40.4	44.1	41.6
	FB8	5.629	27.7	30.2	30.3	31.2
	u.c	–	10.7	8.0	7.8	7.7
*Beauveria bassiana* KCh J1	FB1	9.578	99.0	95.0	90.4	87.1
	FB2	9.075	0.1	0.3	0.8	1.1
	FB6	8.905	0.9	4.7	9.8	11.8
*Beauveria caledonica* KCh J3.3	FB1	9.578	99.2	95.4	82.3	54.5
	FB3	5.795	0.7	3.5	10.6	21.2
	FB4	4.641	0.0	0.4	2.7	9.8
	FB7	5.729	0.1	0.7	4.4	14.6

u.c. unidentified compounds. Data are expressed as the mean of three independent experiments (biological replicates). Standard deviations were in the range 0–5

**Fig. 1:**
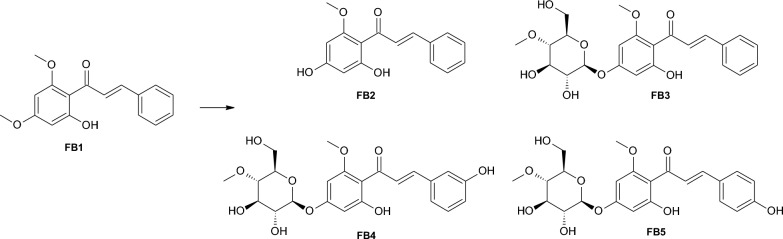
Biotransformation of flavokawain B (FB1) by *M*. *robertsii* MU4 strain

In the case of *M. robertsii* MU4 several products were obtained (four identified) during the process of biotransformation (Figure 1). The product of demethylation and 4-*O*-methylglycosydation (FB3) appears to be the main product of the biotransformation (75.8% content in the reaction mixture after 10 days of the biotransformation) with a retention time of about 5.795 min according to UHPLC.

The product was identified as 1-(4ʹ-*O*-β-D-(4ʹʹʹ-*O*-methylglucopiranosyl)-2ʹ-hydroxy-6ʹ-methoxyphenyl)-3-phenyl-prop-2-en-1-on – 4ʹ-*O*-β-D-(4ʹʹ-*O*-methylglucopyranosyl)-cardamonin (FB3) using NMR spectroscopy (Additional file 1: Figures S15–S21). The structure of the chalcone core (FB1) is confirmed by the ^1^H NMR, ^13^C NMR, COSY, HSQC and HMBC correlation spectra. However, instead of the signal from the protons of one of the -CH_3_ groups (visible in the ^1^H NMR substrate spectrum), signals from the sugar unit are visible. The multiplicities and positions of these signals in both the ^1^H NMR and ^13^C NMR spectra indicate that a 4-*O*-methylglucopyranosyl molecule was introduced in place of this -CH_3_ group. The HMBC spectrum indicated the coupling of the signal from the C-4ʹ carbon from the chalcone skeleton and H-1ʹʹʹ from the sugar unit, which exactly indicates the attachment position of the sugar unit to the chalcone core. The strains of entomopathogenic filamentous fungi have a unique capacity for 4-*O*-methylglycosylation of flavonoids. This reaction is most often described for flavonoid compounds containing a free hydroxyl group in their structure [48, 53–58]. The ability of entomopathogenic strains to hydroxylate and 4-*O*-methylglycosylate flavonoid compounds has also been described [31, 42, 59]. In our previous research, we also observed that the fungi perform demethylation and then 4-*O*-methylglycosylation of methoxyflavones [42, 47]. However, in one of our previous studies, we observed that a series of methoxy derivatives of chrysin, apigenin, and tricetin did not undergo glycosylation. Instead, we observed the tested strains’ ability to selectively demethylate/hydroxylate carbon C-3′ and C-4′ of ring B of the substrates used [43]. Flavokawain B (FB1) also contains two methoxy groups in positions analogous to those in 5,7-dimethoxyflavone. Nevertheless, obtaining 4ʹ-*O*-β-D-(4″-*O*-methylglucopyranosyl)-cardamonin (FB3) in the culture of *M. robertsii* MU4 strain indicates that the test compound (FB1) undergoes progressive demethylation and 4-*O*-methylglucosylation.

The product of 4ʹ-*O*-demethylation of the flavokawain B (FB1) known as cardamonin (FB2).

(Rt around 9.075 min) was also observed in the reaction mixture (1.2% of reaction mixture composition after 10 days). Due to the small amount of compound FB2 obtained, its structure was established based only on a comparison of the ^1^H NMR spectrum obtained for this compound with analogous data previously published in the literature (Additional file 1: Figures S10–S12) [60].

Compound FB4 was characterized by Rt around 4.641 min and it was identified by NMR spectra analysis as 1-(4ʹ-*O*-β-D-(4ʹʹʹ-*O*-methylglucopiranosyl)-2ʹ-hydroxy-6ʹ-methoxyphenyl)-3-(3ʹʹ-hydoxyphenyl)-prop-2-en-1-on – 4ʹ-*O*-β-D-(4″-*O*-methylglucopyranosyl)-3ʹʹ-hydroxycardamonin (FB4) (Additional file 1: Figures S24–S30). In the ^1^H NMR spectrum obtained for this compound (similarly to compound FB3), characteristic signals originating from the 4-*O*-methylglucopyranosyl substituent were observed, along with the observation of 4ʹ-*O*-demethylation. The position of the sugar unit was identified in a similar way as in the case of product FB3. Based on the ^13^C NMR spectrum analysis, it was determined that compound FB4 also underwent hydroxylation in ring B. In the HMBC spectrum, couplings were observed between the signal from the introduced –OH group proton and the signals from the C-2ʹʹ and C-4ʹʹ carbons, confirming unequivocally the positioning of the introduced hydroxyl group.

The second most abundant product in the reaction mixture was the compound identified as 1-(4ʹ-*O*-β-D-(4ʹʹʹ-*O*-methylglucopiranosyl)-2ʹ-hydroxy-6ʹ-methoxyphenyl)-3-(4ʹʹ-hydoxyphenyl)-prop-2-en-1-on – 4ʹ-*O*-β-D-(4″-*O*-methylglucopyranosyl)-4ʹʹ-hydroxycardamonin (FB5), which constituted 16.1% of the reaction mixture (after 10 days, Rt around 4.566 min). The NMR spectra which allowed us to identify product (FB5) (Additional file 1: Figures S33–S39) indicated no changes in the core of the chalcone backbone. Similarly to products FB3 and FB4, the structure of ring A for compound FB5 was determined. In the ^1^H NMR spectrum obtained for compound FB5, a characteristic signal pattern typical for a para-substituted aromatic ring was observed, unequivocally indicating the introduction of a hydroxyl group at carbon C-4ʹʹ. As a result of enzyme activity produced by the *M. robertsii* MU4 strain, products generated through cascading transformations were identified: 4ʹ-*O*-demethylation (compounds FB2-FB5), 4ʹʹ-*O*-methylglucopyranosylation (compounds FB3-FB5), and hydroxylation of ring B (compounds FB4 and FB5). In the culture of this strain, additional products were also formed, albeit in small quantities that impeded determination of their structure. The reaction mixture contained a decreasing amount of unidentified products between the first and the last day of biotransformation (from 11.5% to 0.1%); it may indicate that these compounds were intermediates in the formation of identified products (FB3–FB5).

**Fig. 2:**
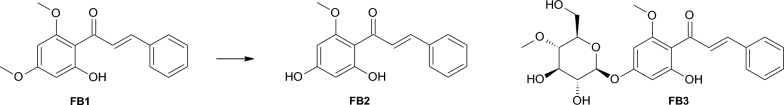
Biotransformation of flavokawain B (FB1) by *I*. *tenuipes* MU35 strain

**Fig. 3:**
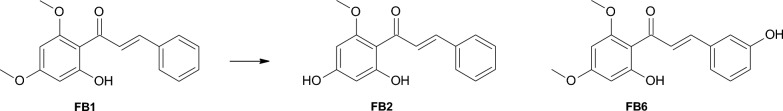
Biotransformation of flavokawain B (FB1) by *B*. *bassiana* KCh J1 strain

Two products were obtained during flavokawain B (FB1) biotransformation in the culture of *Isaria tenuipes* MU35 (Fig. 2) and *Beauveria bassiana* KCh J1 (Fig. 3). In both cultures, the formation of cardamonin (FB2) is noticeable at 9.8% and 1.1%, respectively, in the reaction mixture after 10 days of biotransformation). In the culture of *I. tenuipes* MU35, the main product is 4ʹ-*O*-β-D-(4″-*O*-methylglucopyranosyl)-cardamonin (FB3) (36.7% content of the reaction mixture after 10 days of biotransformation).

The product identified as 1-(2ʹ-hydroxy-4ʹ,6ʹ-dimethoxyphenyl)-3-(3ʹʹ-hydroxyphenyl)-prop-2-en-1-on – 3ʹʹ-hydroxyflavokawain B (FB6) (Additional file 1: Figures S42–S44) appears in the culture of *B. bassiana* KCh J1 as the main product (11.8% after 10 days, Rt about 8.905 min). However, this substance is also noticeable in the cultures of *B. bassiana* BBT and KCh J1.5 in decreasing amounts during the biotransformation process (1.8–0.5% and 0.8–0.0%, respectively), which may indicate that this product is an intermediate in the process of formation of the other products with a 4-*O*-methylglucopyranosyl group. In previous publications, we reported clear differences in the metabolism of the applied substrates in cultures of various strains belonging to the species *B. bassiana* [34, 42, 46]. We also found that the strain *B. bassiana* KCh J1 does not possess the ability to glycosylate flavonoid compounds [42, 46], which we also confirmed in the present study.

**Fig. 4:**
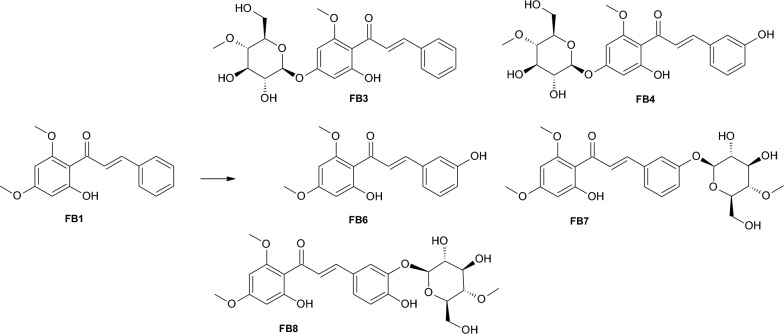
Biotransformation of flavokawain B (FB1) by *B*. *bassiana* BBT and KCh J1.5 strains

Biotransformation in the cultures of *B. bassiana* BBT and KCh J1.5 led to obtaining six identified products (Fig. 4). Products FB3, FB4, FB6 were also identified in the cultures of the strains *M. robertsii* MU4 and *B. bassiana* KCh J1, but two main products were obtained: 1-(2ʹ-hydroxy-4ʹ,6ʹ-dimethoxyphenyl)-3-(3ʹʹ-*O*-β-D-(4ʹʹʹ-*O*-methylglucopiranosyl)-phenyl)-prop-2-en-1-on – 3ʹ-*O*-β-D-(4″-*O*-methylglucopyranosyl)-flavokawain B (FB7) and 1-(2ʹ-hydroxy-4ʹ,6ʹ-dimethoxyphenyl)-3-(3ʹʹ-*O*-β-D-(4ʹʹʹ-*O*-methylglucopiranosyl)-4ʹʹ-hydroxyphenyl)-prop-2-en-1-on – 3ʹ-*O*-β-D-(4″-*O*-methylglucopyranosyl)-4ʹʹ-hydroxyflavokawain B (FB8) accounted for 32.5% and 41.6%, respectively, after 10 days of the reaction in *B. bassiana* KCh J1.5 culture.

The product (FB7) with Rt about 5.729 min was identified by NMR spectra analysis (Additional file 1: Figures S47–S53). In the ^1^H NMR spectrum obtained for this compound, it was observed that the modification occurred within the B ring of the chalcone skeleton. Based on chemical shifts, multiplicity of observed signals in the ^1^H NMR spectrum, and signal positions in the ^13^C NMR spectrum, it was determined that the functionalization occurred at carbon C-3ʹ. It was also confirmed that the structure of compound FB7 contains a 4-*O*-methylglucopyranosyl substituent. In the HMBC spectrum, coupling was observed between the signal from proton H-1ʹʹʹ (sugar unit) and the signal from carbon C-3ʹ, unequivocally indicating that the isolated main product formed in the cultures of *B. bassiana* BBT and KCh J1.5 strains is 3ʹ-*O*-β-D-(4″-*O*-methylglucopyranosyl)-flavokawain B (FB7). Product FB8, which was identified as 3ʹ-*O*-β-D-(4″-*O*-methylglucopyranosyl)-4ʹʹ-hydroxyflavokawain B, was assigned to Rt around 5.629 min. The product is formed in the cultures of *B. bassiana* BBT and KCh J1.5 (27.5% and 31.2%, respectively, of the reaction mixture after 10 days). In the ^1^H NMR spectrum obtained for compound FB8, analogous to the spectra obtained for product FB7, signals originating from protons and carbons in the A ring and propanoic chain were observed at appropriate positions and multiplicities in the ^1^H NMR spectrum, as well as in the ^13^C NMR spectrum. The presence of the 4-*O*-methylglucopyranosyl group in the structure of this compound was also noted. Based on the multiplicity of signals from the B ring protons and the chemical shifts of signals from carbons C-3ʹ and C-4ʹ observed at 147.57 and 146.88, respectively, in the ^13^C NMR spectrum, it was unequivocally determined that this compound resulted from dihydroxylation. In the HMBC spectrum, coupling between the signal from proton H-1ʹʹʹ (sugar unit) and the signal from carbon C-3ʹ was observed, allowing for the determination of its structure as 3ʹ-*O*-β-D-(4″-*O*-methylglucopyranosyl)-4ʹʹ-hydroxyflavokawain B (FB8) (Additional file 1: Figures S56–S62).

**Fig. 5:**
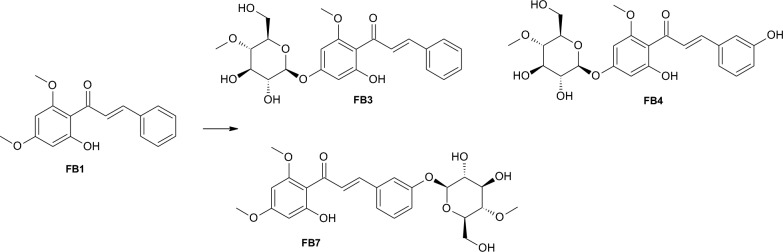
Biotransformation of flavokawain B (FB1) by *B*. *caledonica* KCh J3.3

In the culture of *B. caledonica* KCh J3.3 biotransformation of the FKB, three products were obtained (Fig. 5). The main product, which constituted 21.2% of the reaction mixture after 10 days, appeared to be 4ʹ-*O*-β-D-(4″-*O*-methylglucopyranosyl)-cardamonin (FB3).

Formation of 4ʹ-*O*-β-D-(4″-*O*-methylglucopyranosyl)-3ʹʹ-hydroxycardamonin (FB4) and product FB7 identified as 3ʹ-*O*-β-D-(4″-*O*-methylglucopyranosyl)-flavokawain B (FB7) was also observed (9.8% and 14.6% respectively after 10 days).

Based on the conducted experiments, it was observed that the strain *M. robertsii* MU4 predominantly produces products resulting from modifications of the A ring in the flavokawain B structure; 4-*O*-methylglucopyranosylation was preceded by 4ʹ-*O*-demethylation. However, in cultures of *B. bassiana* BBT and KCh J1.5 strains, transformations within the B ring were observed; the attachment of the sugar unit was preceded by hydroxylation. In our previous publications, through the biotransformation of 2ʹ-hydroxy-2-methylchalcone and 2ʹ-hydroxy-4-methylchalcone in *B. bassiana* KCH J1.5 culture, we obtained dihydrochalcones and chalcones with a 4-*O*-methylglucopyranosyl group located in the A ring [20, 41]. In the culture of the strain *I. fumosorosea* KCh J2, the respective 4″-*O*-methylglucopyranosyl chalcones were obtained with high efficiency [20, 41]. In this study, we found that the type and positioning of the substituents present in flavokawain B (FB1) hinder its transformation in the culture of the strain *I. fumosorosea* KCh J2. Simultaneously, none of the obtained biotransformation products of flavokawain B (FB1) resulted from the action of enzymes reducing the double bond of the substrate used. Additionally, in cultures of unconventional yeasts efficiently conducting the hydrogenation reaction, flavokawain B (FB1) remained unhydrogenated [61]. The in vitro metabolism of flavokawain B was examined using human liver microsomes. Major phase I metabolites were generated by demethylation in position C-4ʹʹ, yielding cardamonin, and hydroxylation predominantly in position C-4, yielding flavokawain C as a phase I metabolite [1]. The course of transformations in phase I observed is similar to what we observed in the culture of *M. robertsii* MU4 (described in this publication). This similarity is also evident in the experiment where flavokawain B was metabolized in the presence of uridine diphosphate (UDP) glucuronic acid by microsomal UDP-glucuronosyl transferases [1]. Flavokawain B in the colorectal cancer cell line LoVo and its doxorubicin-resistant subline LoVo/Dx was converted to the corresponding flavanone 5,7-dimetoxyflavanone. In a previous study it was found that cyclization of chalcone was related to a significant decrease in cytotoxicity [62]. In the cultures of entomopathogenic filamentous fungi described in this publication, we did not observe transformation of flavokawain B (FB1) into the respective flavanone.

Cheminformatics tools such as SwissADME, PreADMET and passOnline were used to compare the physicochemical characteristics, pharmacokinetics, and possible biological activities of flavokawain B (FB1) and its derivatives obtained via biotransformations. Calculations of the physicochemical descriptors for the substrate and the seven products (FB2–FB8) were performed (Additional file 1: Figures S8, S9, S13, S14, S22, S23, S31, S32, S40, S41, S45, S46, S54, S55, S63, S64). Absorption, distribution, metabolism, and excretion (ADME) factors, pharmacokinetic characteristics, and use for medicinal chemistry were all predicted. The Swiss Institute of Bioinformatics (SIB)’s Molecular Modeling Group developed and maintains the online resource SwissADME, which was used to conduct the analysis (http://www.swissadme.ch accessed on September 29, 2023) [63].

Based on the outcomes of this instrument, it was discovered that all of the products indicate lower lipophilicity than flavokawain B (FB1) (Table 2). Moreover, whether there is a hydroxyl group attached to the B ring of the chalcone core or a methylglycoside; the substance is not absorbed from the gastrointestinal tract (FB4, FB5, FB7, FB8). Substances FB1, FB2, FB6, which do not have a sugar unit attached to the chalcone core, exhibit BBB permeability and they cannot be transported by the P-glycoprotein. The simulations are in line with current research on chalcones [64–66] and their glycosides [67]. None of the obtained methylglycosides show the ability to inhibit any of the simulated CYP isoforms. This characteristic allows them to be classified as safe in terms of drug-drug interactions [68, 69]. On the other hand, the usage of aglycons and their potential to inhibit the CYP family may have a beneficial effect in terms of lowering treatment costs due to the medications’ reduced dosages with intact drug activity [70, 71]. The Caco-2 permeability assay is considered as the gold standard method to evaluate both the passive and active transport and the absorption of orally administered drugs [72]. Prediction of permeability of substances FB1–FB8 was performed using the online tool PreADMET (https://preadmet.webservice.bmdrc.org/adme/ accessed September 29, 2023). Collected data are presented in Table 2. Studies have indicated that flavonoids with an attached sugar unit are characterized by lower permeability in the Caco-2 model than the unsubstituted ones [73, 74].

**Table 2:** Data collected by the web tool SwissADME about pharmacokinetics and pharmacodynamics

Activity	Compound							
	FB1	FB2	FB3	FB4	FB5	FB6	FB7	FB8
Lipophilicity (Consensus Log P_o/w_)	3.06	2.6	1.34	0.84	0.83	2.68	1.44	0.93
GI absorption	High	High	High	Low	Low	High	Low	Low
BBB permeant	Yes	Yes	No	No	No	Yes	No	No
Pgp substrate	No	No	Yes	Yes	Yes	No	Yes	Yes
CYP1A2 inhibitor	Yes	Yes	No	No	No	Yes	No	No
CYP2C19 inhibitor	Yes	No	No	No	No	No	No	No
CYP2C9 inhibitor	Yes	Yes	No	No	No	Yes	No	No
CYP2D6 inhibitor	No	No	No	No	No	No	No	No
CYP3A4 inhibitor	Yes	Yes	No	No	No	Yes	No	No
log Kp (cm/s)	− 5.31	− 5.46	− 7.83	− 8.18	− 8.18	− 5.67	− 8.03	− 8.38
TPSA [Å^2^]	55.76	66.76	134.91	155.14	155.14	75.99	144.14	164.37
Bioavailability Score	0.55	0.55	0.55	0.55	0.55	0.55	0.55	0.55
Caco_2_	3.3024	1.5977	1.2027	0.9497	1.254	1.3496	0.5654	1.1716
HIA	95.582	92.833	83.525	68.065	68.065	93.199	81.663	63.994

PSA—(topological polar surface area), the surface sum over all polar atoms of the molecule (oxygen, nitrogen, sulfur and phosphorus), including their attached hydrogen atoms. Caco2—in vitro Caco-2 cell permeability (× 10^–6^ cm/sec); HIA human intestinal absorption (HIA, %)

Potential bioactivities were calculated using the PassOnline tool (http://www.way2drug.com/passonline/predict.php accessed on September 30, 2023). Table 3 shows predictions of the possible activities of flavokawain B (FB1) and the obtained metabolites (FB2–FB8). In most cases, the activities of metabolites FB2–FB8 are higher than the activities of flavokawain B (FB1) or comparable. Chalcones are generally described as anticarcinogenic [75–77] as are their glycosides [78, 79]. A bioinformatics tool predicted that the anticarcinogenic activity of the methylglycosyl residues of FB1 is much more possible than non-sugar metabolites (possibility of 47.6–53.0% to 88.1–90.1%, respectively). According to the predictions, all of substances FB1–FB8 will stimulate caspase 3, leading to cell apoptosis, with a high probability. Bioinformatics projections align with the current state of knowledge [80–84]. It is expected that products FB2–FB5, FB7, and FB8 will indicate antileishmanial activity in accordance with the in silico studies. A great number of flavokawain B (FB1) analogues showed antiprotozoal activity [85], as did other chalcones [86, 87]. Also, some of the aurones synthesized from flavokawain B (FB1) showed toxicity towards *Leishmania infantum* at a similar level as amphotericin B [88].

**Table 3:** Biological activity predictions for flavokawain B (FB1) and its metabolites (FB2–FB8) using the passOnline tool

Compound	Activity	Membrane integrity agonist	Caspase 3 stimulant	Antiprotozoal (*Leishmania*)	Free radical scavenger	Hepatoprotectant	Anticarcinogenic	Vasoprotective
FB1	Pa	0.894	0.892	0.765	0.599	0.569	0.476	0.608
	Pi	0.012	0.004	0.006	0.006	0.015	0.022	0.019
FB2	Pa	0.906	0.865	0.759	0.626	0.599	0.507	0.587
	Pi	0.010	0.004	0.006	0.005	0.012	0.019	0.022
FB3	Pa	0.931	0.952	0.947	0.873	0.907	0.881	0.896
	Pi	0.005	0.003	0.002	0.002	0.002	0.003	0.003
FB4	Pa	0.941	0.955	0.948	0.921	0.913	0.897	0.867
	Pi	0.004	0.003	0.002	0.002	0.002	0.003	0.004
FB5	Pa	0.940	0.947	0.948	0.914	0.915	0.895	0.876
	Pi	0.004	0.003	0.002	0.002	0.002	0.003	0.003
FB6	Pa	0.911	0.895	0.768	0.682	0.586	0.530	0.527
	Pi	0.008	0.004	0.006	0.004	0.013	0.017	0.033
FB7	Pa	0.935	0.969	0.950	0.909	0.903	0.888	0.875
	Pi	0.005	0.002	0.002	0.002	0.002	0.003	0.003
FB8	Pa	0.927	0.966	0.957	0.934	0.920	0.904	0.856
	Pi	0.005	0.002	0.002	0.001	0.002	0.002	0.004

Pi stands for likely idleness, and Pa for likely activity. The values range from 0 to 1, with 0 denoting no likelihood of Pa or Pi and 1 denoting 100% probability

## Materials and methods

### Substrates

The substrates 2-hydroxy-4,6-dimetoxyacetophenone and benzaldehyde were obtained from Sigma-Aldrich (St. Louis, MO, USA). FB1 was synthesized from these substrates. Its NMR spectral data are presented in Additional file Materials.

### Synthesis

**Fig. 6:**
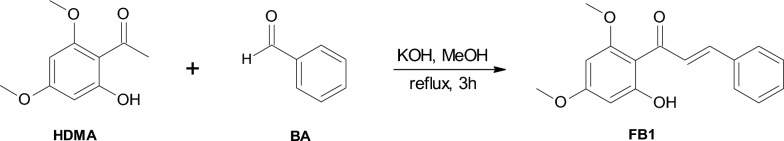
Flavokawain B synthesis

Flavokawain B (FB1) was obtained in the Claisen-Schmidt reaction of benzaldehyde (BA) with 2-hydroxy-4,6-dimetoxyacetophenone (HDMA) in the reaction presented in Fig. 6.

After 2 h of reflux, the product of the Claisen-Schmidt reaction was transferred to an acid environment and filtered using a Buchner funnel. The obtained compound was confirmed by NMR (^1^H NMR, ^13^C NMR, COSY, HMBC and HSQC) analysis.

### Microorganisms

The microorganisms *B. caledonica* KCh J3.3, *B. bassiana* KCh J1.5, KCh J1 and KCh BBT, *I. farinosa* KCh KW 1.1, *I. fumosorosea* KCh J2, *I. tenuipes* MU35 and *M. robertsii* MU4 were obtained from the collection of the Department of Food Chemistry and Biocatalysis, Wrocław University of Environmental and Life Sciences (Wrocław, Poland).

### Screening procedure

Erlenmeyer flasks (300 mL), each containing 100 mL of the sterile cultivation medium (3% glucose, 1% Aminobac), were inoculated with a suspension of each entomopathogenic strain and then incubated for 4 days at 24 °C on a rotary shaker. After this time, 10 mg of a substrate was dissolved in 1 mL of dimethyl sulfoxide (DMSO) and added to the medium. Samples were collected on the 1st, 3rd, 7th and 10th day of the process. Then, all products were extracted using ethyl acetate, and extracts were dried using MgSO_4_, concentrated *in vacuo* and analyzed using TLC and UHPLC methods.

### Scale-up biotransformation

For the scale-up process we used Erlenmeyer flasks (2000 mL), each containing 500 mL of the same cultivation medium (3% glucose, 1% Aminobac), which were inoculated in the same way as described above. Three days after inoculation, 100 mg of a substrate was dissolved in 2 mL of DMSO and added to the interior. Samples were collected on the 3–7th day of the process. Products were extracted three times using ethyl acetate and then analyzed using TLC, HPLC and NMR spectroscopy (^1^H NMR, ^13^C NMR, COSY, HMBC and HSQC) analysis.

### Analysis

Initial tests were carried out using TLC plates (SiO_2_, DC Alufolien Kieselgel 60 F_254_ (0.2 mm thick), Merck, Darmstadt, Germany). The mobile phase contained a mixture of chloroform and methanol in a 9:1 (*v*/*v*) ratio. The plates were observed using a UV lamp (254 and 365 nm).

The scale-up biotransformation products were separated using 500 µm preparative TLC silica gel plates (Anatech, Gehrden, Germany). The mobile phase contained a mixture of chloroform and methanol in a 9:1 (*v*/*v*) ratio. Separation products were scraped out and extracted twice using ethyl acetate.

### UHPLC

UHPLC analyses were carried out using a Thermo Scientific Dionex Ultimate 3000 UHPLC + instrument with a photodiode array detector (detection in wavelength: 210–450 nm) and a ZORBAX Eclipse XDB C-18 analytical column (5 m, 4.6 250 mm, Agilent, Santa Clara, CA, USA). Chromatographic separation was achieved using a gradient program as follows: initial conditions–25% B in A, 0.5 min–25% B in A, 4 min–40% B in A, 7.5 min–55% B in A, 8 min–100% B in A, 9.7 mi–25% B in A, 11 min–25% B in A. The flow rate was 0.7 mL min^−1^, where A: 0.1% HCOOH in H_2_O; B: 0.1% HCOOH in acetonitrile.

### NMR data and isolated yields of biotransformation products

Flavokawain B (FB1): ^1^H NMR (400 MHz, DMSO-*d*_6_) δ (ppm): 13.41 (s, 1H, C-2ʹ-OH); 7.76 (d, 1H, H-3, *J* = 15.7 Hz); 7.73–7.71 (m, 2H, H-2ʹʹ and H-6ʹʹ); 7.64 (d, 1H, H-2, *J* = 15.7 Hz); 7.48–7.39 (m, 3H, H-3ʹʹ, H-4ʹʹ and H-5ʹʹ); 6.16 (d, 1H, H-5ʹ, *J* = 2.3 Hz); 6.13 (d, 1H, H-3ʹ, *J* = 2.3 Hz); 3.89 (s, 3H, C-6ʹ-OCH_3_); 3.82 (s, 3H, C-4ʹ-OCH_3_); ^13^C NMR (101 MHz, DMSO-*d*_6_) δ (ppm): 192.39 (C-1); 165.65 (C-2ʹ); 165.52 (C-4ʹ);161.96 (C-6ʹ); 142.43 (C-3); 134.83 (C-1ʹʹ); 130.54 (C-4ʹʹ); 129.14 (C-3ʹʹ and C-5ʹʹ); 128.54 (C-2ʹʹ and C-6ʹʹ); 127.51 (C-2); 106.38 (C-1ʹ); 93.94 (C-3ʹ); 91.18 (C-5ʹ); 56.29 (C-6ʹ-OCH_3_); 55.76 (C-4ʹ-OCH_3_).

Cardamonin (FB2): After 7 days’ transformation of 100 mg of (FB1) in the *Isaria tenuipes* MU35 culture the isolation yield of (FB2) was 7 mg. ^1^H NMR (400 MHz, DMSO-*d*_6_) δ (ppm): 13.72 (s, 1H, C-2ʹ-OH);10.68 (s, 1H, C-4ʹ-OH); 7.83 (d, 1H, H-3, *J* = 15.7 Hz); 7.70–7.74 (m, 2H, H-2ʹʹ and H-6ʹʹ); 7.66 (d, 1H, H-2, *J* = 15.7 Hz); 7.43–7.48 (m, 3H, H-3ʹʹ, H-4ʹʹ and H-5ʹʹ); 6.02 (d, 1H, H-5ʹ, *J* = 2.3 Hz); 5.93 (d, 1H, H-3ʹ, *J* = 2.3 Hz); 3.88 (s, 3H, C-6ʹ-OCH_3_); (400 MHz, CDCl_3_) δ (ppm): 14.17 (s, 1H, C-2ʹ-OH); 8.63 (s, 1H, C-4ʹ-OH); 7.89 (d, 1H, H-3, *J* = 15.6 Hz); 7.77 (d, 1H, H-2, *J* = 15.6 Hz); 7.58–7.62 (m, 2H, H-2ʹʹ and H-6ʹʹ); 7.36–7.43 (m, 3H, H-3ʹʹ, H-4ʹʹ and H-5ʹʹ); 6.05 (d, 1H, H-5ʹ, *J* = 2.1 Hz); 5.97 (d, 1H, H-3ʹ, *J* = 2.3 Hz); 3.92 (s, 3H, C-6ʹ-OCH_3_).

4ʹ-*O*-β-D-(4″-*O*-methylglucopyranosyl)-cardamonin (FB3). After 7 days’ transformation of 100 mg of (FB1) in the *Metarhizium robertsii* MU4 culture the isolation yield of (FB3) was 42 mg. ^1^H NMR (400 MHz, DMSO*-d*_6_) δ (ppm): 7.70–7.76 (m, 2H, H-2ʹʹ and H-6ʹʹ); 7.67 (d, 1H, H-2, *J* = 15.8 Hz); 7.60 (d, 1H, H-3, *J* = 15.7 Hz); 7.42–7.48 (m, 3H, H-3ʹʹ, H-4ʹʹ and H-5ʹʹ); 6.25 (d, 1H, H-5ʹ, *J* = 2.1 Hz); 6.21 (d, 1H, H-3ʹ, *J* = 2.2 Hz); 5.45 (broad s, 1H, C-2ʹʹʹ-OH); 5.31 (broad s, 1H, C-3ʹʹʹ-OH); 5.02 (d, 1H, H-1ʹʹʹ, *J* = 7.8 Hz); 4.76 (t,1H, C-6ʹʹʹ-OH, *J* = 5.4 Hz); 3.87 (s, 3H, C-6ʹ-OCH_3_); 3.60–3.68 (m, 1H, one of H-6ʹʹʹ); 3,47–3,54 (m, 1H, one of H-6ʹʹʹ); 3.45 (s, 3H, C-4ʹʹʹ-OCH_3_); 3.42–3.46 (m, 1H, H-3ʹʹʹ); 3,32–3,36 (m, 1H, H-5ʹʹʹ); 3.24 (t, 1H, H-2ʹʹʹ, *J* = 7.9 Hz); 3.02 (t, 1H, H-4ʹʹʹ, *J* = 9.2 Hz); ^13^C NMR (151 MHz, DMSO*-d*_6_) δ (ppm): 192.71 (C-1); 163.85 (C-2ʹ); 162.88 (C-4ʹ); 161.45 (C-6ʹ); 142.71 (C-3); 134.72 (C-1ʹʹ); 130.55 (C-4ʹʹ); 129.10 (C-2ʹʹ and C-6ʹʹ); 128.55 (C-3ʹʹ and C-5ʹʹ); 127.64 (C-2); 107.54 (C-1ʹ); 99,36 (C-1ʹʹʹ); 96.33 (C-3ʹ); 92.18 (C-5ʹ); 79.00 (C-4ʹʹʹ); 76.24 (C-3ʹʹʹ); 75.76 (C-5ʹʹʹ); 73.29 (C-2ʹʹʹ); 60.19 (C-6ʹʹʹ); 59.72 (C-4ʹʹʹ-OCH_3_); 56.20 (C-6ʹ-OCH_3_).

4ʹ-*O*-β-D-(4″-*O*-methylglucopyranosyl)-3ʹʹ-hydroxycardamonin (FB4): After 3 days’ transformation of 100 mg of (FB1) in the *Beauveria bassiana* KCh BBT culture the isolation yield of (FB4) was 12 mg. ^1^H NMR (400 MHz, DMSO*-d*_6_) δ (ppm): 12.94 (s, 1H, C-2ʹ-OH); 9.68 (s, 1H, C-3ʹʹ-OH); 7.60 (d, 1H, H-2, *J* = 15.5 Hz); 7.52 (d, 1H, H-3, *J* = 15.7 Hz); 7.25 (t, 1H, H-5ʹʹ, *J* = 7.8 Hz); 7.13 (d, 1H, H-6ʹʹ, *J* = 7.8 Hz); 7.07 (broad s, 1H, H-2ʹʹ); 6.85 (dd, 1H, H-4ʹʹ, *J* = 7.8, 1.8 Hz); 6.25 (d, 1H, H-5ʹ, *J* = 2.1 Hz); 6.20 (d, 1H, H-3ʹ, *J* = 2.1 Hz); 5.46 (d, 1H, C-2ʹʹʹ-OH, *J* = 5.2 Hz); 5.32 (d, 1H, H-3ʹʹʹ-OH, *J* = 5.5 Hz); 5.02 (d, 1H, H-1ʹʹʹ, *J* = 7.8 Hz); 4.77 (t, 1H, C-6ʹʹʹ-OH, *J* = 5.5 Hz); 3.88 (s, 3H, C-6ʹ-OCH_3_); 3.64 (dd, 1H, one of H-6ʹʹʹ, *J* = 11.4, 3.6 Hz); 3.45 (s, 3H, C-4ʹʹʹ-OCH_3_); 3.42–3.54 (m, 2H, H-3ʹʹʹ and one of H-6ʹʹʹ); 3.32–3.36 (m, 1H, H-5ʹʹʹ); 3.23–3.28 (m, 1H, H-2ʹʹʹ); 3.19 (t, 1H, H-4ʹʹʹ, *J* = 9.2 Hz); ^13^C NMR (151 MHz, DMSO d_6_) δ (ppm): 192.62 (C-1); 166.21 (C-2ʹ); 162.98 (C-4ʹ); 161.54 (C-6ʹ); 157.80 (C-3ʹʹ); 142.93 (C-3); 136.00 (C-1ʹʹ); 130.15 (C-5ʹʹ); 127.37 (C-2); 119.78 (C-6ʹʹ); 117.83 (C-4ʹʹ); 114.45 (C-2ʹʹ); 107.43 (C-1ʹ); 99.35 (C-1ʹʹʹ); 96.36 (C-3ʹ); 92.23 (C-5ʹ); 79.02 (C-4ʹʹʹ); 75.78 (C-3ʹʹʹ); 75.59 (C-5ʹʹʹ); 73.30 (C-2ʹʹʹ); 60.21 (C-6ʹʹʹ); 59.75 (C-4ʹʹʹ, -OCH_3_); 56.22 (C-6ʹ, -OCH_3_).

4ʹ-*O*-β-D-(4″-*O*-methylglucopyranosyl)-4ʹʹ-hydroxycardamonin (FB5): After 7 days’ transformation of 100 mg of (FB1) in the *Metarhizium robertsii* MU4 culture the isolation yield of (FB5) was 11 mg. ^1^H NMR (400 MHz, acetone*-d*_6_) δ (ppm): 7.87 (d, 1H, H-2, *J* = 15.5 Hz); 7.76 (d, 1H, H-3, *J* = 15.5 Hz); 7.60–7.65 (m, 2H, H-2ʹʹ and H-6ʹʹ); 6.88–6.96 (m, 2H, H-3ʹʹ and H-5ʹʹ); 6.27 (d, 1H, H-5ʹ, *J* = 2.2 Hz); 6.18 (d, 1H, H-3ʹ, *J* = 2.2 Hz); 5.09 (d, 1H, H-1ʹʹʹ, *J* = 7.8 Hz); 4.01 (s, 3H, C-6ʹ-OCH_3_); 3.85 (dd, 1H, one of H-6ʹʹʹ, *J* = 12.0, 2.0 Hz); 3.69 (dd, 1H, one of H-6ʹʹʹ, *J* = 11.8, 5.0 Hz); 3.65 (t, 1H, H-3ʹʹʹ, *J* = 9.0 Hz); 3.57–3.60 (m, 1H, H-5ʹʹʹ); 3.56 (s, 3H, C-4ʹʹʹ-OCH_3_); 3.47 (dd, 1H, H-2ʹʹʹ, *J* = 9.1, 7.8 Hz);3.19 (dd, 1H, H-4ʹʹʹ, *J* = 9.5, 9.1 Hz); ^13^C NMR (151 MHz, acetone-*d*_6_) δ (ppm): 193.55 (C-1); 167.72 (C-2ʹ); 164.81 (C-4ʹ); 163.59 (C-6ʹ); 160.67 (C-4ʹʹ); 143.88 (C-3); 131.39 (C-2ʹʹ and C-6ʹʹ); 127.86 (C-1ʹʹ); 125.02 (C-2); 116.72 (C-3ʹʹ and C-5ʹʹ); 107.62 (C-1ʹ); 100.64 (C-1ʹʹʹ); 97.48 (C-3ʹ); 92.81 (C-5ʹ); 80.04 (C-4ʹʹʹ); 77.77 (C-3ʹʹʹ); 77.19 (C-5ʹʹʹ); 74.62 (C-2ʹʹʹ); 61.91 (C-6ʹʹʹ); 60.53 (C-4ʹʹʹ-OCH_3_); 56.52 (C-6ʹ-OCH_3_).

3ʹʹ-hydroxyflavokawain B (FB6): After 3 days’ transformation of 100 mg of (FB1) in the *Beauveria caledonica* KCh J3.3 culture the isolation yield of (FB6) was 1 mg. ^1^H NMR (400 MHz, CDCl_3_) δ (ppm): 14.25 (s, 1H, H-2ʹ, -OH); 7.86 (d, 1H, H-3, *J* = 15.6 Hz); 7.71 (d, 1H, H-2, *J* = 15.6 Hz); 7.28 (t, 1H, H-5ʹ, *J* = 7.9 Hz); 7.18 (d, 1H, H-6ʹ, *J* = 7.7 Hz); 7.07 (s, 1H, H-2ʹ); 6.87 (dd, 1H, H-4ʹ, *J* = 8.0, 1.7 Hz); 6.11 (d, 1H, H-5ʹ, *J* = 2.4 Hz); 5.97 (d, 1H, H-3ʹ, *J* = 2.4 Hz); 3.92 (s, 3H, C-6ʹ-OCH_3_); 3.84 (s, 3H, C-4ʹ-OCH_3_).

3ʹ-*O*-β-D-(4″-*O*-methylglucopyranosyl)-flavokawain B (FB7): After 7 days’ transformation of 100 mg of (FB1) in the *Beauveria bassiana* KCh J1.5 culture the isolation yield of (FB7) was 25 mg. ^1^H NMR (400 MHz, DMSO*-d*_6_) δ (ppm): 13.35 (s, 1H, C-2ʹ-OH); 7.73 (d, 1H, H-2, *J* = 15.7 Hz); 7.61 (d, 1H, H-3, *J* = 15.7 Hz); 7.33–7.39 (m, 3H, H-2ʹʹ, 5ʹʹ and H-6ʹʹ); 7.07–7.11 (m, 1H, H-4ʹʹ); 6.17 (d, 1H, H-5ʹ, *J* = 2.3 Hz); 6.14 (d, 1H, H-3ʹ, *J* = 2.3 Hz); 5.42 (d, 1H, C-2ʹʹʹ-OH, *J* = 5.2 Hz); 5.28 (d, 1H, C-3ʹʹʹ-OH, *J* = 5.2 Hz); 4.99 (d, 1H, H-1ʹʹʹ, *J* = 7.8 Hz); 4.73 (dd, 1H, C-6ʹʹʹ-OH, *J* = 6.1, 5.2 Hz); 3.89 (s, 3H, C-6ʹ-OCH_3_); 3.83 (s, 3H, C-4ʹ-OCH_3_); 3.64 (ddd, 1H, one of H-6ʹʹʹ, *J* = 11.8, 5.2, 1.5 Hz); 3.52 (dd, 1H, one of H-6ʹʹʹ, *J* = 11.7, 5.1 Hz); 3.46 (s, 3H, C-4ʹʹʹ-OCH_3_); 3.40–4.48 (m, 2H, H-3ʹʹʹ and H-5ʹʹʹ); 3.26 (ddd, 1H, H-2ʹʹʹ, *J* = 8.9, 8.2, 5.2 Hz); 3.04 (t, 1H, H-4ʹʹʹ, *J* = 9.3 Hz); ^13^C NMR (151 MHz, DMSO*-d*_*6*_) δ (ppm): 192.34 (C-1); 165.66 (C-4ʹ); 165.44 (C-2ʹ); 161.95 (C-6ʹ); 157.72 (C-3ʹʹ); 142.10 (C-3); 136.18 (C-1ʹʹ); 130.07 (C-5ʹʹ); 127.86 (C-2); 122.06 (C-6ʹʹ); 118.45 (C-4ʹʹ); 115.68 (C-2ʹʹ); 106.39 (C-1ʹ); 99.89 (C-1ʹʹʹ); 93.88 (C-3ʹ); 91.17 (C-5ʹ); 79.00 (C-4ʹʹʹ); 76.28 (C-3ʹʹʹ); 75.56 (C-5ʹʹʹ); 73.44 (C-2ʹʹʹ); 60.23 (C-6ʹʹʹ); 59.70 (C-4ʹʹʹ-OCH_3_); 56.28 (C-6ʹ-OCH_3_); 55.74 (C-4ʹ-OCH_3_).

3ʹ-*O*-β-D-(4″-*O*-methylglucopyranosyl)-4ʹʹ-hydroxyflavokawain B (FB8): After 7 days’ transformation of 100 mg of (FB1) in the *Beauveria bassiana* KCh J1.5 culture the isolation yield of (FB8) was 25 mg. ^1^H NMR (400 MHz, DMSO*-d*_6_) δ (ppm): 13.60 (s, 1H, C-2ʹ-OH); 8.91 (s, 1H, C-4ʹʹ, -OH); 7.62 (d, 1H, H-2, *J* = 15.6 Hz); 7.56 (d, 1H, H-3, *J* = 15.6 Hz); 7.18 (d, 1H, H-2ʹʹ, *J* = 1.5 Hz); 7.14 (dd, 1H, H-6ʹʹ, *J* = 8.7, 1.5 Hz); 7.11 (d, 1H, H-5ʹʹ, *J* = 8.6 Hz); 6.16 (d, 1H, H-5ʹ, *J* = 2.3 Hz); 6.12 (d, 1H, H-3ʹ, *J* = 2.3 Hz); 5.45 (d, 1H, C-2ʹʹʹ-OH, *J* = 4.2 Hz); 5.28 (d, 1H, C-3ʹʹʹ-OH, *J* = 5.4 Hz); 4.99 (d, 1H, H-1ʹʹʹ, *J* = 7.8 Hz); 4.75 (dd, 1H, C-6ʹʹʹ-OH, *J* = 6.2, 5.2 Hz); 3.90 (s, 3H, C-6ʹ-OCH_3_); 3.82 (s, 3H, C-4ʹ-OCH_3_); 3.64 (ddd, 1H, one of H-6ʹʹʹ, *J* = 11.1, 4.6, 1.5 Hz); 3.48–3.54 (m, 1H, one of, H-6ʹʹʹ); 3.46 (s, 3H, C-4ʹʹʹ-OCH_3_); 3.39–4.47 (m, 2H, H-3ʹʹʹ and H-5ʹʹʹ); 3.27–3.33 (m, 1H, H-2ʹʹʹ); 3.05 (t, 1H, H-4ʹʹʹ, *J* = 9.2 Hz); ^13^C NMR (151 MHz, DMSO*-d*_*6*_) δ(ppm): 192.17 (C-1); 165.73 (C-4ʹ); 165.51 (C-2ʹ); 161.92 (C-6ʹ); 146.88 (C-4ʹʹ); 142.83 (C-3); 129.29 (C-1ʹʹ); 125.42 (C-2); 121.34 (C-6ʹʹ); 115.93 (C-5ʹʹ); 114.86 (C-2ʹʹ); 106.23 (C-1ʹ); 100.94 (Cʹʹʹ-1); 93.91 (C-3ʹ); 91.15 (C-5ʹ); 79.04 (C-4ʹʹʹ); 75.74 (C-3ʹʹʹ); 75.62 (C-5ʹʹʹ); 73.42 (C-2ʹʹʹ); 60.24 (C-6ʹʹʹ); 59.76 (C-4ʹʹʹ-OCH_3_); 56.24 (C-6ʹ-OCH_3_); 55.71 (C-4ʹ-OCH_3_).

## Conclusions

In summary, the study explored the enzymatic modification of flavokawain B (FB1) within the entomopathogenic microbial cultures of *B. bassiana*, *B. caledonica*, *I. fumosorosea*, *I. farinosa* and *M. robertsii* strains. The investigation revealed the biotransformation of flavokawain B (FB1) into seven identified derivatives through the catalytic activities of the selected filamentous fungal strains.

The primary findings included the predominant occurrence of 4-*O*-methylglycosylation in the culture of described strains, each exhibiting distinct patterns of biotransformation. The biotransformation products were characterized using various analytical techniques, including nuclear magnetic resonance (NMR) spectroscopy and high-performance liquid chromatography (HPLC).

*M. robertsii* MU4, for instance, exhibited the formation of 4ʹ-*O*-β-D-(4ʹʹ-*O*-methylglucopyranosyl)-cardamonin (FB3) as the main product, indicating a process of demethylation and 4-*O*-methylglycosylation. However, in cultures of *B*. *bassiana* KCh BBT and KCh J1.5 strains, transformations within the B ring were observed; the attachment of the sugar unit was preceded by hydroxylation.

Cheminformatics tools were employed to compare the physicochemical characteristics, pharmacokinetics, and potential biological activities of flavokawain B (FB1) and its derivatives. The results indicated that the modified derivatives generally displayed lower lipophilicity than the parent compound. Moreover, the introduction of a sugar unit to the chalcone core affected the compoundsʹ absorption from the gastrointestinal tract.

Predictions from ADME factors, pharmacokinetic characteristics, and biological activities suggested that the derivatives, in most cases, exhibited comparable or enhanced activities compared to flavokawain B (FB1). Notably, the products with methylglycosyl residues were predicted to have a higher probability of anticarcinogenic activity, and all derivatives were expected to stimulate caspase 3, leading to cell apoptosis. Furthermore, the in silico studies predicted antileishmanial activity for the derivatives, aligning with the broader knowledge of chalcones and their analogs demonstrating antiprotozoal effects.

In conclusion, this study has provided noteworthy insights into the enzymatic transformations undergone by flavokawain B (FB1) through the actions of entomopathogenic filamentous fungi. It effectively elucidated the structural modifications and furnished predictions regarding the potential pharmacological activities of the resultant compounds. These findings significantly enhance our understanding of the biocatalytic capabilities inherent in these microbial cultures, emphasizing the promising therapeutic applications of the modified flavokawain B derivatives.

### Supplementary Information

**Additional file 1.** Supplementary Figures S1–S64.

## Data Availability

All data generated or analyzed during this study are included in this published article and its Additional file.
