# Supplementary material for: Multienzymatic biotransformation of flavokawain B by entomopathogenic filamentous fungi: structural modifications and pharmacological predictions
Source: Microb Cell Fact. 2024 Feb 24;23:65. doi: 10.1186/s12934-024-02338-9 (PMC10893614; doi:10.1186/s12934-024-02338-9)
Supplement: Supplementary file 1 — Additional file 1. Supplementary Figures S1–S64. [file 12934_2024_2338_MOESM1_ESM.pdf]

Supplementary Data

Multienzymatic biotransformation of flavokawain B by entomopathogenic filamentous fungi: structural modifications and pharmacological predictions

Paweł Chlipała[1*], Tomasz Tronina[1], Monika Dymarska[1], Monika Urbaniak[2], Ewa Kozłowska[1], Łukasz Stępień[2], Edyta Kostrzewa-Susłow[1] and Tomasz Janeczko[1*]

[1] Department of Food Chemistry and Biocatalysis, Wrocław University of Environmental and Life Sciences, Wrocław, Poland; tomasz.tronina@upwr.edu.pl (T.T.); monika.dymarska@upwr.edu.pl (M.D.) e.a.kozlowska@gmail.com (E.K.); edyta.kostrzewa-suslow@upwr.edu.pl (E.K.-S.)
[2] Institute of Plant Genetics, Polish Academy of Sciences, Strzeszyńska 34, 60-479 Poznań, Poland; murb@igr.poznan.pl (M.U.); lste@igr.poznan.pl (Ł.S.)
[*] Corresponding author: pawel.chlipala@upwr.edu.pl (P.C.); janeczko13@interia.pl (T.J.)

Contents:

Figure S1. The UV absorption maxima of 1-(2'-hydroxy-4',6'-dimethoxyphenyl)-3-phenyl-prop-2-en-1-on - flavokawain B (**FB1**)

Figure S2. ^{1}H NMR spectra of 1-(2'-hydroxy-4',6'-dimethoxyphenyl)-3-phenyl-prop-2-en-1-on - flavokawain B (**FB1**) (DMSO-d_6, 600 MHz)

Figure S3. Flavone part of the ^{1}H NMR spectral 1-(2'-hydroxy-4',6'-dimethoxyphenyl)-3-phenyl-prop-2-en-1-on - flavokawain B (**FB1**) (DMSO-d_6, 600 MHz)

Figure S4. ^{13}C NMR spectra of 1-(2'-hydroxy-4',6'-dimethoxyphenyl)-3-phenyl-prop-2-en-1-on - flavokawain B (**FB1**) (DMSO-d_6, 151 MHz)

Figure S5. COSY spectrum of 1-(2'-hydroxy-4',6'-dimethoxyphenyl)-3-phenyl-prop-2-en-1-on - flavokawain B (**FB1**) (DMSO-d_6, 600 MHz)

Figure S6. HSQC spectrum of 1-(2'-hydroxy-4',6'-dimethoxyphenyl)-3-phenyl-prop-2-en-1-on - flavokawain B (**FB1**) (DMSO-d_6, 600/151 MHz)

Figure S7. HMBC spectrum of 1-(2'-hydroxy-4',6'-dimethoxyphenyl)-3-phenyl-prop-2-en-1-on - flavokawain B (**FB1**) (DMSO-d_6, 600/151 MHz)

Figure S8. Predicted Boiled-Egg plot from swissADME online web tool for 1-(2'-hydroxy-4',6'-dimethoxyphenyl)-3-phenyl-prop-2-en-1-on - flavokawain B (**FB1**)

Figure S9. 1-(2'-hydroxy-4',6'-dimethoxyphenyl)-3-phenyl-prop-2-en-1-on - flavokawain B (**FB1**) physicochemical and ADME parameters prediction using the SwissADME modelling

Figure S10. The UV absorption maxima of 1-(2',4'-dihydroxy-6'-methoxyphenyl)-3-phenyl-prop-2-en-1-on - cardamonin (**FB2**)

Figure S11. ^{1}H NMR spectra of 1-(2',4'-dihydroxy-6'-methoxyphenyl)-3-phenyl-prop-2-en-1-on - cardamonin (**FB2**) (DMSO-d_6, 600 MHz)

Figure S12. Flavone part of the ^{1}H NMR spectral 1-(2',4'-dihydroxy-6'-methoxyphenyl)-3-phenyl-prop-2-en-1-on - cardamonin (**FB2**) (DMSO-d_6, 600 MHz)

Figure S12. Predicted Boiled-Egg plot from swissADME online web tool for 1-(2',4'-dihydroxy-6'-methoxyphenyl)-3-phenyl-prop-2-en-1-on - cardamonin (**FB2**)

Figure S14. 1-(2',4'-dihydroxy-6'-methoxyphenyl)-3-phenyl-prop-2-en-1-on - cardamonin (**FB2**) physicochemical and ADME parameters prediction using the SwissADME modelling

Figure S15. The UV absorption maxima of 1-(4'-*O*-β-D-(4'''-*O*-methylglucopiranosyl)-2'-hydroxy-6'-methoxyphenyl)-3-phenyl-prop-2-en-1-on - 4'-*O*-β-D-(4''-*O*-methylglucopyranosyl)-cardamonin (**FB3**)

Figure S16. ^{1}H NMR spectra of 1-(4'-*O*-β-D-(4'''-*O*-methylglucopiranosyl)-2'-hydroxy-6'-methoxyphenyl)-3-phenyl-prop-2-en-1-on - 4'-*O*-β-D-(4''-*O*-methylglucopyranosyl)-cardamonin (**FB3**) (DMSO-d_6, 600 MHz)

Figure S17. Flavone part of the ^{1}H NMR spectral 1-(4'-*O*-β-D-(4'''-*O*-methylglucopiranosyl)-2'-hydroxy-6'-methoxyphenyl)-3-phenyl-prop-2-en-1-on - 4'-*O*-β-D-(4''-*O*-methylglucopyranosyl)-cardamonin (**FB3**) (DMSO-d_6, 600 MHz)

Figure S18. ^{13}C NMR spectra of 1-(4'-*O*-β-D-(4'''-*O*-methylglucopiranosyl)-2'-hydroxy-6'-methoxyphenyl)-3-phenyl-prop-2-en-1-on - 4'-*O*-β-D-(4''-*O*-methylglucopyranosyl)-cardamonin (**FB3**) (DMSO-d_6, 151 MHz)

Figure S19. COSY spectrum of 1-(4'-*O*-β-D-(4'''-*O*-methylglucopiranosyl)-2'-hydroxy-6'-methoxyphenyl)-3-phenyl-prop-2-en-1-on - 4'-*O*-β-D-(4''-*O*-methylglucopyranosyl)-cardamonin (**FB3**) (DMSO-d_6, 600 MHz)

Figure S20. HSQC spectrum of 1-(4'-*O*-β-D-(4'''-*O*-methylglucopiranosyl)-2'-hydroxy-6'-methoxyphenyl)-3-phenyl-prop-2-en-1-on - 4'-*O*-β-D-(4''-*O*-methylglucopyranosyl)-cardamonin (**FB3**) (DMSO-d_6, 600/151 MHz)

Figure S21. HMBC spectrum of 1-(4'-*O*-β-D-(4'''-*O*-methylglucopiranosyl)-2'-hydroxy-6'-methoxyphenyl)-3-phenyl-prop-2-en-1-on - 4'-*O*-β-D-(4''-*O*-methylglucopyranosyl)-cardamonin (**FB3**) (DMSO-d_6, 600/151 MHz)

Figure S22. Predicted Boiled-Egg plot from swissADME online web tool for 1-(4'-*O*-β-D-(4'''-*O*-methylglucopiranosyl)-2'-hydroxy-6'-methoxyphenyl)-3-phenyl-prop-2-en-1-on - 4'-*O*-β-D-(4''-*O*-methylglucopyranosyl)-cardamonin (**FB3**)

Figure S23. 1-(4'-*O*-β-D-(4'''-*O*-methylglucopiranosyl)-2'-hydroxy-6'-methoxyphenyl)-3-phenyl-prop-2-en-1-on - 4'-*O*-β-D-(4''-*O*-methylglucopyranosyl)-cardamonin (**FB3**) physicochemical and ADME parameters prediction using the SwissADME modelling

Figure S24. The UV absorption maxima of 1-(4'-*O*-β-D-(4'''-*O*-methylglucopiranosyl)-2'-hydroxy-6'-methoxyphenyl)-3-(3''-hydoxyphenyl)-prop-2-en-1-on - 4'-*O*-β-D-(4″-*O*-methylglucopyranosyl)-3''-hydroxycardamonin (**FB4**)

Figure S25. ^{1}H NMR spectra of 1-(4'-*O*-β-D-(4'''-*O*-methylglucopiranosyl)-2'-hydroxy-6'-methoxyphenyl)-3-(3''-hydoxyphenyl)-prop-2-en-1-on - 4'-*O*-β-D-(4″-*O*-methylglucopyranosyl)-3''-hydroxycardamonin (**FB4**) (DMSO-d_6, 600 MHz)

Figure S26. Flavone part of the ^{1}H NMR spectral 1-(4'-*O*-β-D-(4'''-*O*-methylglucopiranosyl)-2'-hydroxy-6'-methoxyphenyl)-3-(3''-hydoxyphenyl)-prop-2-en-1-on - 4'-*O*-β-D-(4″-*O*-methylglucopyranosyl)-3''-hydroxycardamonin (**FB4**) (DMSO-d_6, 600 MHz)

Figure S27. ^{13}C NMR spectra of 1-(4'-*O*-β-D-(4'''-*O*-methylglucopiranosyl)-2'-hydroxy-6'-methoxyphenyl)-3-(3''-hydoxyphenyl)-prop-2-en-1-on - 4'-*O*-β-D-(4″-*O*-methylglucopyranosyl)-3''-hydroxycardamonin (**FB4**) (DMSO-d_6, 151 MHz)

Figure S28. COSY spectrum of 1-(4'-*O*-β-D-(4'''-*O*-methylglucopiranosyl)-2'-hydroxy-6'-methoxyphenyl)-3-(3''-hydoxyphenyl)-prop-2-en-1-on - 4'-*O*-β-D-(4″-*O*-methylglucopyranosyl)-3''-hydroxycardamonin (**FB4**) (DMSO-d_6, 600 MHz)

Figure S29. HSQC spectrum of 1-(4'-*O*-β-D-(4'''-*O*-methylglucopiranosyl)-2'-hydroxy-6'-methoxyphenyl)-3-(3''-hydoxyphenyl)-prop-2-en-1-on - 4'-*O*-β-D-(4″-*O*-methylglucopyranosyl)-3''-hydroxycardamonin (**FB4**) (DMSO-d_6, 600/151 MHz)

Figure S30. HMBC spectrum of 1-(4'-*O*-β-D-(4'''-*O*-methylglucopiranosyl)-2'-hydroxy-6'-methoxyphenyl)-3-(3''-hydoxyphenyl)-prop-2-en-1-on - 4'-*O*-β-D-(4″-*O*-methylglucopyranosyl)-3''-hydroxycardamonin (**FB4**) (DMSO-d_6, 600/151 MHz)

Figure S31. Predicted Boiled-Egg plot from swissADME online web tool for 1-(4'-*O*-β-D-(4'''-*O*-methylglucopiranosyl)-2'-hydroxy-6'-methoxyphenyl)-3-(3''-hydoxyphenyl)-prop-2-en-1-on - 4'-*O*-β-D-(4″-*O*-methylglucopyranosyl)-3''-hydroxycardamonin (**FB4**)

Figure S32. 1-(4'-*O*-β-D-(4'''-*O*-methylglucopiranosyl)-2'-hydroxy-6'-methoxyphenyl)-3-(3''-hydoxyphenyl)-prop-2-en-1-on - 4'-*O*-β-D-(4″-*O*-methylglucopyranosyl)-3''-hydroxycardamonin (**FB4**) physicochemical and ADME parameters prediction using the SwissADME modelling

Figure S33. The UV absorption maxima of 1-(4'-*O*-β-D-(4'''-*O*-methylglucopiranosyl)-2'-hydroxy-6'-methoxyphenyl)-3-(4''-hydoxyphenyl)-prop-2-en-1-on - 4'-*O*-β-D-(4″-*O*-methylglucopyranosyl)-4''-hydroxycardamonin (**FB5**)

Figure S34. ^{1}H NMR spectra of 1-(4'-*O*-β-D-(4'''-*O*-methylglucopiranosyl)-2'-hydroxy-6'-methoxyphenyl)-3-(4''-hydoxyphenyl)-prop-2-en-1-on - 4'-*O*-β-D-(4″-*O*-methylglucopyranosyl)-4''-hydroxycardamonin (**5**) (Acetone-d_6, 600 MHz)

Figure S35. Flavone part of the ^{1}H NMR spectral 1-(4'-*O*-β-D-(4'''-*O*-methylglucopiranosyl)-2'-hydroxy-6'-methoxyphenyl)-3-(4''-hydoxyphenyl)-prop-2-en-1-on - 4'-*O*-β-D-(4″-*O*-methylglucopyranosyl)-4''-hydroxycardamonin (**FB5**) (Acetone-d_6, 600 MHz)

Figure S36. ^{13}C NMR spectra of 1-(4'-*O*-β-D-(4'''-*O*-methylglucopiranosyl)-2'-hydroxy-6'-methoxyphenyl)-3-(4''-hydoxyphenyl)-prop-2-en-1-on - 4'-*O*-β-D-(4″-*O*-methylglucopyranosyl)-4''-hydroxycardamonin (**FB5**) (Acetone-d_6, 151 MHz)

Figure S37. COSY spectrum of 1-(4'-*O*-β-D-(4'''-*O*-methylglucopiranosyl)-2'-hydroxy-6'-methoxyphenyl)-3-(4''-hydoxyphenyl)-prop-2-en-1-on - 4'-*O*-β-D-(4″-*O*-methylglucopyranosyl)-4''-hydroxycardamonin (**FB5**) (Acetone-d_6, 600 MHz)

Figure S38. HSQC spectrum of 1-(4'-*O*-β-D-(4'''-*O*-methylglucopiranosyl)-2'-hydroxy-6'-methoxyphenyl)-3-(4''-hydoxyphenyl)-prop-2-en-1-on - 4'-*O*-β-D-(4″-*O*-methylglucopyranosyl)-4''-hydroxycardamonin (**FB5**) (Acetone-d_6, 600/151 MHz)

Figure S39. HMBC spectrum of 1-(4'-*O*-β-D-(4'''-*O*-methylglucopiranosyl)-2'-hydroxy-6'-methoxyphenyl)-3-(4''-hydoxyphenyl)-prop-2-en-1-on - 4'-*O*-β-D-(4″-*O*-methylglucopyranosyl)-4''-hydroxycardamonin (**FB5**) (Acetone-d_6, 600/151 MHz)

Figure S40. Predicted Boiled-Egg plot from swissADME online web tool for 1-(4'-*O*-β-D-(4'''-*O*-methylglucopiranosyl)-2'-hydroxy-6'-methoxyphenyl)-3-(4''-hydoxyphenyl)-prop-2-en-1-on - 4'-*O*-β-D-(4″-*O*-methylglucopyranosyl)-4''-hydroxycardamonin (**FB5**)

Figure S41. 1-(4'-*O*-β-D-(4'''-*O*-methylglucopiranosyl)-2'-hydroxy-6'-methoxyphenyl)-3-(4''-hydoxyphenyl)-prop-2-en-1-on - 4'-*O*-β-D-(4″-*O*-methylglucopyranosyl)-4''-hydroxycardamonin (**FB5**) physicochemical and ADME parameters prediction using the SwissADME modelling

Figure S42. The UV absorption maxima of 1-(2'-hydroxy-4',6'-dimethoxyphenyl)-3-(3''-hydroxyphenyl)-prop-2-en-1-on - 3''-hydroxyflavokawain B (**FB6**)

Figure S43. ^{1}H NMR spectra of 1-(2'-hydroxy-4',6'-dimethoxyphenyl)-3-(3''-hydroxyphenyl)-prop-2-en-1-on - 3''-hydroxyflavokawain B (**FB6**) ($CDCl_3$, 600 MHz)

Figure S44. Flavone part of the ^{1}H NMR spectral 1-(2'-hydroxy-4',6'-dimethoxyphenyl)-3-(3''-hydroxyphenyl)-prop-2-en-1-on - 3''-hydroxyflavokawain B (**FB6**) ($CDCl_3$, 600 MHz)

Figure S45. Predicted Boiled-Egg plot from swissADME online web tool for 1-(2'-hydroxy-4',6'-dimethoxyphenyl)-3-(3''-hydroxyphenyl)-prop-2-en-1-on - 3''-hydroxyflavokawain B (**FB6**)

Figure S46. 1-(2'-hydroxy-4',6'-dimethoxyphenyl)-3-(3''-hydroxyphenyl)-prop-2-en-1-on - 3''-hydroxyflavokawain B (**FB6**) physicochemical and ADME parameters prediction using the SwissADME modelling

Figure S47. The UV absorption maxima of 1-(2'-hydroxy-4',6'-dimethoxyphenyl)-3-(3''-*O*-β-D-(4'''-*O*-methylglucopiranosyl)-phenyl)-prop-2-en-1-on - 3'-*O*-β-D-(4″-*O*-methylglucopyranosyl)-flavokawain B (**FB7**)

Figure S48. ^{1}H NMR spectra of 1-(2'-hydroxy-4',6'-dimethoxyphenyl)-3-(3''-*O*-β-D-(4'''-*O*-methylglucopiranosyl)-phenyl)-prop-2-en-1-on - 3'-*O*-β-D-(4″-*O*-methylglucopyranosyl)-flavokawain B (**FB7**) (DMSO-d_6, 600 MHz)

Figure S49. Flavone part of the ^{1}H NMR spectral 1-(2'-hydroxy-4',6'-dimethoxyphenyl)-3-(3''-*O*-β-D-(4'''-*O*-methylglucopiranosyl)-phenyl)-prop-2-en-1-on - 3'-*O*-β-D-(4″-*O*-methylglucopyranosyl)-flavokawain B (**FB7**) (DMSO-d_6, 600 MHz)

Figure S50. ^{13}C NMR spectra of 1-(2'-hydroxy-4',6'-dimethoxyphenyl)-3-(3''-*O*-β-D-(4'''-*O*-methylglucopiranosyl)-phenyl)-prop-2-en-1-on - 3'-*O*-β-D-(4″-*O*-methylglucopyranosyl)-flavokawain B (**FB7**) (DMSO-d_6, 151 MHz)

Figure S51. COSY spectrum of 1-(2'-hydroxy-4',6'-dimethoxyphenyl)-3-(3''-*O*-β-D-(4'''-*O*-methylglucopiranosyl)-phenyl)-prop-2-en-1-on - 3'-*O*-β-D-(4″-*O*-methylglucopyranosyl)-flavokawain B (**7**) (DMSO-d_6, 600 MHz)

Figure S52. HSQC spectrum of 1-(2'-hydroxy-4',6'-dimethoxyphenyl)-3-(3''-*O*-β-D-(4'''-*O*-methylglucopiranosyl)-phenyl)-prop-2-en-1-on - 3'-*O*-β-D-(4″-*O*-methylglucopyranosyl)-flavokawain B (**FB7**) (DMSO-d_6, 600/151 MHz)

Figure S53. HMBC spectrum of 1-(2'-hydroxy-4',6'-dimethoxyphenyl)-3-(3''-*O*-β-D-(4'''-*O*-methylglucopiranosyl)-phenyl)-prop-2-en-1-on - 3'-*O*-β-D-(4″-*O*-methylglucopyranosyl)-flavokawain B (**FB7**) (DMSO-d_6, 600/151 MHz)

Figure S54. Predicted Boiled-Egg plot from swissADME online web tool for 1-(2'-hydroxy-4',6'-dimethoxyphenyl)-3-(3''-*O*-β-D-(4'''-*O*-methylglucopiranosyl)-phenyl)-prop-2-en-1-on - 3'-*O*-β-D-(4″-*O*-methylglucopyranosyl)-flavokawain B (**FB7**)

Figure S55. 1-(2'-hydroxy-4',6'-dimethoxyphenyl)-3-(3''-*O*-β-D-(4'''-*O*-methylglucopiranosyl)-phenyl)-prop-2-en-1-on - 3'-*O*-β-D-(4″-*O*-methylglucopyranosyl)-flavokawain B (**FB7**) physicochemical and ADME parameters prediction using the SwissADME modelling

Figure S56. The UV absorption maxima of 1-(2'-hydroxy-4',6'-dimethoxyphenyl)-3-(3''-*O*-β-D-(4'''-*O*-methylglucopiranosyl)-4''-hydroxyphenyl)-prop-2-en-1-on - 3'-*O*-β-D-(4″-*O*-methylglucopyranosyl)-4''-hydroxyflavokawain B (**FB8**)

Figure S57. ^{1}H NMR spectra of 1-(2'-hydroxy-4',6'-dimethoxyphenyl)-3-(3''-*O*-β-D-(4'''-*O*-methylglucopiranosyl)-4''-hydroxyphenyl)-prop-2-en-1-on - 3'-*O*-β-D-(4″-*O*-methylglucopyranosyl)-4''-hydroxyflavokawain B (**FB8**) (DMSO-d_6, 600 MHz)

Figure S58. Flavone part of the ^{1}H NMR spectral 1-(2'-hydroxy-4',6'-dimethoxyphenyl)-3-(3''-*O*-β-D-(4'''-*O*-methylglucopiranosyl)-4''-hydroxyphenyl)-prop-2-en-1-on - 3'-*O*-β-D-(4″-*O*-methylglucopyranosyl)-4''-hydroxyflavokawain B (**FB8**) (DMSO-d_6, 600 MHz)

Figure S59. ^{13}C NMR spectra of 1-(2'-hydroxy-4',6'-dimethoxyphenyl)-3-(3''-*O*-β-D-(4'''-*O*-methylglucopiranosyl)-4''-hydroxyphenyl)-prop-2-en-1-on - 3'-*O*-β-D-(4″-*O*-methylglucopyranosyl)-4''-hydroxyflavokawain B (**FB8**) (DMSO-d_6, 151 MHz)

Figure S60. COSY spectrum of 1-(2'-hydroxy-4',6'-dimethoxyphenyl)-3-(3''-*O*-β-D-(4'''-*O*-methylglucopiranosyl)-4''-hydroxyphenyl)-prop-2-en-1-on - 3'-*O*-β-D-(4″-*O*-methylglucopyranosyl)-4''-hydroxyflavokawain B (**FB8**) (DMSO-d_6, 600 MHz)

Figure S61. HSQC spectrum of 1-(2'-hydroxy-4',6'-dimethoxyphenyl)-3-(3''-*O*-β-D-(4'''-*O*-methylglucopiranosyl)-4''-hydroxyphenyl)-prop-2-en-1-on - 3'-*O*-β-D-(4″-*O*-methylglucopyranosyl)-4''-hydroxyflavokawain B (**FB8**) (DMSO-d_6, 600/151 MHz)

Figure S62. HMBC spectrum of 1-(2'-hydroxy-4',6'-dimethoxyphenyl)-3-(3''-*O*-β-D-(4'''-*O*-methylglucopiranosyl)-4''-hydroxyphenyl)-prop-2-en-1-on - 3'-*O*-β-D-(4″-*O*-methylglucopyranosyl)-4''-hydroxyflavokawain B (**FB8**) (DMSO-d_6, 600/151 MHz)

Figure S63. Predicted Boiled-Egg plot from swissADME online web tool for 1-(2'-hydroxy-4',6'-dimethoxyphenyl)-3-(3''-*O*-β-D-(4'''-*O*-methylglucopiranosyl)-4''-hydroxyphenyl)-prop-2-en-1-on - 3'-*O*-β-D-(4″-*O*-methylglucopyranosyl)-4''-hydroxyflavokawain B (**FB8**)

Figure S64. 1-(2'-hydroxy-4',6'-dimethoxyphenyl)-3-(3''-*O*-β-D-(4'''-*O*-methylglucopiranosyl)-4''-hydroxyphenyl)-prop-2-en-1-on - 3'-*O*-β-D-(4″-*O*-methylglucopyranosyl)-4''-hydroxyflavokawain B (**FB8**) physicochemical and ADME parameters prediction using the SwissADME modelling

Figure S1. The UV absorption maxima of 1-(2'-hydroxy-4',6'-dimethoxyphenyl)-3-phenyl-prop-2-en-1-on - flavokawain B (**FB1**)

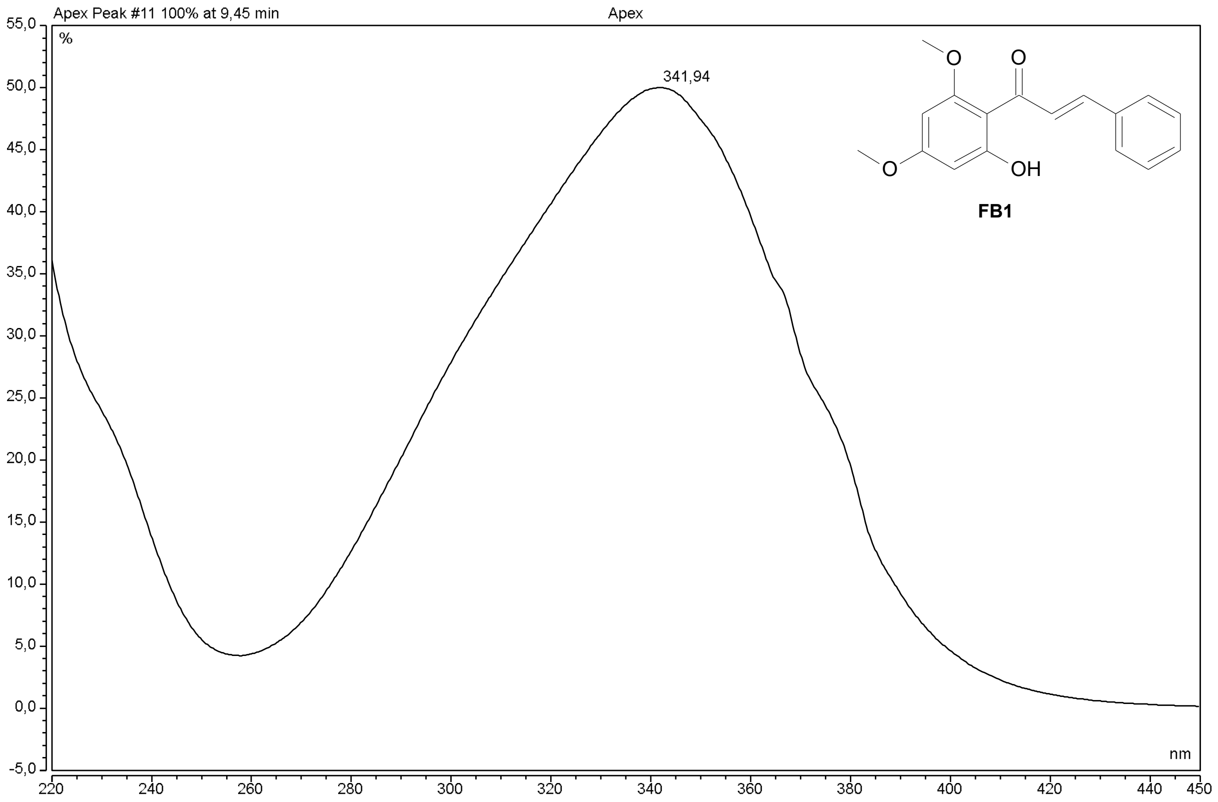

Figure S2. ^{1}H NMR spectra of 1-(2'-hydroxy-4',6'-dimethoxyphenyl)-3-phenyl-prop-2-en-1-on - flavokawain B (**FB1**) (DMSO-d_6, 600 MHz)

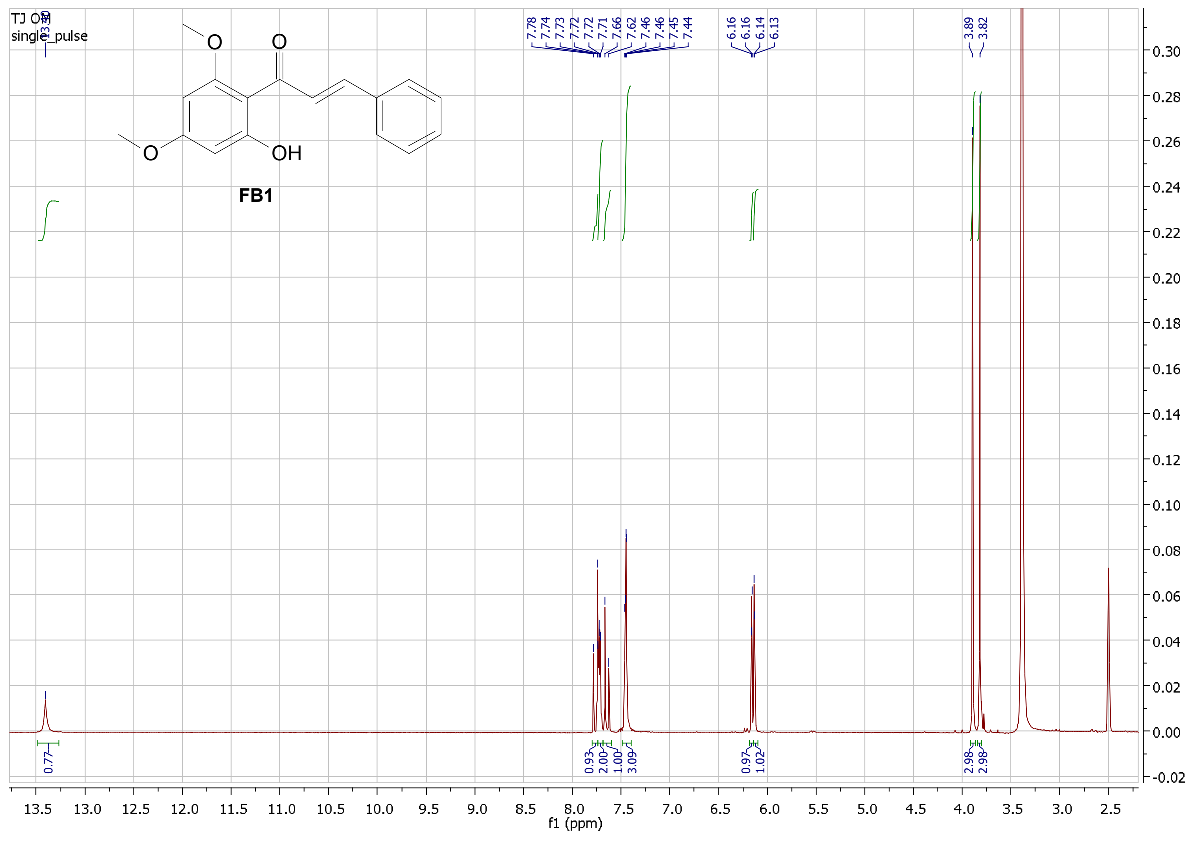

Figure S3. Flavone part of the ^{1}H NMR spectral 1-(2'-hydroxy-4',6'-dimethoxyphenyl)-3-phenyl-prop-2-en-1-on - flavokawain B (**FB1**) (DMSO-d_6, 600 MHz)

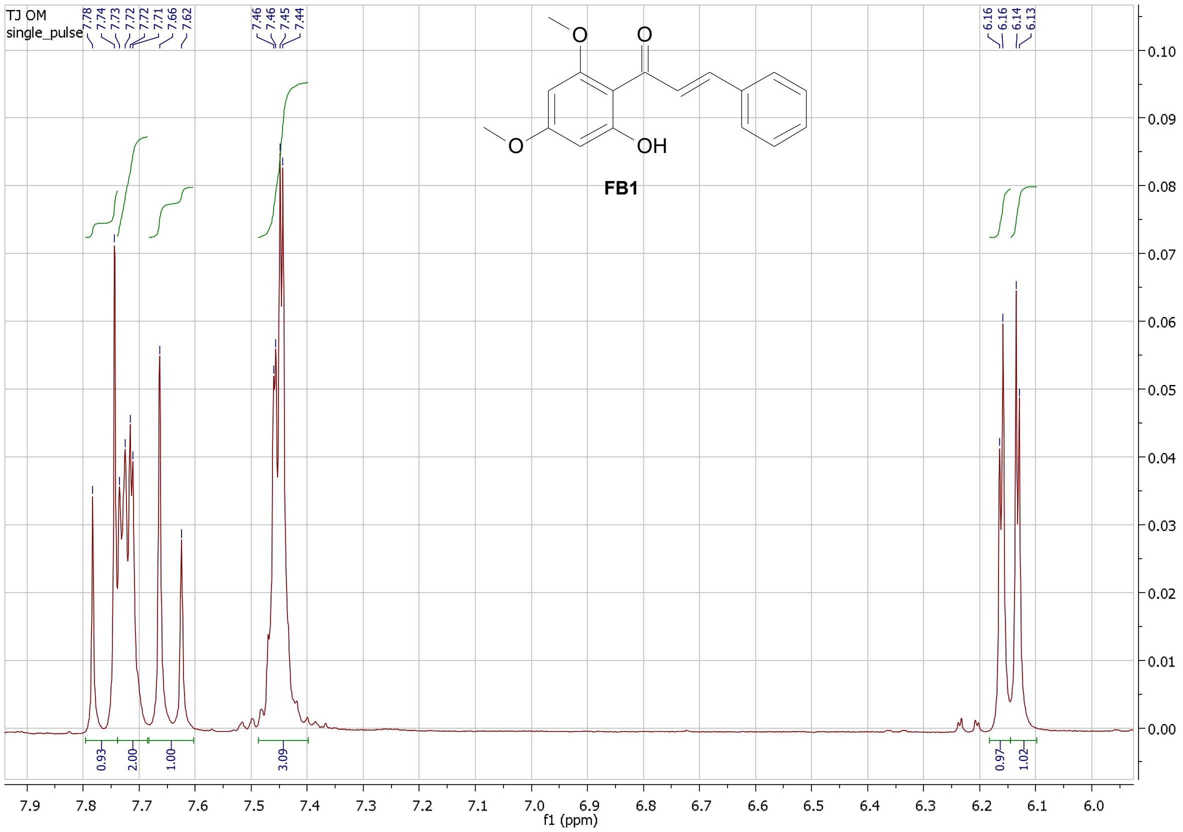

Figure S4. ^{13}C NMR spectra of 1-(2'-hydroxy-4',6'-dimethoxyphenyl)-3-phenyl-prop-2-en-1-on - flavokawain B (**FB1**) (DMSO-d_6, 151 MHz)

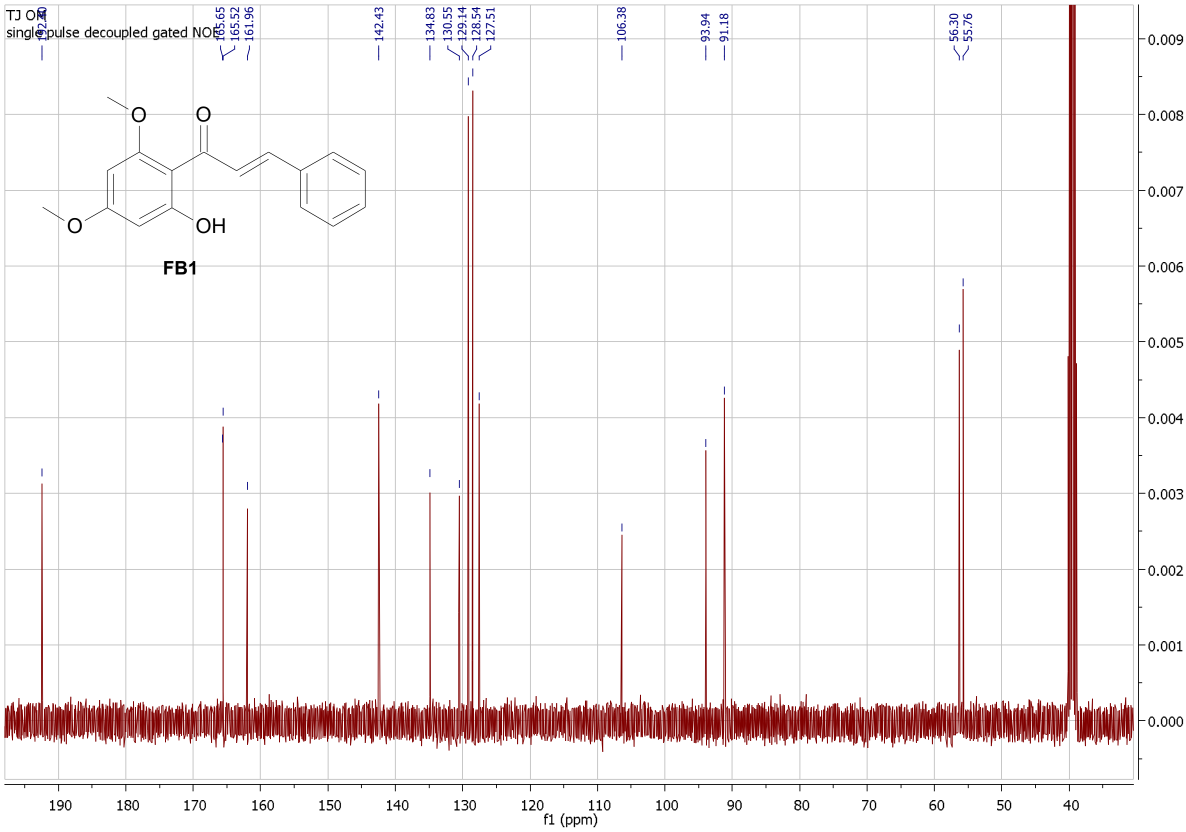

Figure S5. COSY spectrum of 1-(2'-hydroxy-4',6'-dimethoxyphenyl)-3-phenyl-prop-2-en-1-on - flavokawain B (**FB1**) (DMSO-d_6, 600 MHz)

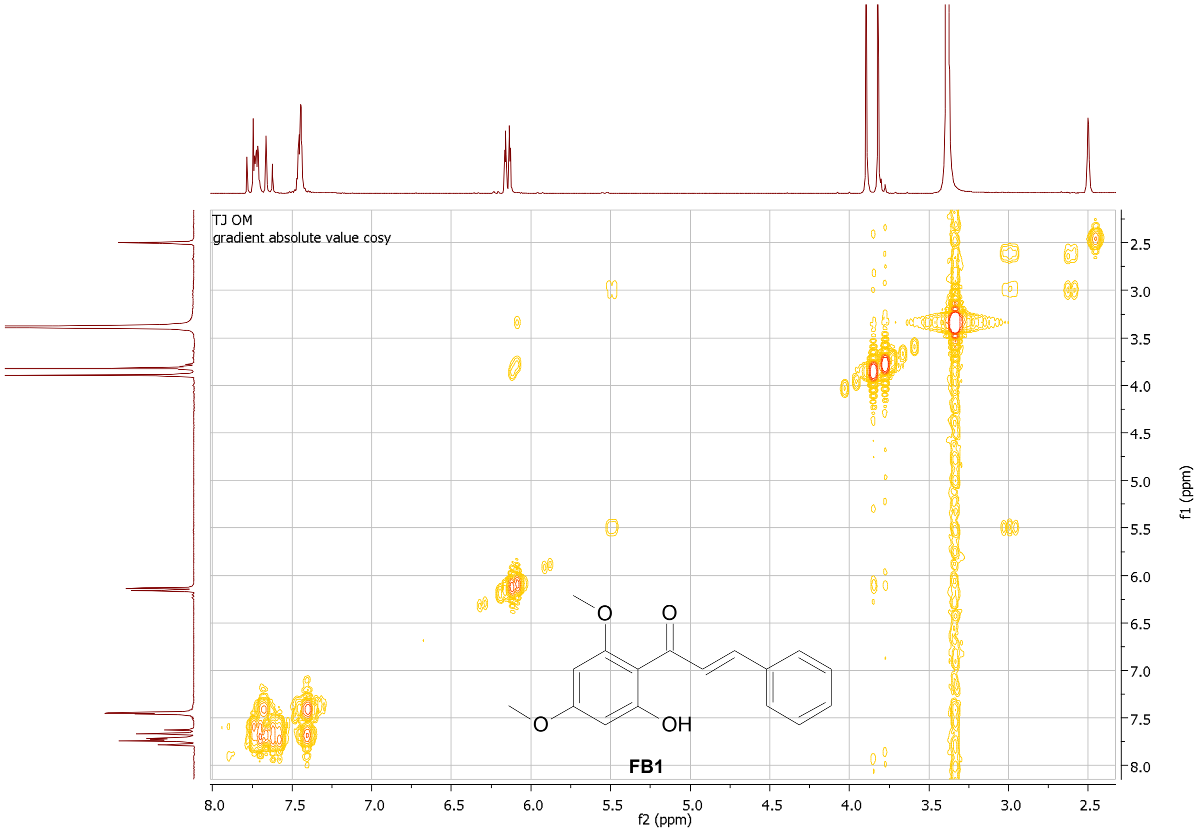

Figure S6. HSQC spectrum of 1-(2'-hydroxy-4',6'-dimethoxyphenyl)-3-phenyl-prop-2-en-1-on - flavokawain B (**FB1**) (DMSO-d_6, 600/151 MHz)

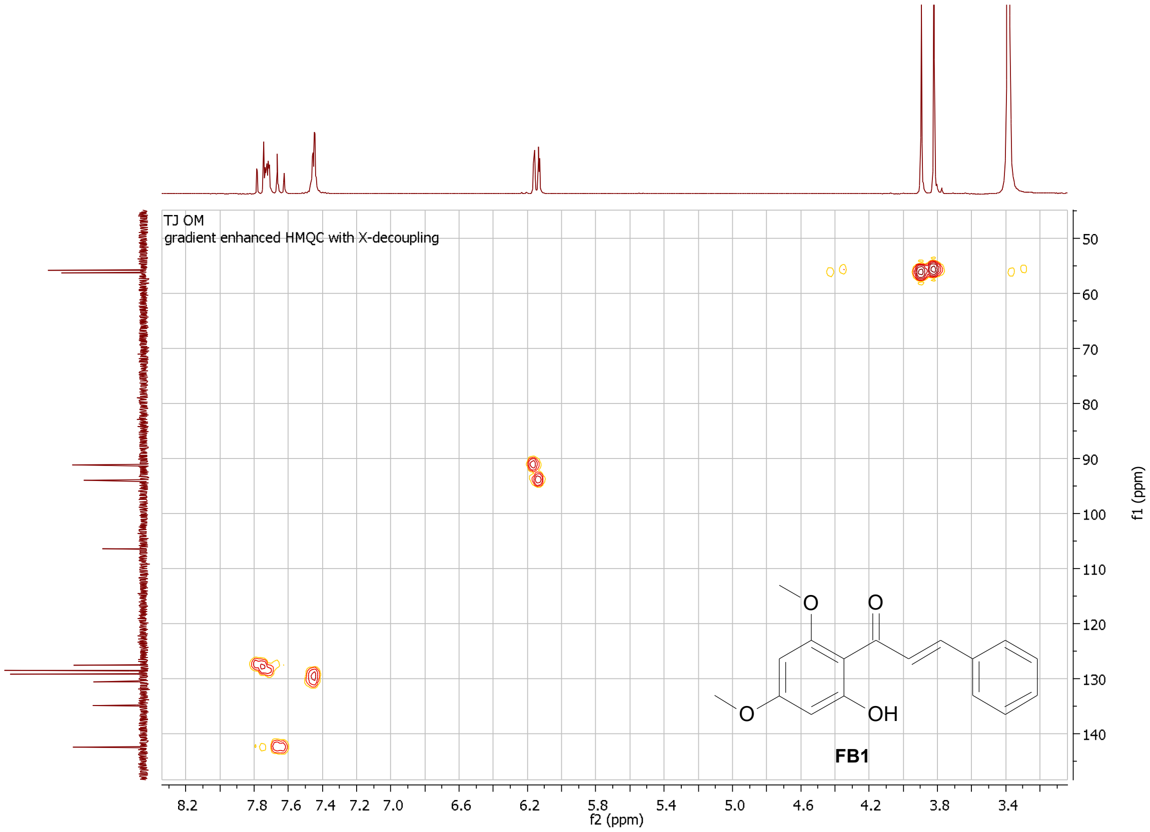

Figure S7. HMBC spectrum of 1-(2'-hydroxy-4',6'-dimethoxyphenyl)-3-phenyl-prop-2-en-1-on - flavokawain B (**FB1**) (DMSO-d_6, 600/151 MHz)

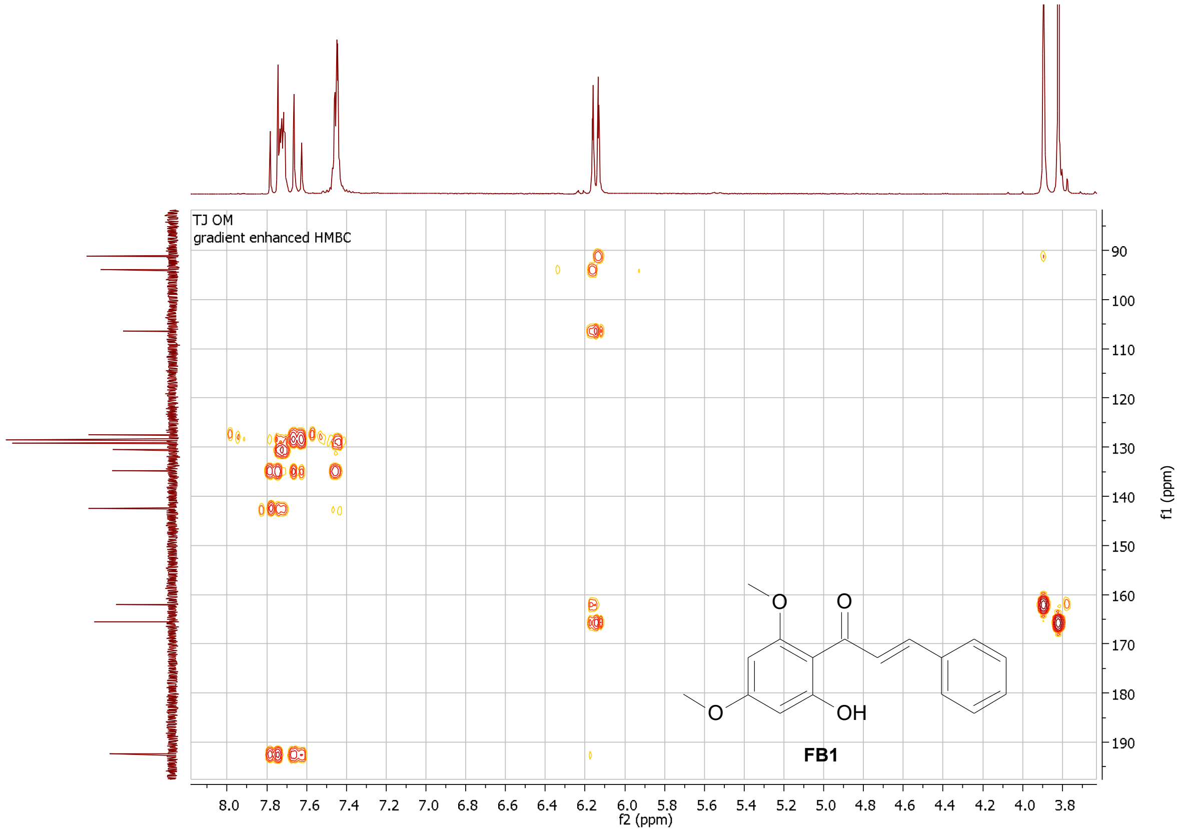

Figure S8. Predicted Boiled-Egg plot from swissADME online web tool for 1-(2'-hydroxy-4',6'-dimethoxyphenyl)-3-phenyl-prop-2-en-1-on - flavokawain B (**FB1**)

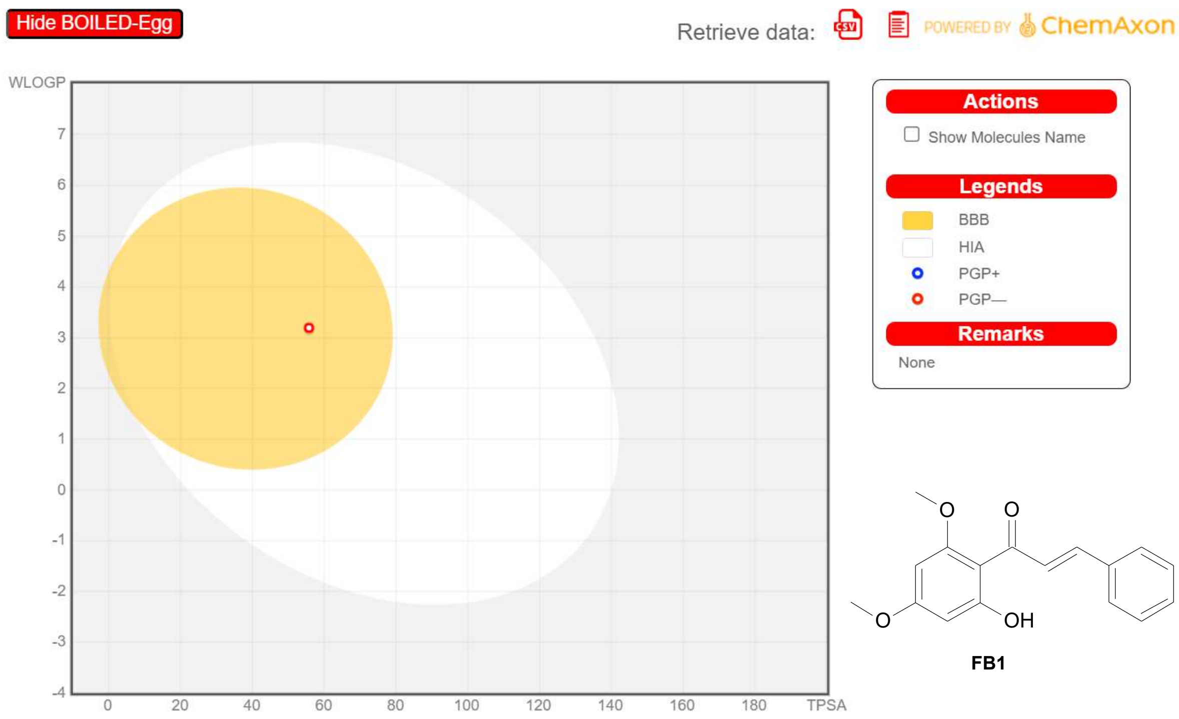

Figure S9. 1-(2'-hydroxy-4',6'-dimethoxyphenyl)-3-phenyl-prop-2-en-1-on - flavokawain B (**FB1**) physicochemical and ADME parameters prediction using the SwissADME modelling

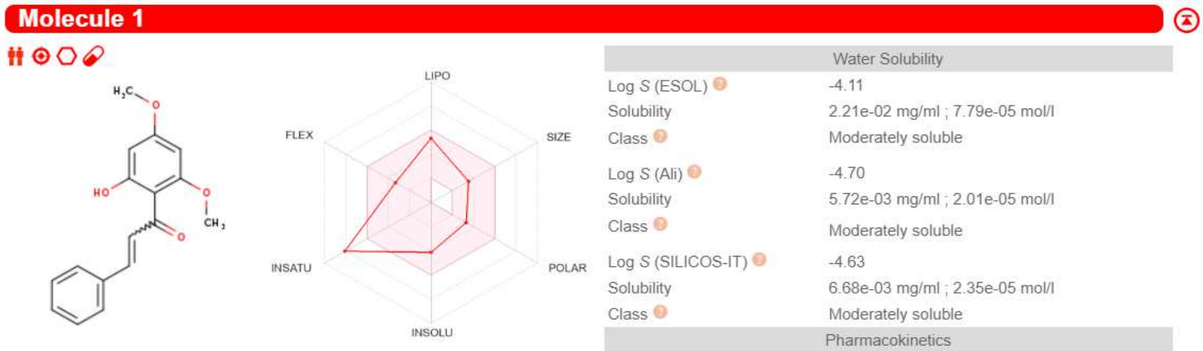

SMILES COc1cc(O)c(c(c1)OC)C(=O)/C=C/c1ccccc1

Physicochemical Properties	
Formula	C17H16O4
Molecular weight	284.31 g/mol
Num. heavy atoms	21
Num. arom. heavy atoms	12
Fraction Csp3	0.12
Num. rotatable bonds	5
Num. H-bond acceptors	4
Num. H-bond donors	1
Molar Refractivity	81.26
TPSA	55.76 Å²
**Lipophilicity**	
Log $P_{o/w}$ (iLOGP)	2.63
Log $P_{o/w}$ (XLOGP3)	3.83
Log $P_{o/w}$ (WLOGP)	3.20
Log $P_{o/w}$ (MLOGP)	2.08
Log $P_{o/w}$ (SILICOS-IT)	3.54
Consensus Log $P_{o/w}$	3.06

Water Solubility	
Log *S* (ESOL)	-4.11
Solubility	2.21e-02 mg/ml ; 7.79e-05 mol/l
Class	Moderately soluble
Log *S* (Ali)	-4.70
Solubility	5.72e-03 mg/ml ; 2.01e-05 mol/l
Class	Moderately soluble
Log *S* (SILICOS-IT)	-4.63
Solubility	6.68e-03 mg/ml ; 2.35e-05 mol/l
Class	Moderately soluble
**Pharmacokinetics**	
GI absorption	High
BBB permeant	Yes
P-gp substrate	No
CYP1A2 inhibitor	Yes
CYP2C19 inhibitor	Yes
CYP2C9 inhibitor	Yes
CYP2D6 inhibitor	No
CYP3A4 inhibitor	Yes
Log K_p (skin permeation)	-5.31 cm/s
**Druglikeness**	
Lipinski	Yes; 0 violation
Ghose	Yes
Veber	Yes
Egan	Yes
Muegge	Yes
Bioavailability Score	0.55
**Medicinal Chemistry**	
PAINS	0 alert
Brenk	1 alert: michael_acceptor_1
Leadlikeness	No; 1 violation: XLOGP3>3.5
Synthetic accessibility	2.73

Figure S10. The UV absorption maxima of 1-(2',4'-dihydroxy-6'-methoxyphenyl)-3-phenyl-prop-2-en-1-on - cardamonin (**FB2**)

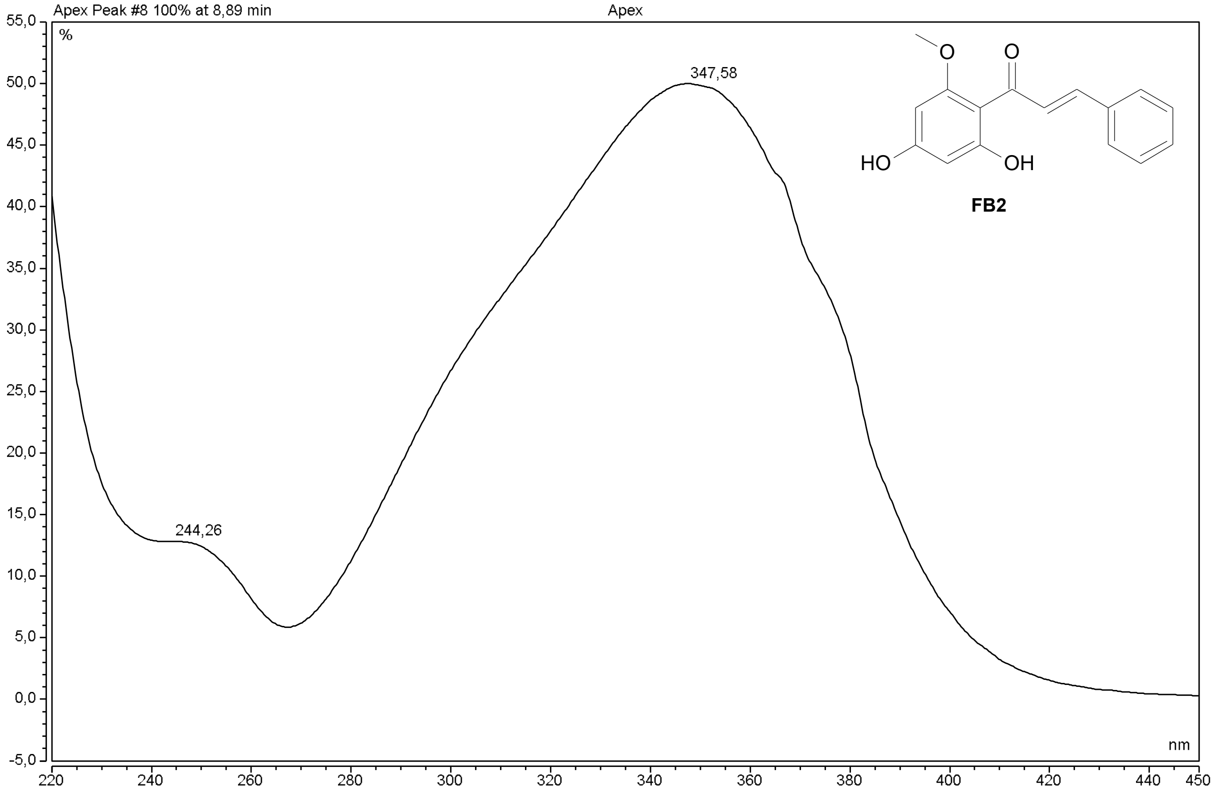

Figure S11. ^{1}H NMR spectra of 1-(2',4'-dihydroxy-6'-methoxyphenyl)-3-phenyl-prop-2-en-1-on - cardamonin (**FB2**) (DMSO-d_6, 600 MHz)

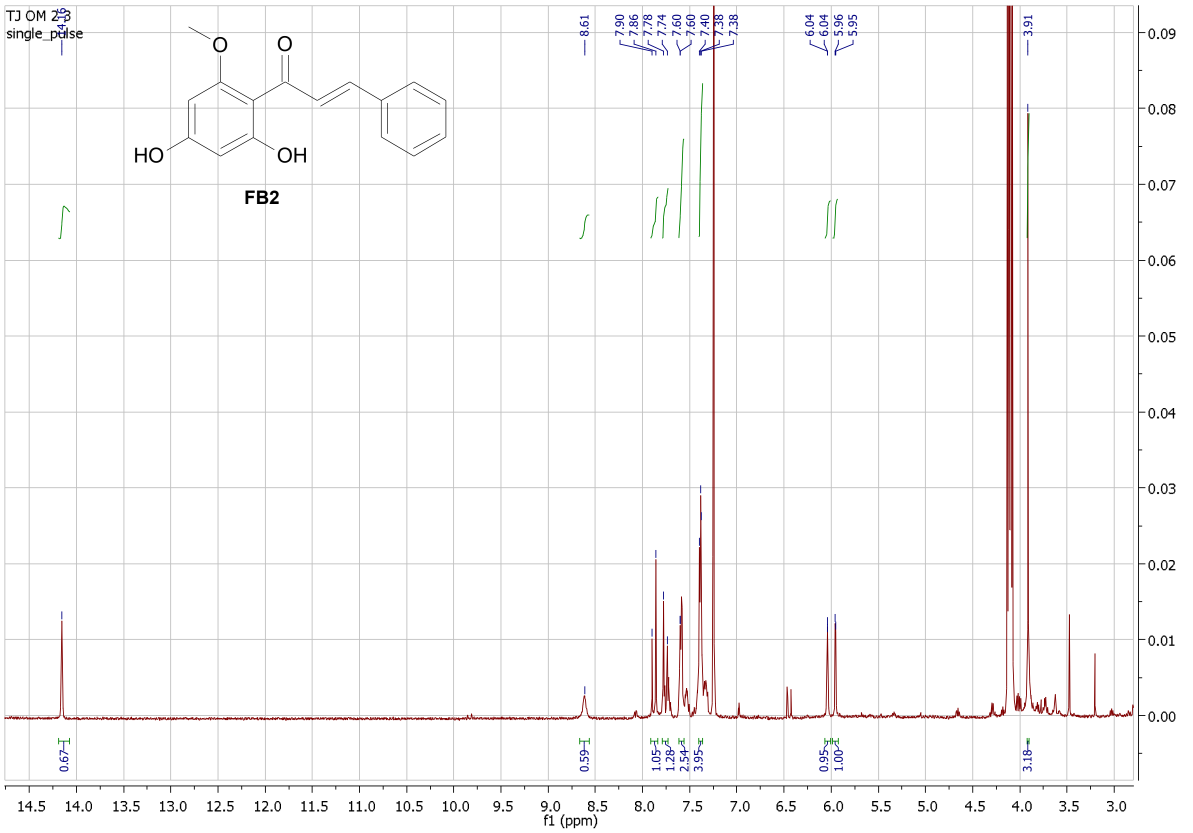

Figure S12. Flavone part of the ^{1}H NMR spectral 1-(2',4'-dihydroxy-6'-methoxyphenyl)-3-phenyl-prop-2-en-1-on - cardamonin (**FB2**) (DMSO-d_6, 600 MHz)

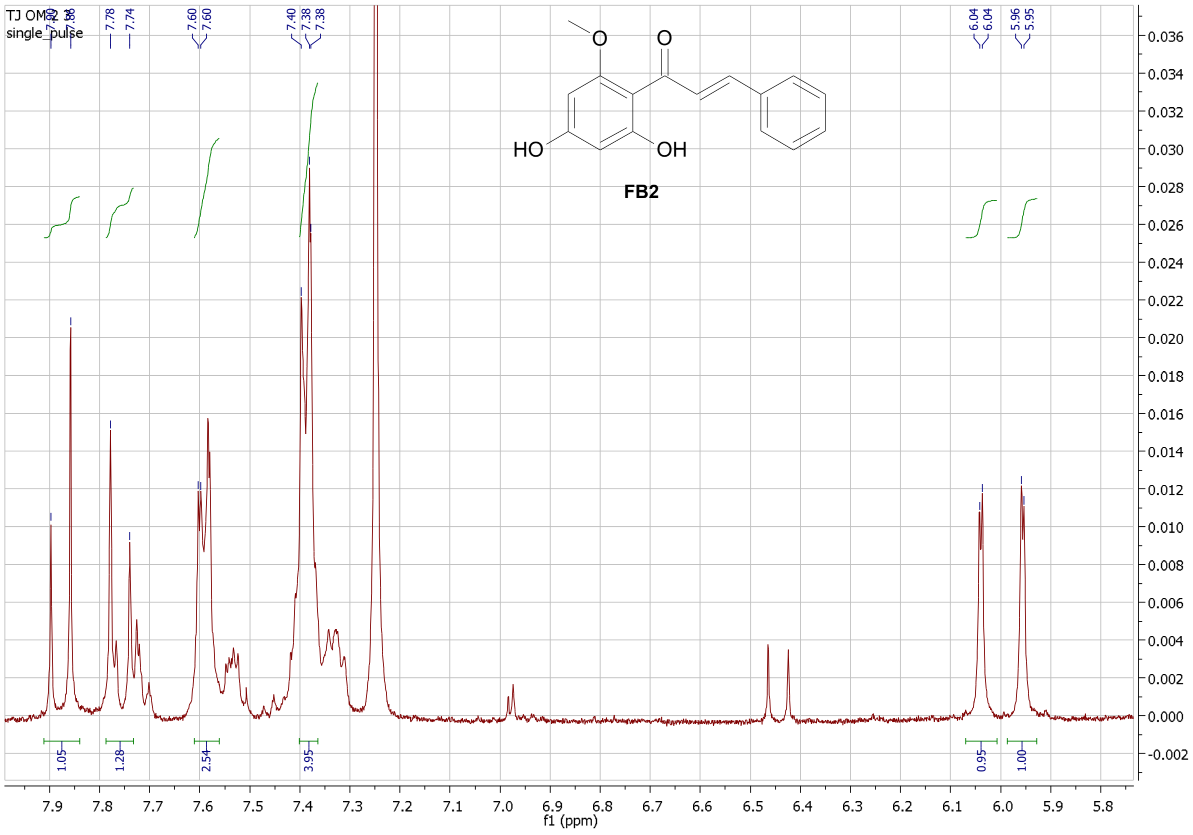

Figure S12. Predicted Boiled-Egg plot from swissADME online web tool for 1-(2',4'-dihydroxy-6'-methoxyphenyl)-3-phenyl-prop-2-en-1-on - cardamonin (**FB2**)

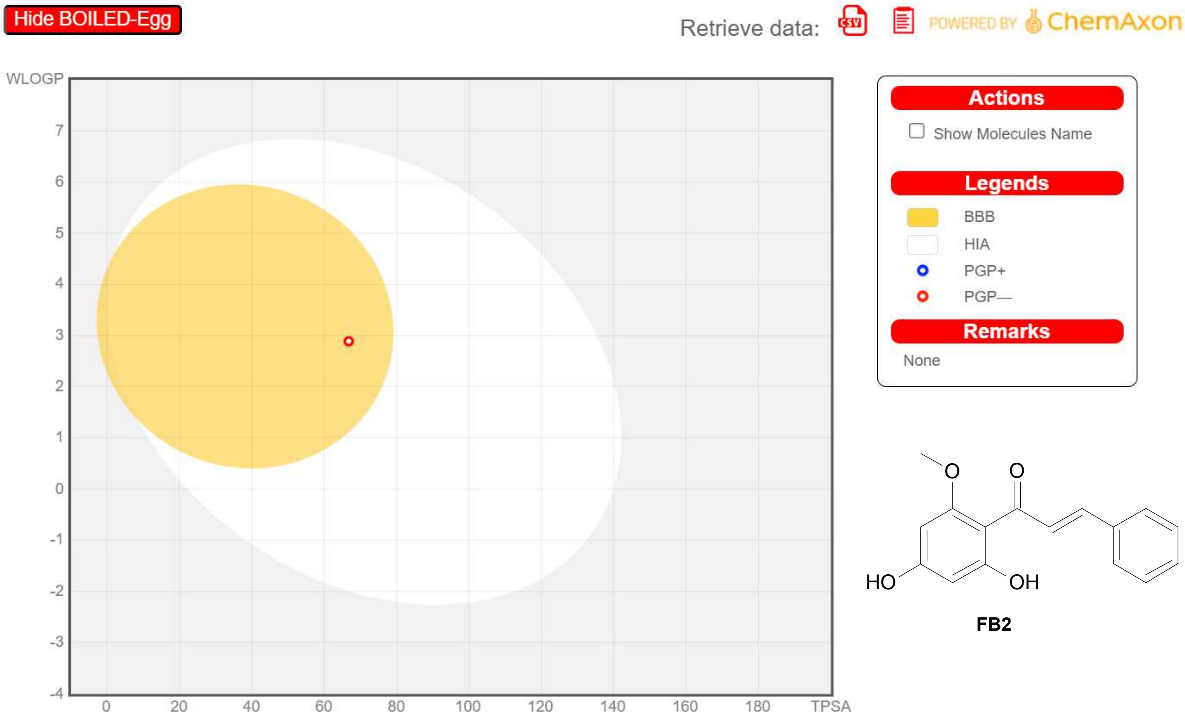

Figure S14. 1-(2',4'-dihydroxy-6'-methoxyphenyl)-3-phenyl-prop-2-en-1-on - cardamonin (**FB2**) physicochemical and ADME parameters prediction using the SwissADME modelling

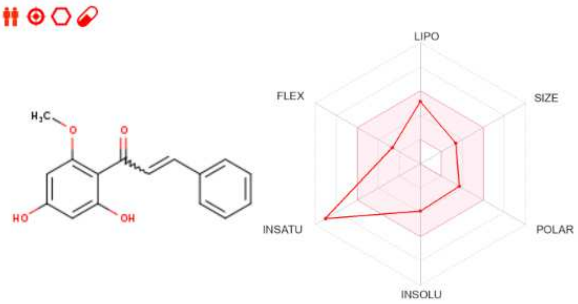

SMILES COc1cc(O)cc(c1C(=O)/C=C/c1ccccc1)O

Physicochemical Properties	
Formula	C16H14O4
Molecular weight	270.28 g/mol
Num. heavy atoms	20
Num. arom. heavy atoms	12
Fraction Csp3	0.06
Num. rotatable bonds	4
Num. H-bond acceptors	4
Num. H-bond donors	2
Molar Refractivity	76.79
TPSA	66.76 Å²

Lipophilicity	
Log $P_{o/w}$ (iLOGP)	1.78
Log $P_{o/w}$ (XLOGP3)	3.50
Log $P_{o/w}$ (WLOGP)	2.89
Log $P_{o/w}$ (MLOGP)	1.83
Log $P_{o/w}$ (SILICOS-IT)	3.00
Consensus Log $P_{o/w}$	2.60

Water Solubility	
Log *S* (ESOL)	-3.90
Solubility	3.40e-02 mg/ml ; 1.26e-04 mol/l
Class	Soluble
Log *S* (Ali)	-4.59
Solubility	7.03e-03 mg/ml ; 2.60e-05 mol/l
Class	Moderately soluble
Log *S* (SILICOS-IT)	-3.93
Solubility	3.17e-02 mg/ml ; 1.17e-04 mol/l
Class	Soluble

Pharmacokinetics	
GI absorption	High
BBB permeant	Yes
P-gp substrate	No
CYP1A2 inhibitor	Yes
CYP2C19 inhibitor	No
CYP2C9 inhibitor	Yes
CYP2D6 inhibitor	No
CYP3A4 inhibitor	Yes
Log K_p (skin permeation)	-5.46 cm/s

Druglikeness	
Lipinski	Yes; 0 violation
Ghose	Yes
Veber	Yes
Egan	Yes
Muegge	Yes
Bioavailability Score	0.55

Medicinal Chemistry	
PAINS	0 alert
Brenk	1 alert: michael_acceptor_1
Leadlikeness	Yes
Synthetic accessibility	2.62

Figure S15. The UV absorption maxima of 1-(4'-*O*-β-D-(4'''-*O*-methylglucopiranosyl)-2'-hydroxy-6'-methoxyphenyl)-3-phenyl-prop-2-en-1-on - 4'-*O*-β-D-(4''-*O*-methylglucopyranosyl)-cardamonin (**FB3**)

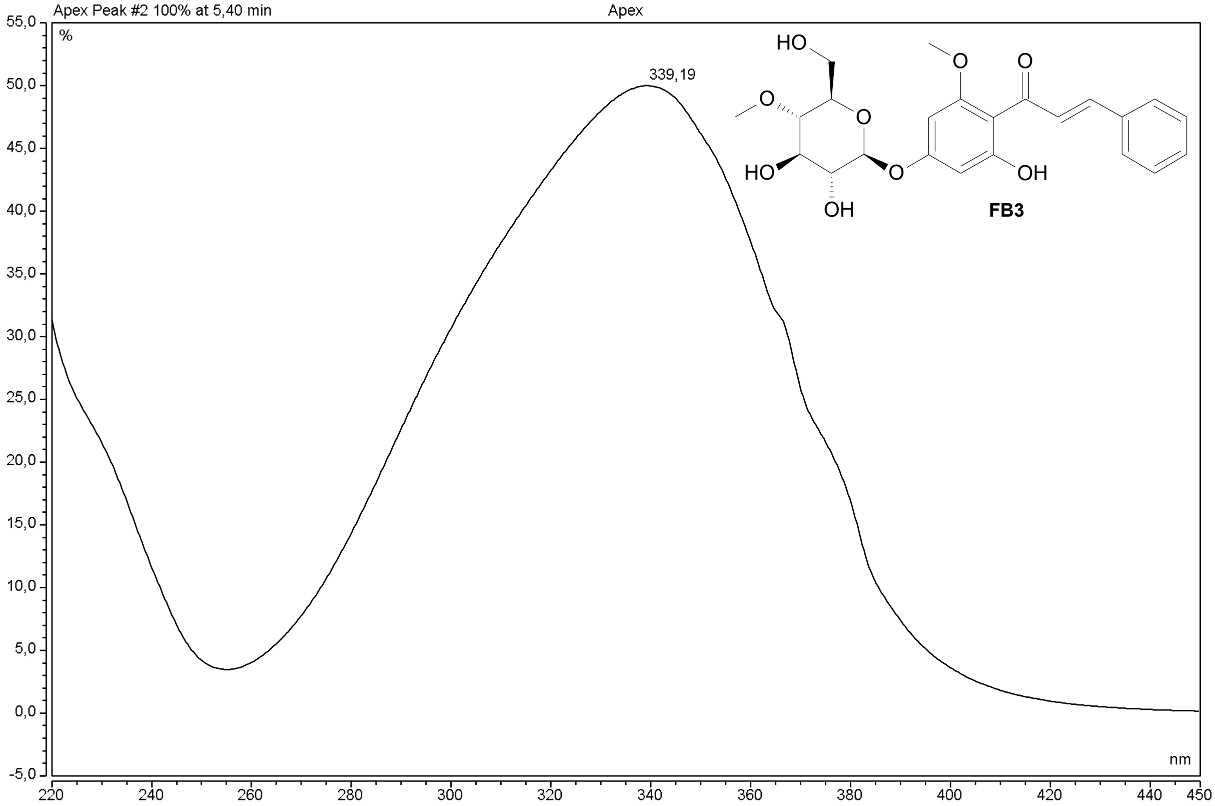

Figure S16. ^{1}H NMR spectra of 1-(4'-*O*-β-D-(4'''-*O*-methylglucopiranosyl)-2'-hydroxy-6'-methoxyphenyl)-3-phenyl-prop-2-en-1-on - 4'-*O*-β-D-(4''-*O*-methylglucopyranosyl)-cardamonin (**FB3**) (DMSO-d_6, 600 MHz)

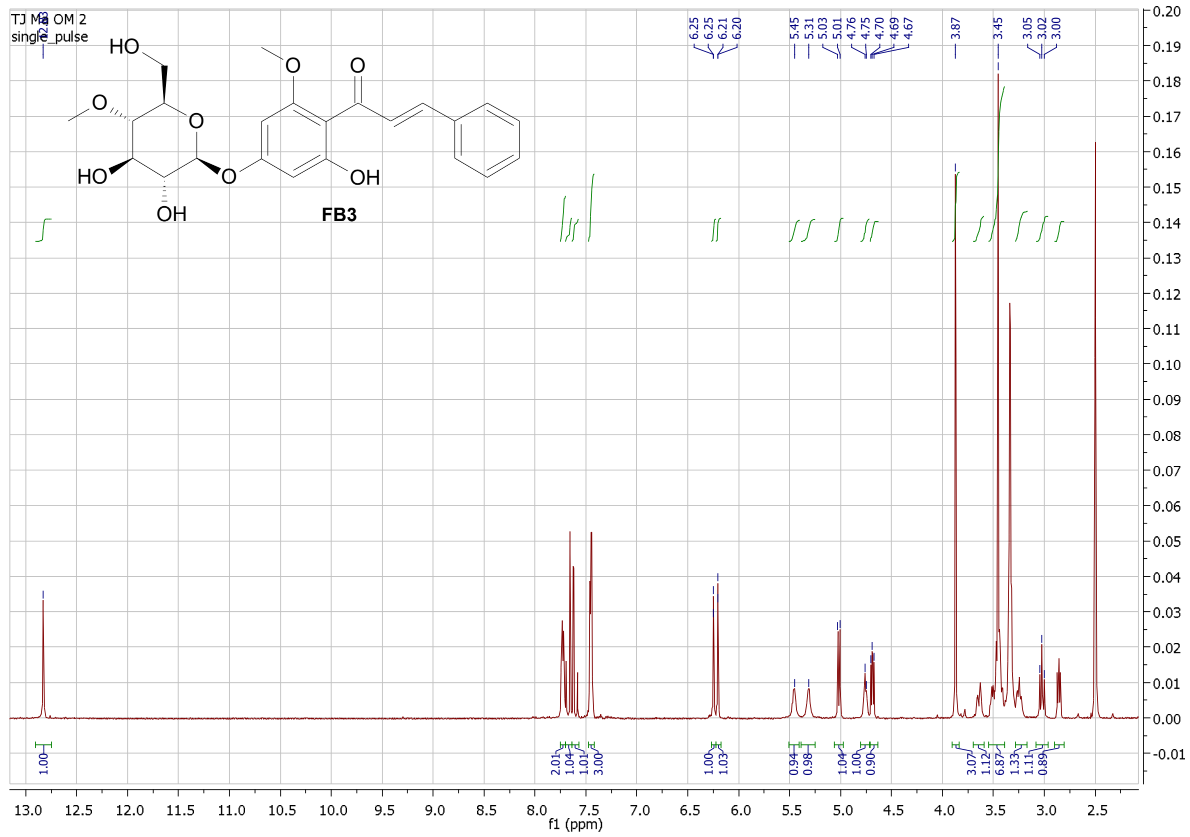

Figure S17. Flavone part of the ^{1}H NMR spectral 1-(4'-*O*-β-D-(4'''-*O*-methylglucopiranosyl)-2'-hydroxy-6'-methoxyphenyl)-3-phenyl-prop-2-en-1-on - 4'-*O*-β-D-(4''-*O*-methylglucopyranosyl)-cardamonin (**FB3**) (DMSO-d_6, 600 MHz)

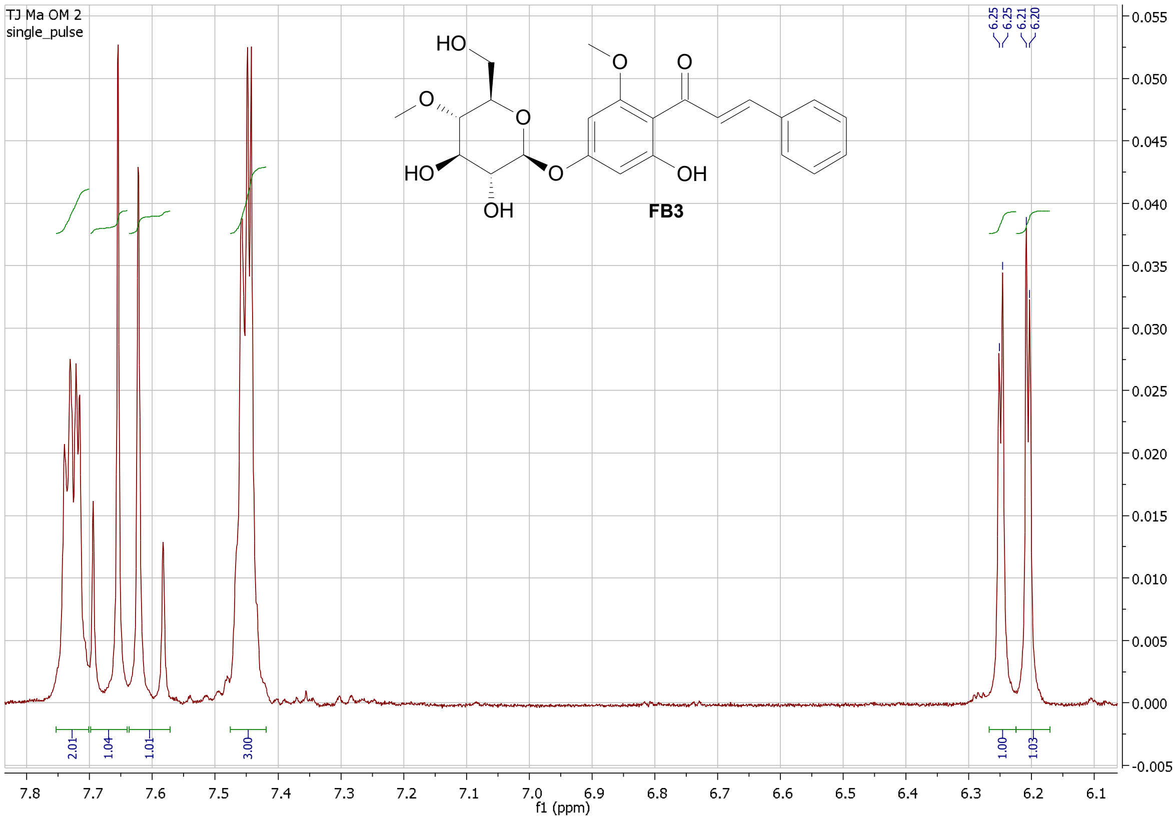

Figure S18. ^{13}C NMR spectra of 1-(4'-*O*-β-D-(4'''-*O*-methylglucopiranosyl)-2'-hydroxy-6'-methoxyphenyl)-3-phenyl-prop-2-en-1-on - 4'-*O*-β-D-(4''-*O*-methylglucopyranosyl)-cardamonin (**FB3**) (DMSO-d_6, 151 MHz)

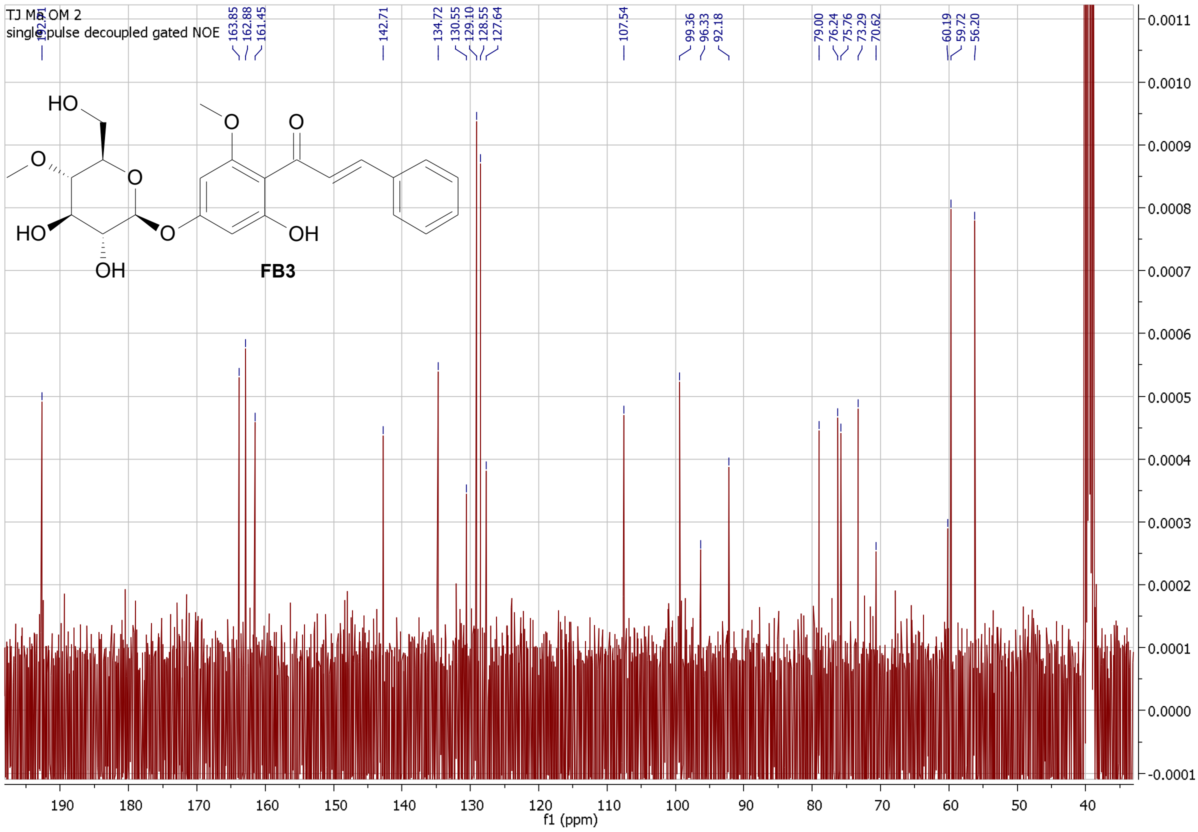

Figure S19. COSY spectrum of 1-(4'-*O*-β-D-(4'''-*O*-methylglucopiranosyl)-2'-hydroxy-6'-methoxyphenyl)-3-phenyl-prop-2-en-1-on - 4'-*O*-β-D-(4''-*O*-methylglucopyranosyl)-cardamonin (**FB3**) (DMSO-d_6, 600 MHz)

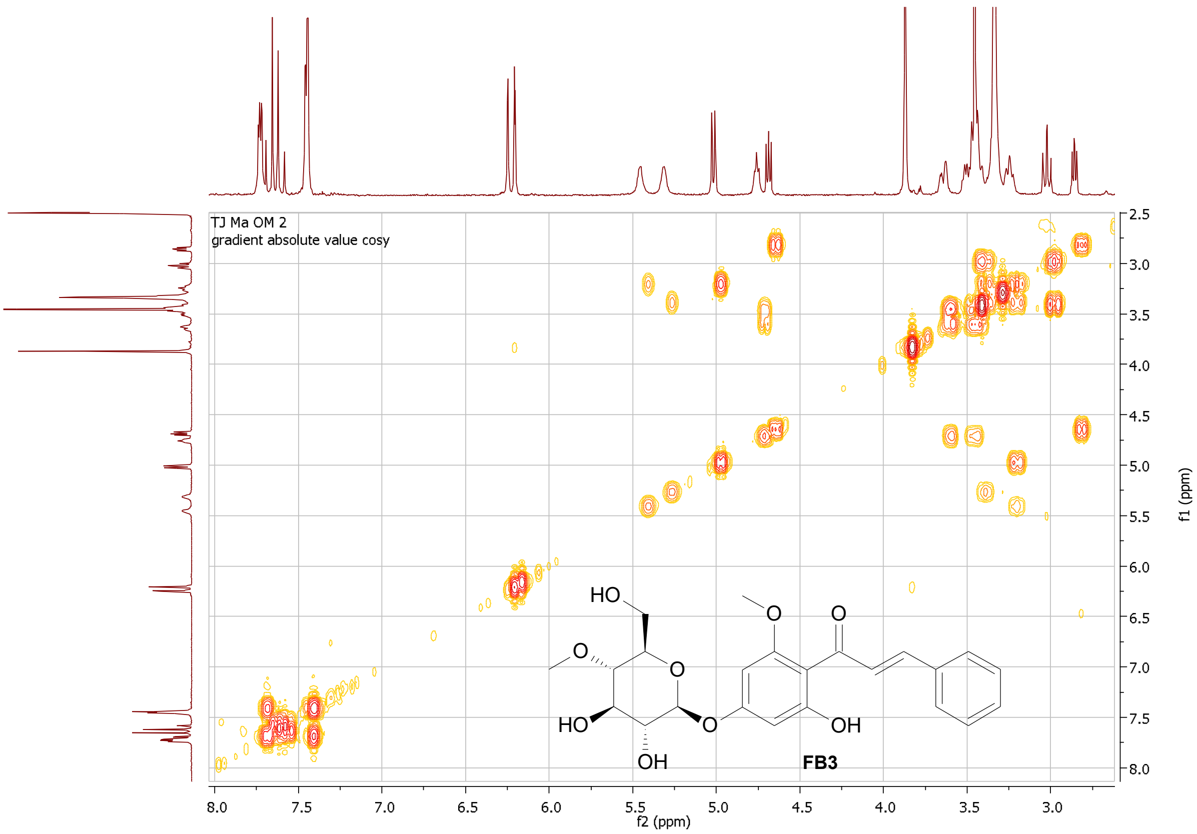

Figure S20. HSQC spectrum of 1-(4'-*O*-β-D-(4'''-*O*-methylglucopiranosyl)-2'-hydroxy-6'-methoxyphenyl)-3-phenyl-prop-2-en-1-on - 4'-*O*-β-D-(4''-*O*-methylglucopyranosyl)-cardamonin (**FB3**) (DMSO-d_6, 600/151 MHz)

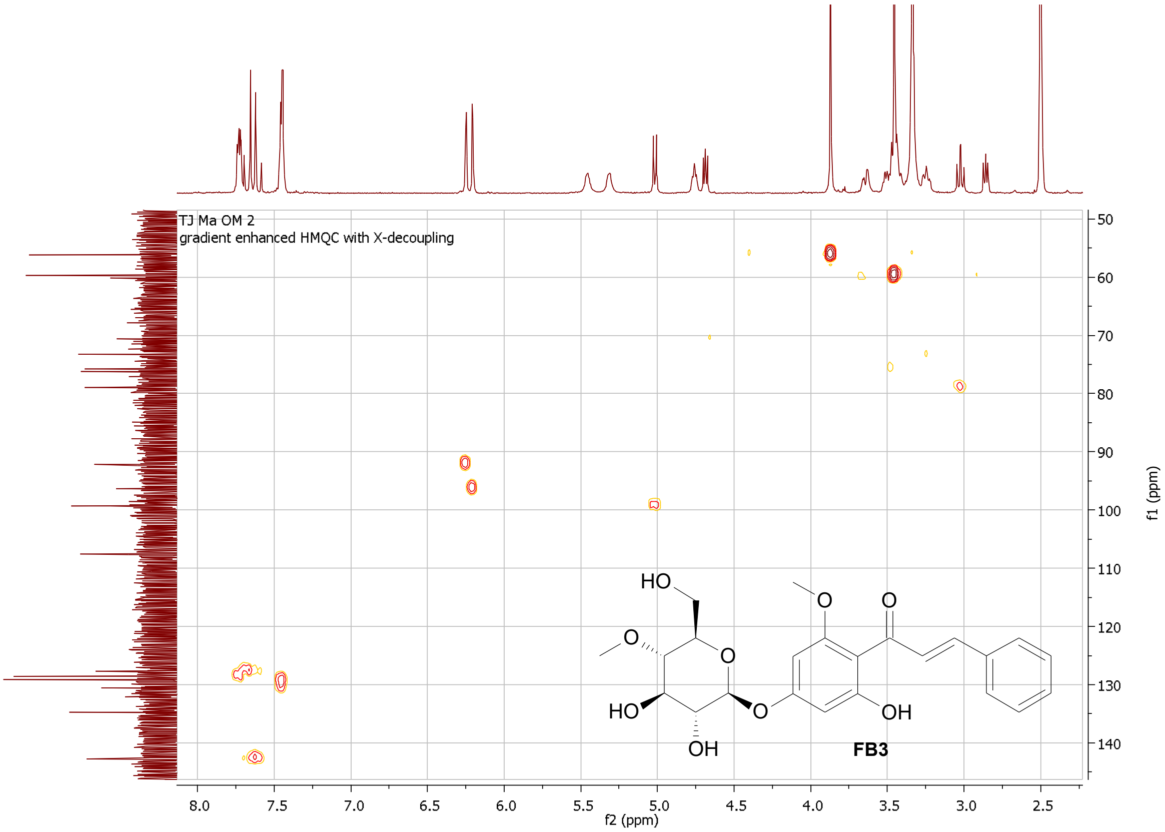

Figure S21. HMBC spectrum of 1-(4’-*O*-β-D-(4’’’-*O*-methylglucopiranosyl)-2’-hydroxy-6’-methoxyphenyl)-3-phenyl-prop-2-en-1-on - 4’-*O*-β-D-(4”-*O*-methylglucopyranosyl)-cardamonin (**FB3**) (DMSO-d_6, 600/151 MHz)

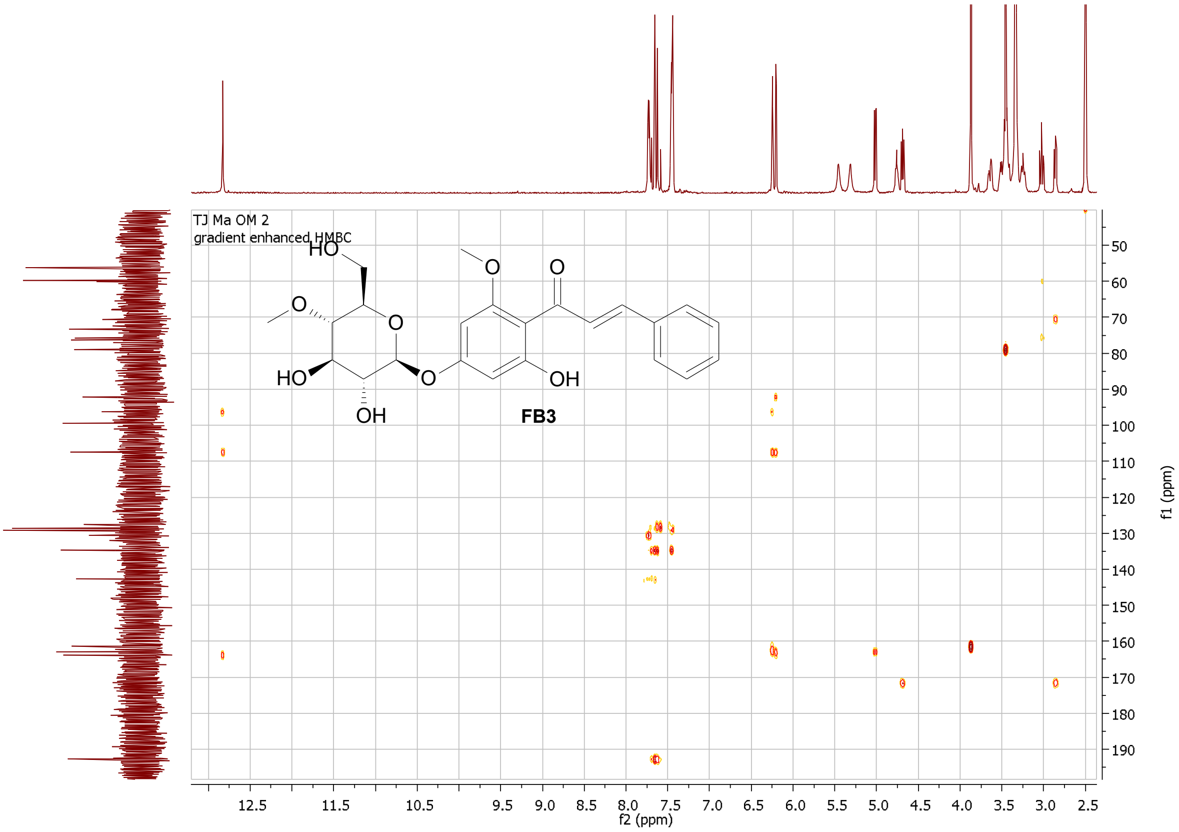

Figure S22. Predicted Boiled-Egg plot from swissADME online web tool for 1-(4’-*O*-β-D-(4’’’-*O*-methylglucopiranosyl)-2’-hydroxy-6’-methoxyphenyl)-3-phenyl-prop-2-en-1-on - 4’-*O*-β-D-(4”-*O*-methylglucopyranosyl)-cardamonin (**FB3**)

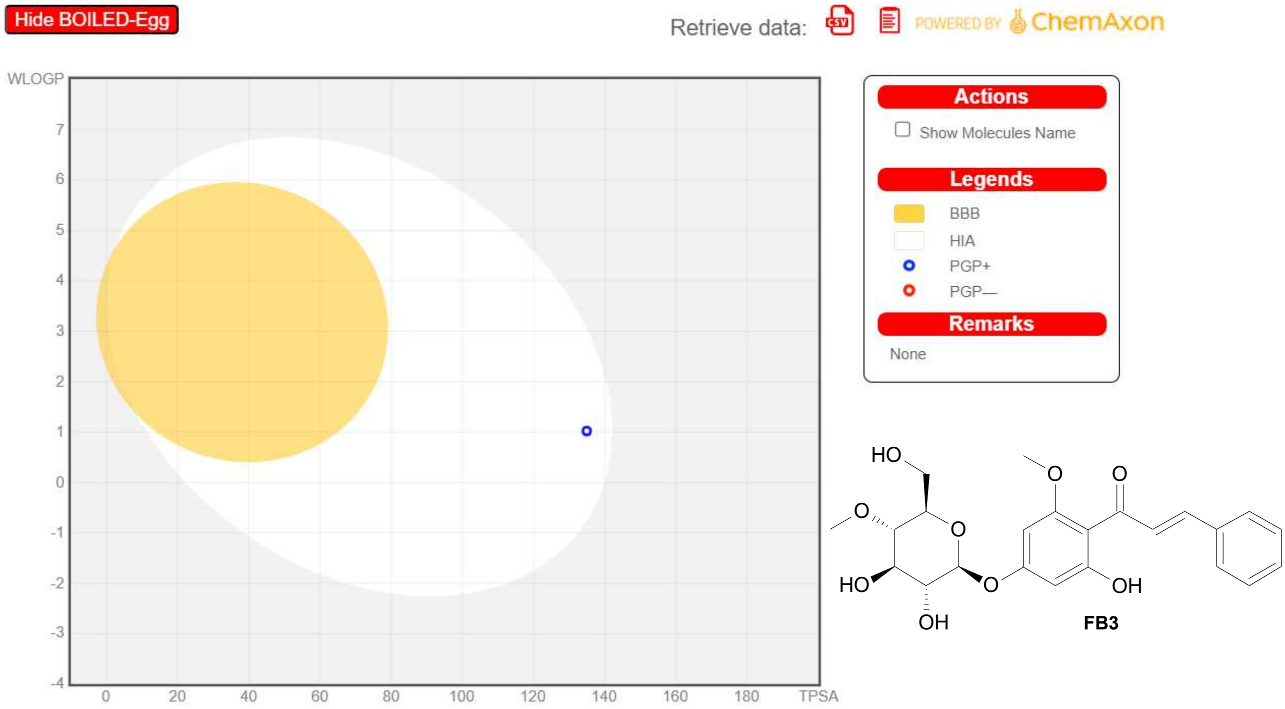

Figure S23. 1-(4’-*O*-β-D-(4’’’-*O*-methylglucopiranosyl)-2’-hydroxy-6’-methoxyphenyl)-3-phenyl-prop-2-en-1-on - 4’-*O*-β-D-(4”-*O*-methylglucopyranosyl)-cardamonin (**FB3**) physicochemical and ADME parameters prediction using the SwissADME modelling

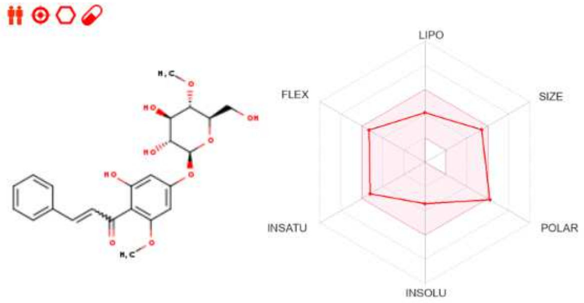

SMILES CO[C@@H]1[C@@H](CO)O[C@H]([C@@H]([C@H]1O)O)Oc1cc(O)c(c(c1)OC)C(=O)/C=C/c1ccccc1

Physicochemical Properties	
Formula	C23H26O9
Molecular weight	446.45 g/mol
Num. heavy atoms	32
Num. arom. heavy atoms	12
Fraction Csp3	0.35
Num. rotatable bonds	8
Num. H-bond acceptors	9
Num. H-bond donors	4
Molar Refractivity	113.64
TPSA	134.91 Å²

Lipophilicity	
Log $P_{o/w}$ (iLOGP)	3.06
Log $P_{o/w}$ (XLOGP3)	1.68
Log $P_{o/w}$ (WLOGP)	1.02
Log $P_{o/w}$ (MLOGP)	-0.56
Log $P_{o/w}$ (SILICOS-IT)	1.52
Consensus Log $P_{o/w}$	1.34

Water Solubility	
Log *S* (ESOL)	-3.42
Solubility	1.71e-01 mg/ml ; 3.84e-04 mol/l
Class	Soluble
Log *S* (Ali)	-4.13
Solubility	3.33e-02 mg/ml ; 7.45e-05 mol/l
Class	Moderately soluble
Log *S* (SILICOS-IT)	-2.91
Solubility	5.51e-01 mg/ml ; 1.23e-03 mol/l
Class	Soluble

Pharmacokinetics	
GI absorption	High
BBB permeant	No
P-gp substrate	Yes
CYP1A2 inhibitor	No
CYP2C19 inhibitor	No
CYP2C9 inhibitor	No
CYP2D6 inhibitor	No
CYP3A4 inhibitor	No
Log K_p (skin permeation)	-7.83 cm/s

Druglikeness	
Lipinski	Yes; 0 violation
Ghose	Yes
Veber	Yes
Egan	No; 1 violation: TPSA>131.6
Muegge	Yes
Bioavailability Score	0.55

Medicinal Chemistry	
PAINS	0 alert
Brenk	1 alert: michael_acceptor_1
Leadlikeness	No; 2 violations: MW>350, Rotors>7
Synthetic accessibility	5.18

Figure S24. The UV absorption maxima of 1-(4’-*O*-β-D-(4’’’-*O*-methylglucopiranosyl)-2’-hydroxy-6’-methoxyphenyl)-3-(3’’-hydoxyphenyl)-prop-2-en-1-on - 4’-*O*-β-D-(4″-*O*-methylglucopyranosyl)-3”-hydroxycardamonin (**FB4**)

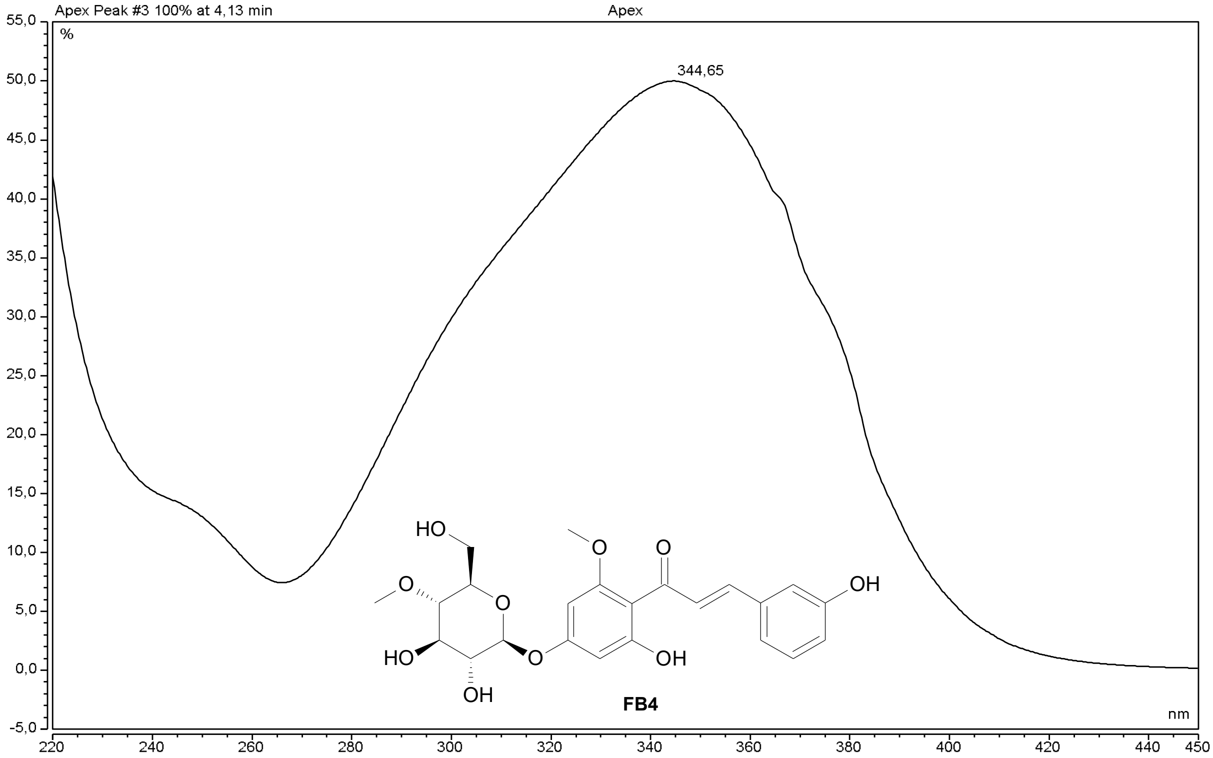

Figure S25. ^{1}H NMR spectra of 1-(4’-*O*-β-D-(4’’’-*O*-methylglucopiranosyl)-2’-hydroxy-6’-methoxyphenyl)-3-(3’’-hydoxyphenyl)-prop-2-en-1-on - 4’-*O*-β-D-(4″-*O*-methylglucopyranosyl)-3”-hydroxycardamonin (**FB4**) (DMSO-d_6, 600 MHz)

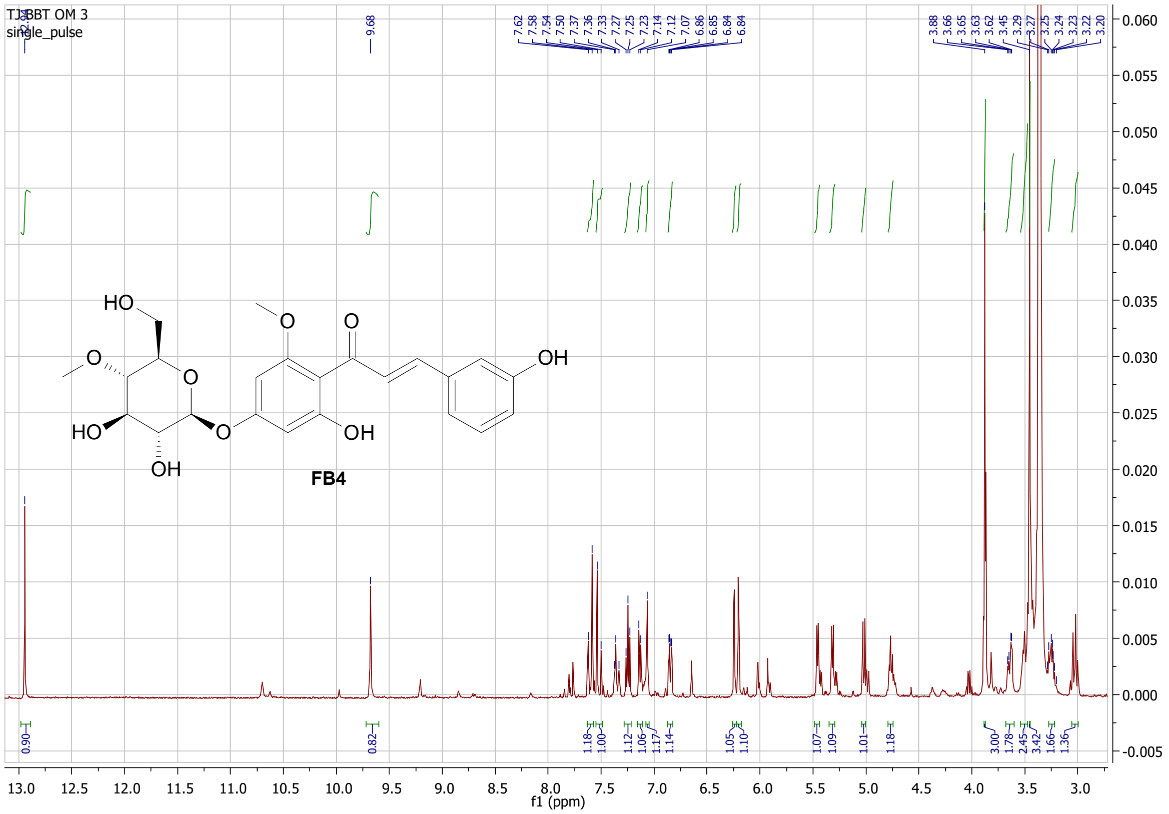

Figure S26. Flavone part of the ^{1}H NMR spectral 1-(4’-*O*-β-D-(4’’’-*O*-methylglucopiranosyl)-2’-hydroxy-6’-methoxyphenyl)-3-(3’’-hydoxyphenyl)-prop-2-en-1-on - 4’-*O*-β-D-(4″-*O*-methylglucopyranosyl)-3”-hydroxycardamonin (**FB4**) (DMSO-d_6, 600 MHz)

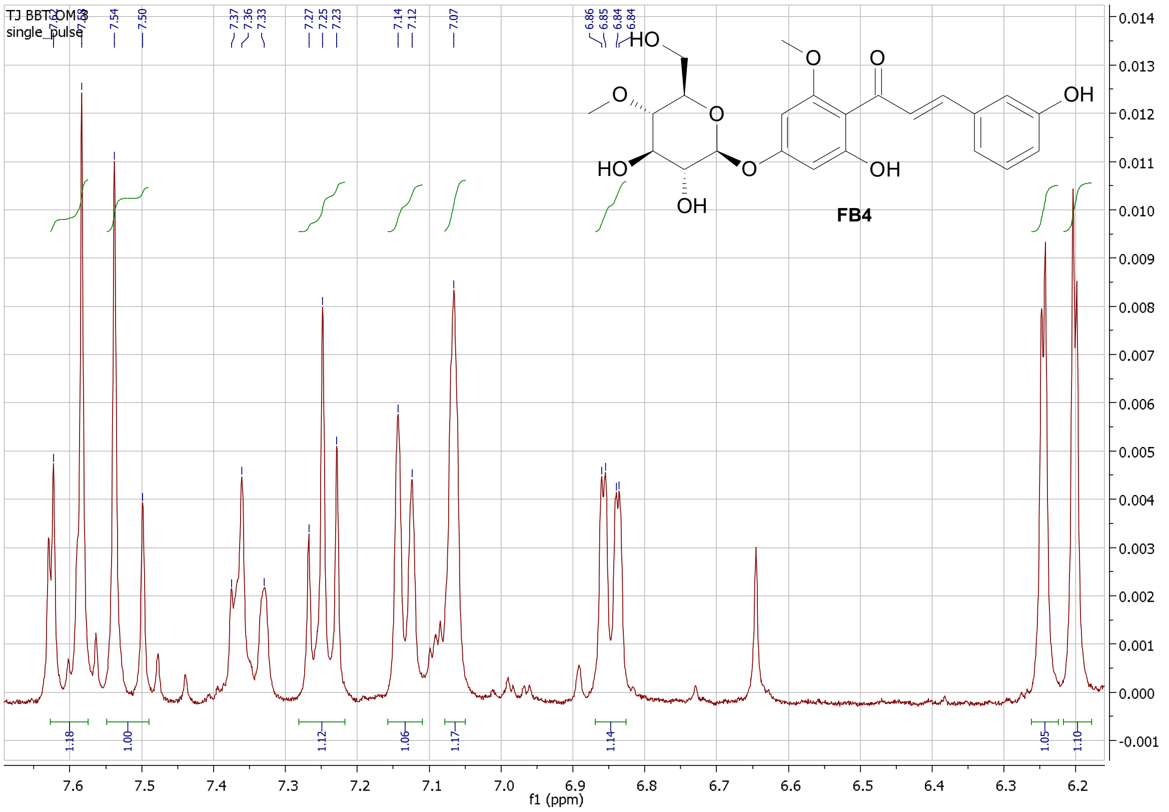

Figure S27. ^{13}C NMR spectra of 1-(4'-*O*-β-D-(4'''-*O*-methylglucopiranosyl)-2'-hydroxy-6'-methoxyphenyl)-3-(3''-hydoxyphenyl)-prop-2-en-1-on - 4'-*O*-β-D-(4″-*O*-methylglucopyranosyl)-3''-hydroxycardamonin (**FB4**) (DMSO-d_6, 151 MHz)

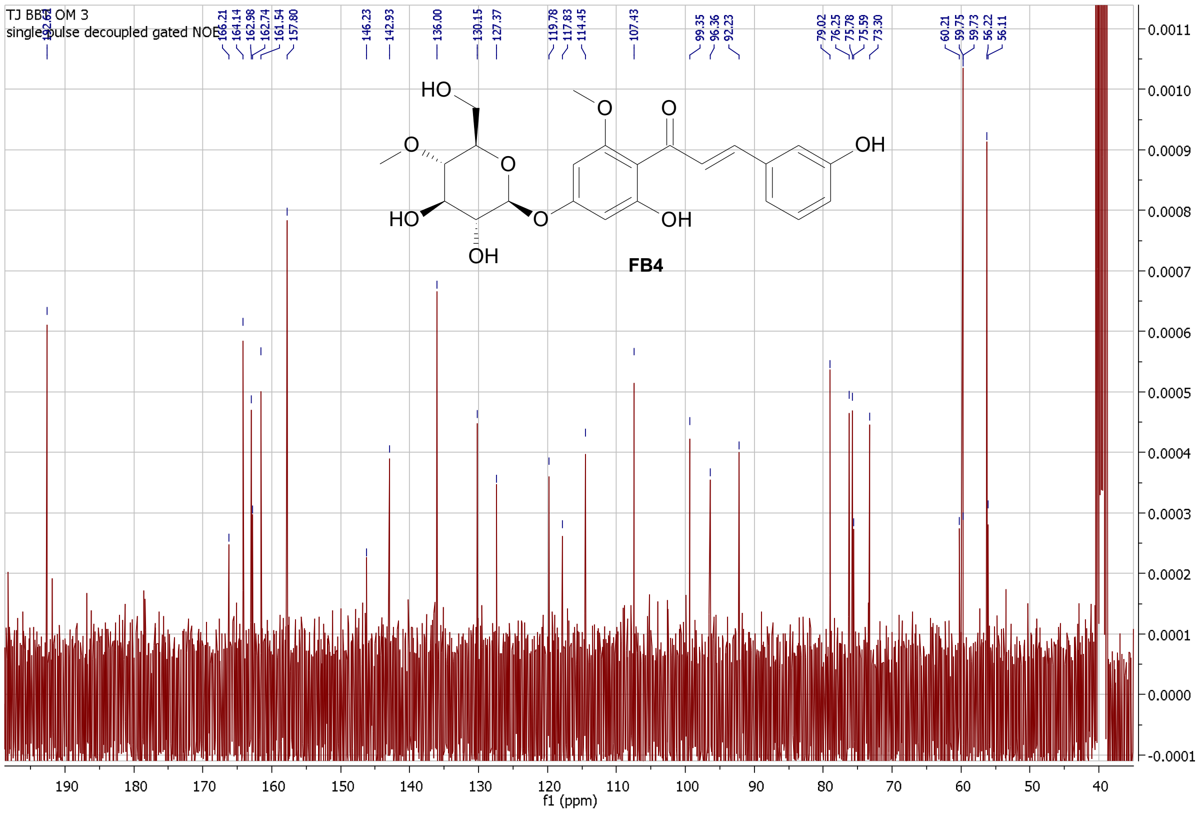

Figure S28. COSY spectrum of 1-(4'-*O*-β-D-(4'''-*O*-methylglucopiranosyl)-2'-hydroxy-6'-methoxyphenyl)-3-(3''-hydoxyphenyl)-prop-2-en-1-on - 4'-*O*-β-D-(4″-*O*-methylglucopyranosyl)-3''-hydroxycardamonin (**FB4**) (DMSO-d_6, 600 MHz)

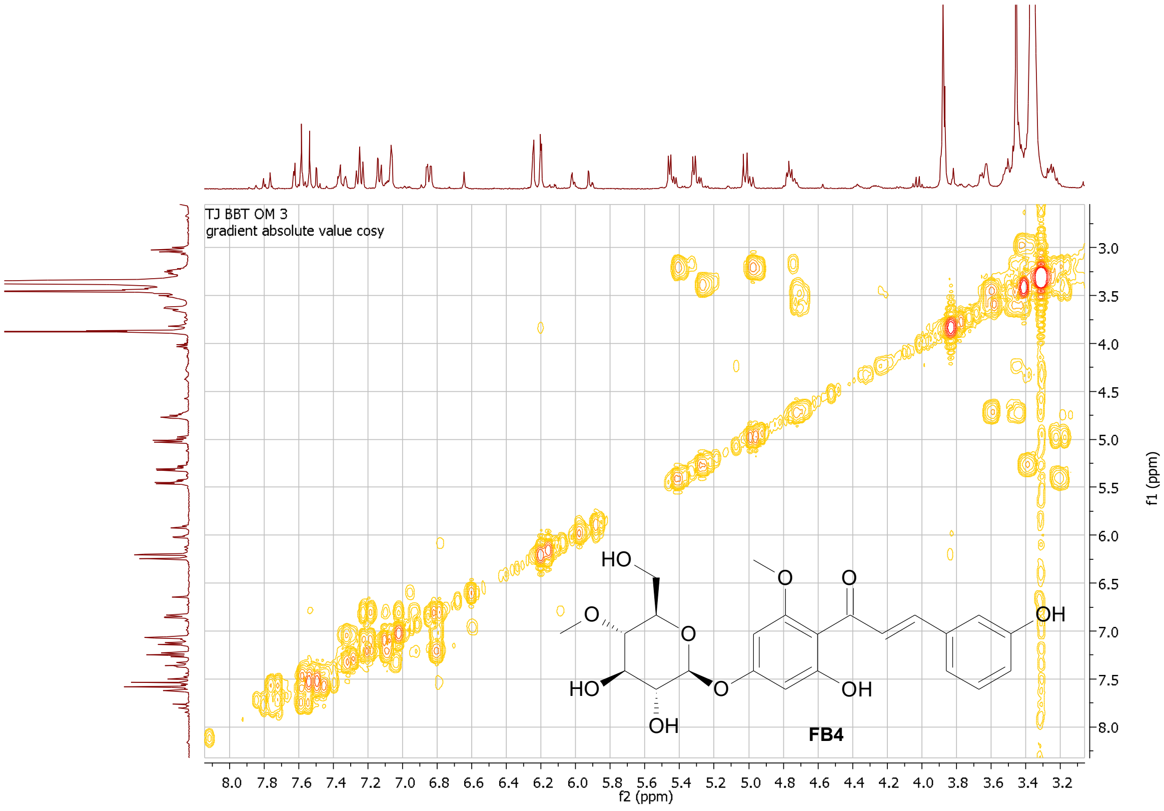

Figure S29. HSQC spectrum of 1-(4’-*O*-β-D-(4’’’-*O*-methylglucopiranosyl)-2’-hydroxy-6’-methoxyphenyl)-3-(3’’-hydoxyphenyl)-prop-2-en-1-on - 4’-*O*-β-D-(4″-*O*-methylglucopyranosyl)-3”-hydroxycardamonin (**FB4**) (DMSO-d_6, 600/151 MHz)

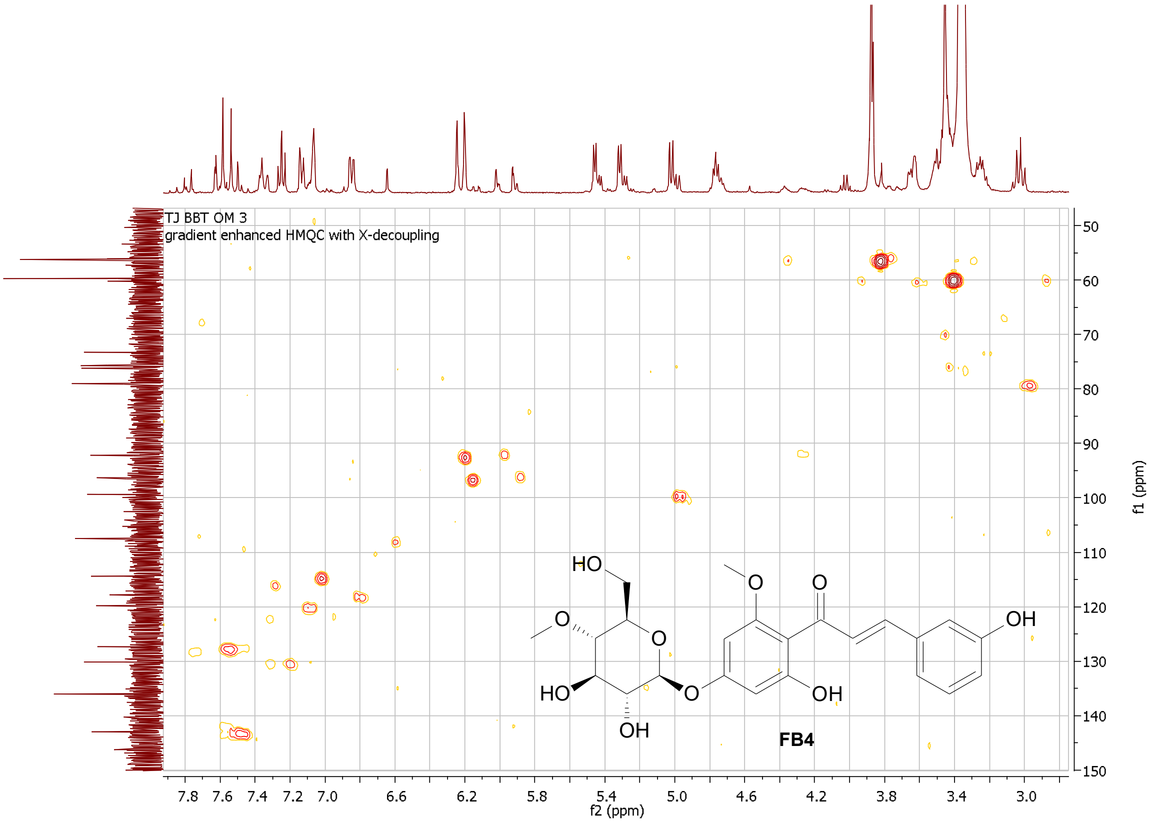

Figure S30. HMBC spectrum of 1-(4’-*O*-β-D-(4’’’-*O*-methylglucopiranosyl)-2’-hydroxy-6’-methoxyphenyl)-3-(3’’-hydoxyphenyl)-prop-2-en-1-on - 4’-*O*-β-D-(4″-*O*-methylglucopyranosyl)-3”-hydroxycardamonin (**FB4**) (DMSO-d_6, 600/151 MHz)

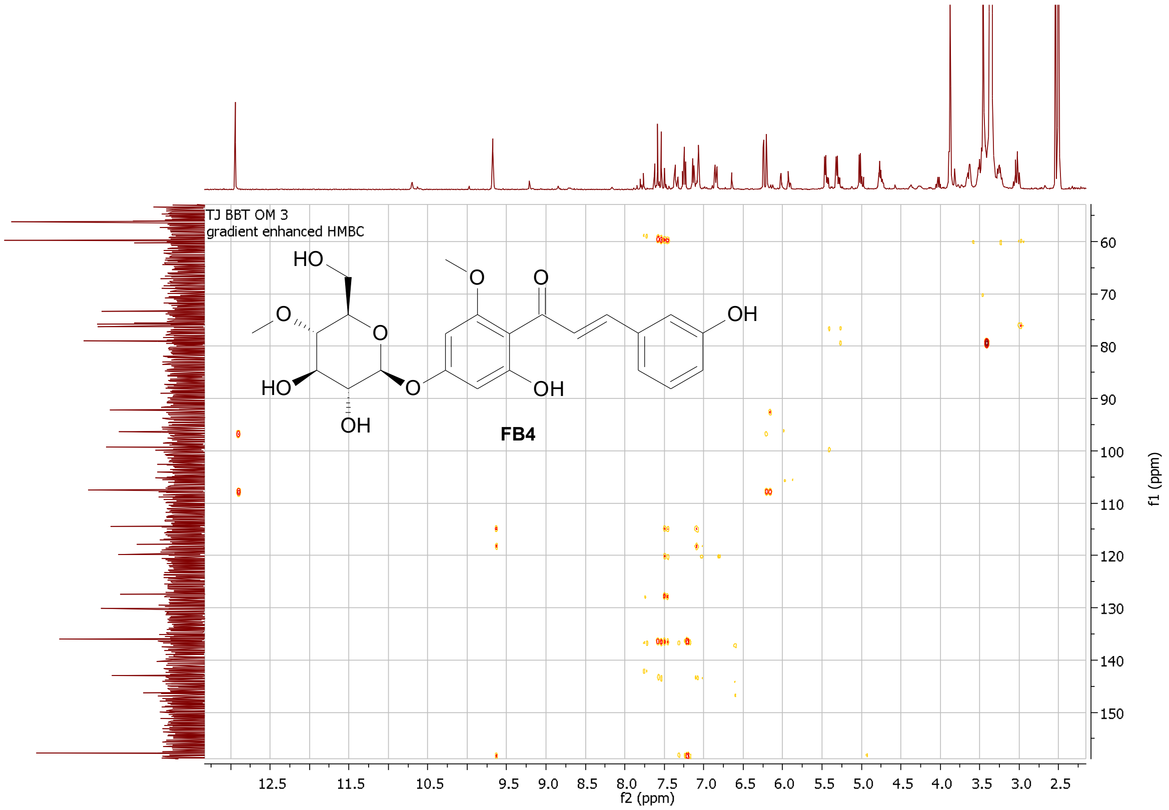

Figure S31. Predicted Boiled-Egg plot from swissADME online web tool for 1-(4’-*O*-β-D-(4’’’-*O*-methylglucopiranosyl)-2’-hydroxy-6’-methoxyphenyl)-3-(3’’-hydoxyphenyl)-prop-2-en-1-on - 4’-*O*-β-D-(4″-*O*-methylglucopyranosyl)-3’’-hydroxycardamonin (**FB4**)

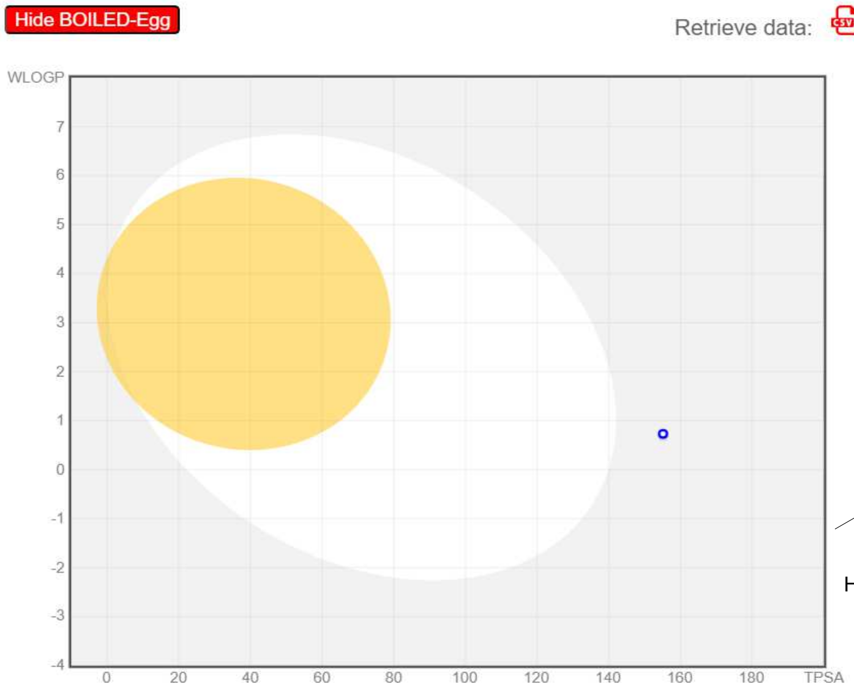

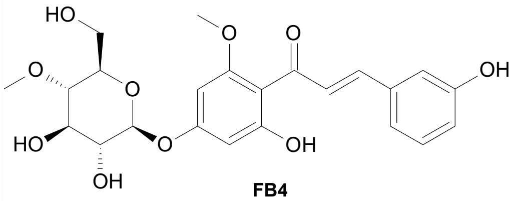

FB4

Figure S32. 1-(4’-*O*-β-D-(4’’’-*O*-methylglucopiranosyl)-2’-hydroxy-6’-methoxyphenyl)-3-(3’’-hydoxyphenyl)-prop-2-en-1-on - 4’-*O*-β-D-(4″-*O*-methylglucopyranosyl)-3’’-hydroxycardamonin (**FB4**) physicochemical and ADME parameters prediction using the SwissADME modelling

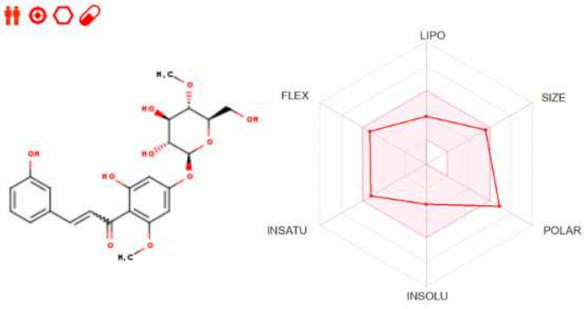

SMILES CO[C@@H]1[C@@H](CO)O[C@H]([C@@H]([C@H]1O)O)Oc1cc(O)c(c(c1)OC)C(=O)/C=C/c1cccc(c1)O

Physicochemical Properties	
Formula	C23H26O10
Molecular weight	462.45 g/mol
Num. heavy atoms	33
Num. arom. heavy atoms	12
Fraction Csp3	0.35
Num. rotatable bonds	8
Num. H-bond acceptors	10
Num. H-bond donors	5
Molar Refractivity	115.66
TPSA	155.14 Å²

Lipophilicity	
Log $P_{o/w}$ (iLOGP)	2.15
Log $P_{o/w}$ (XLOGP3)	1.33
Log $P_{o/w}$ (WLOGP)	0.73
Log $P_{o/w}$ (MLOGP)	-1.06
Log $P_{o/w}$ (SILICOS-IT)	1.05
Consensus Log $P_{o/w}$	0.84

Water Solubility	
Log *S* (ESOL)	-3.29
Solubility	2.39e-01 mg/ml ; 5.17e-04 mol/l
Class	Soluble
Log *S* (Ali)	-4.19
Solubility	2.99e-02 mg/ml ; 6.47e-05 mol/l
Class	Moderately soluble
Log *S* (SILICOS-IT)	-2.32
Solubility	2.22e+00 mg/ml ; 4.81e-03 mol/l
Class	Soluble

Pharmacokinetics	
GI absorption	Low
BBB permeant	No
P-gp substrate	Yes
CYP1A2 inhibitor	No
CYP2C19 inhibitor	No
CYP2C9 inhibitor	No
CYP2D6 inhibitor	No
CYP3A4 inhibitor	No
Log K_p (skin permeation)	-8.18 cm/s

Druglikeness	
Lipinski	Yes; 0 violation
Ghose	Yes
Veber	No; 1 violation: TPSA>140
Egan	No; 1 violation: TPSA>131.6
Muegge	No; 1 violation: TPSA>150
Bioavailability Score	0.55

Medicinal Chemistry	
PAINS	0 alert
Brenk	1 alert: michael_acceptor_1
Leadlikeness	No; 2 violations: MW>350, Rotors>7
Synthetic accessibility	5.24

Figure S33. The UV absorption maxima of 1-(4’-*O*-β-D-(4’”-*O*-methylglucopiranosyl)-2’-hydroxy-6’-methoxyphenyl)-3-(4”-hydoxyphenyl)-prop-2-en-1-on - 4’-*O*-β-D-(4″-*O*-methylglucopyranosyl)-4”-hydroxycardamonin (**FB5**)

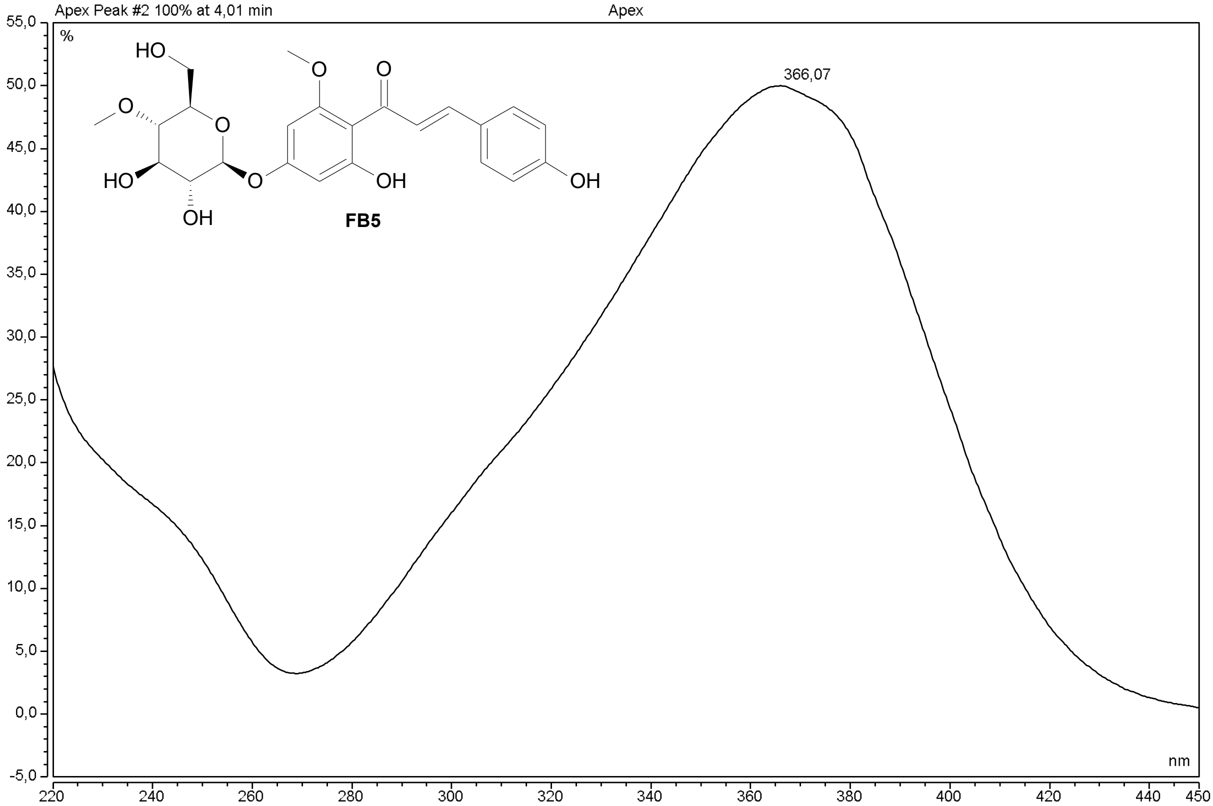

Figure S34. ^{1}H NMR spectra of 1-(4’-*O*-β-D-(4’”-*O*-methylglucopiranosyl)-2’-hydroxy-6’-methoxyphenyl)-3-(4”-hydoxyphenyl)-prop-2-en-1-on - 4’-*O*-β-D-(4″-*O*-methylglucopyranosyl)-4”-hydroxycardamonin (**FB5**) (Acetone-d_6, 600 MHz)

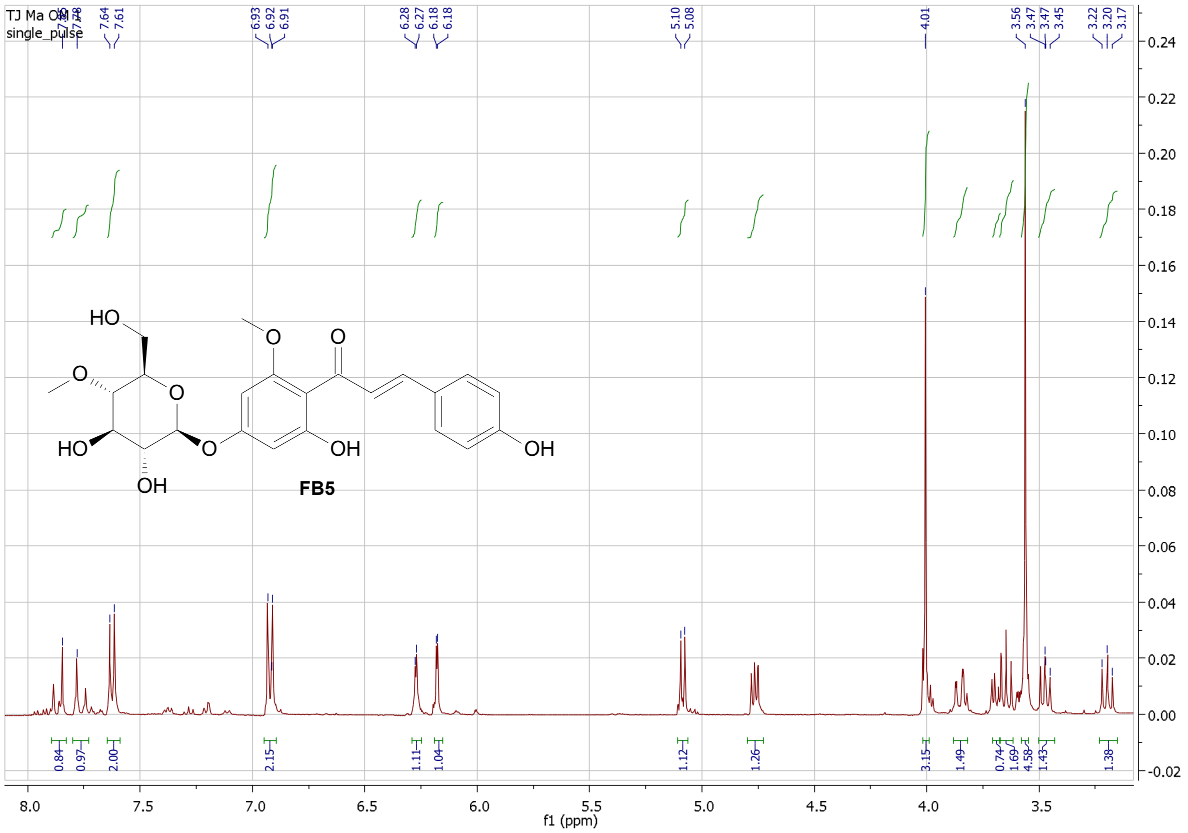

Figure S35. Flavone part of the ^{1}H NMR spectral 1-(4’-*O*-β-D-(4’’’-*O*-methylglucopiranosyl)-2’-hydroxy-6’-methoxyphenyl)-3-(4’’-hydoxyphenyl)-prop-2-en-1-on - 4’-*O*-β-D-(4″-*O*-methylglucopyranosyl)-4”-hydroxycardamonin (**FB5**) (Acetone-d_6, 600 MHz)

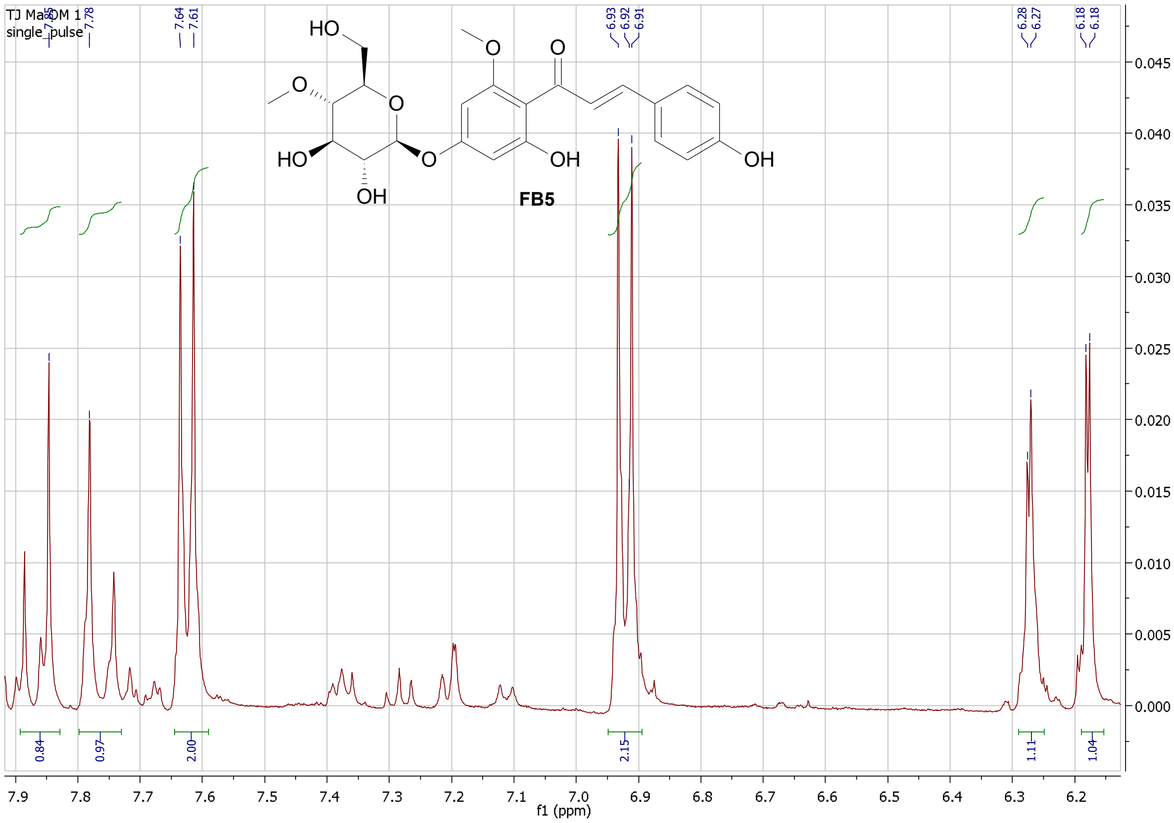

Figure S36. ^{13}C NMR spectra of 1-(4’-*O*-β-D-(4’’’-*O*-methylglucopiranosyl)-2’-hydroxy-6’-methoxyphenyl)-3-(4’’-hydoxyphenyl)-prop-2-en-1-on - 4’-*O*-β-D-(4″-*O*-methylglucopyranosyl)-4”-hydroxycardamonin (**FB5**) (Acetone-d_6, 151 MHz)

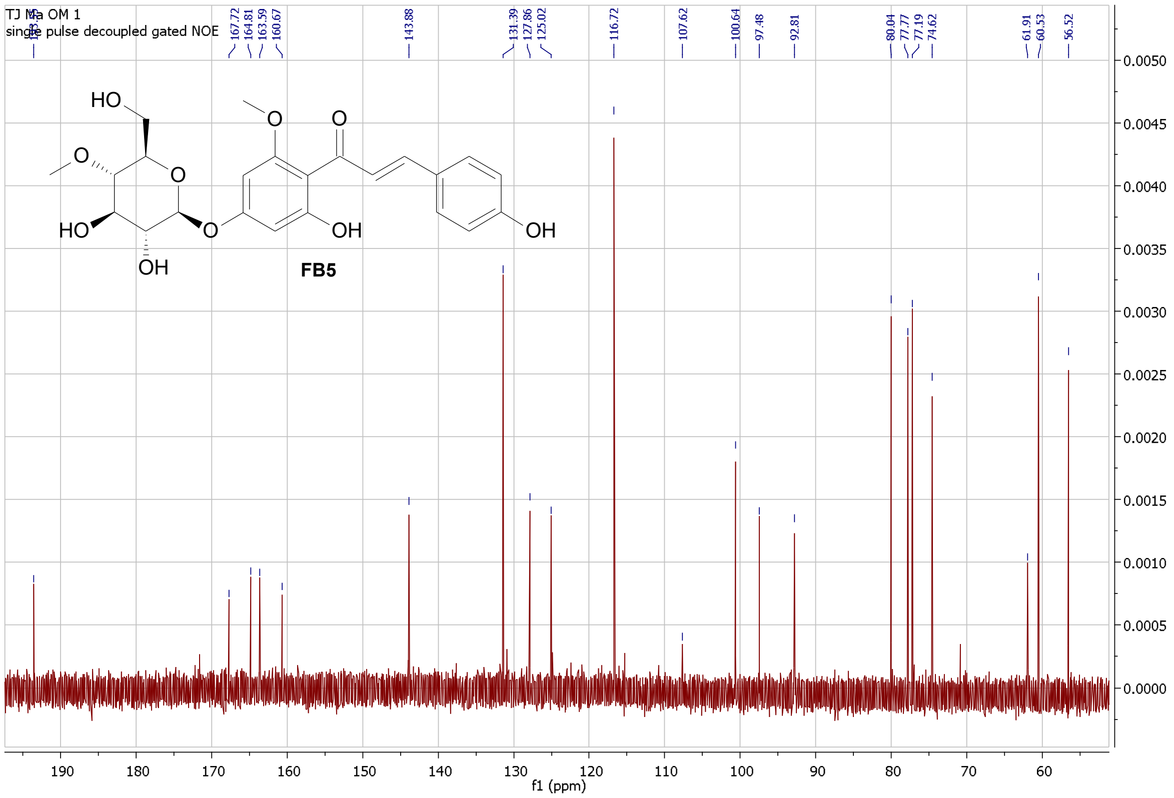

Figure S37. COSY spectrum of 1-(4’-*O*-β-D-(4’’’-*O*-methylglucopiranosyl)-2’-hydroxy-6’-methoxyphenyl)-3-(4’’-hydoxyphenyl)-prop-2-en-1-on - 4’-*O*-β-D-(4″-*O*-methylglucopyranosyl)-4’’-hydroxycardamonin (**FB5**) (Acetone-d_6, 600 MHz)

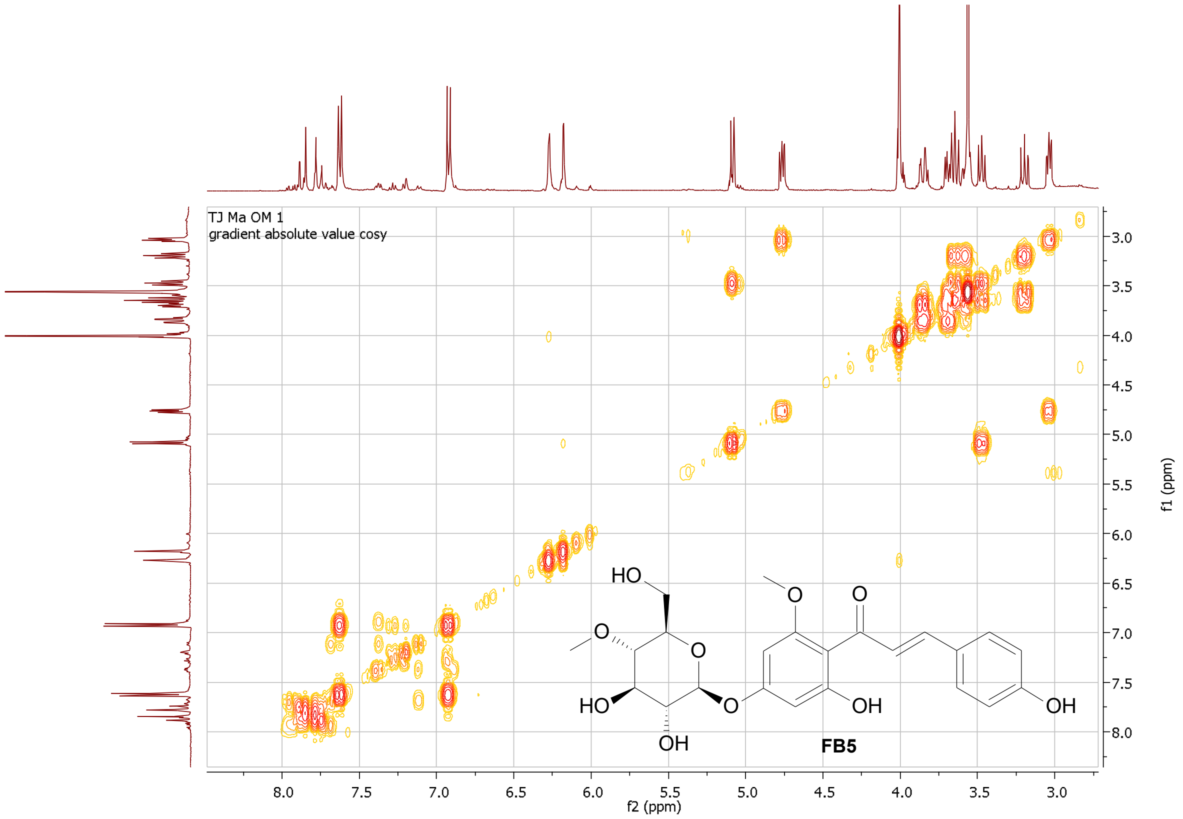

Figure S38. HSQC spectrum of 1-(4’-*O*-β-D-(4’’’-*O*-methylglucopiranosyl)-2’-hydroxy-6’-methoxyphenyl)-3-(4’’-hydoxyphenyl)-prop-2-en-1-on - 4’-*O*-β-D-(4″-*O*-methylglucopyranosyl)-4’’-hydroxycardamonin (**FB5**) (Acetone-d_6, 600/151 MHz)

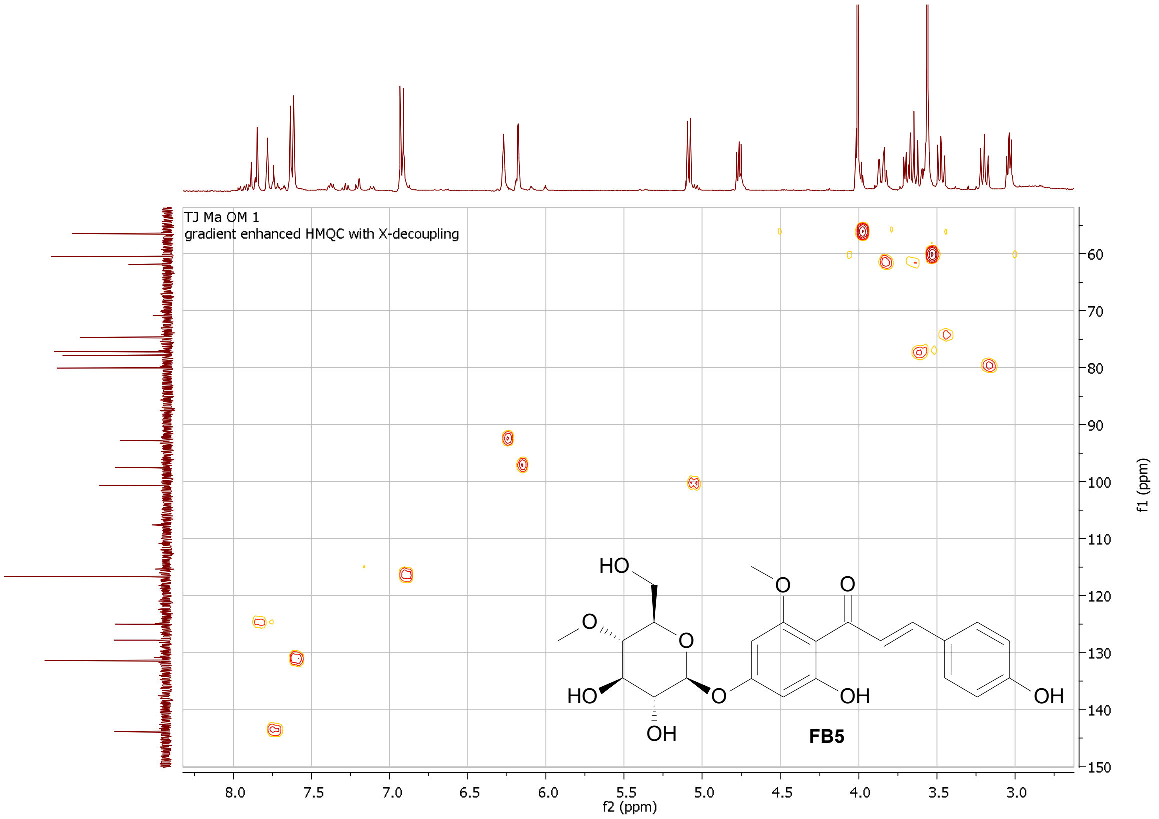

Figure S39. HMBC spectrum of 1-(4’-*O*-β-D-(4’”-*O*-methylglucopiranosyl)-2’-hydroxy-6’-methoxyphenyl)-3-(4”-hydoxyphenyl)-prop-2-en-1-on - 4’-*O*-β-D-(4″-*O*-methylglucopyranosyl)-4”-hydroxycardamonin (**FB5**) (Acetone-d_6, 600/151 MHz)

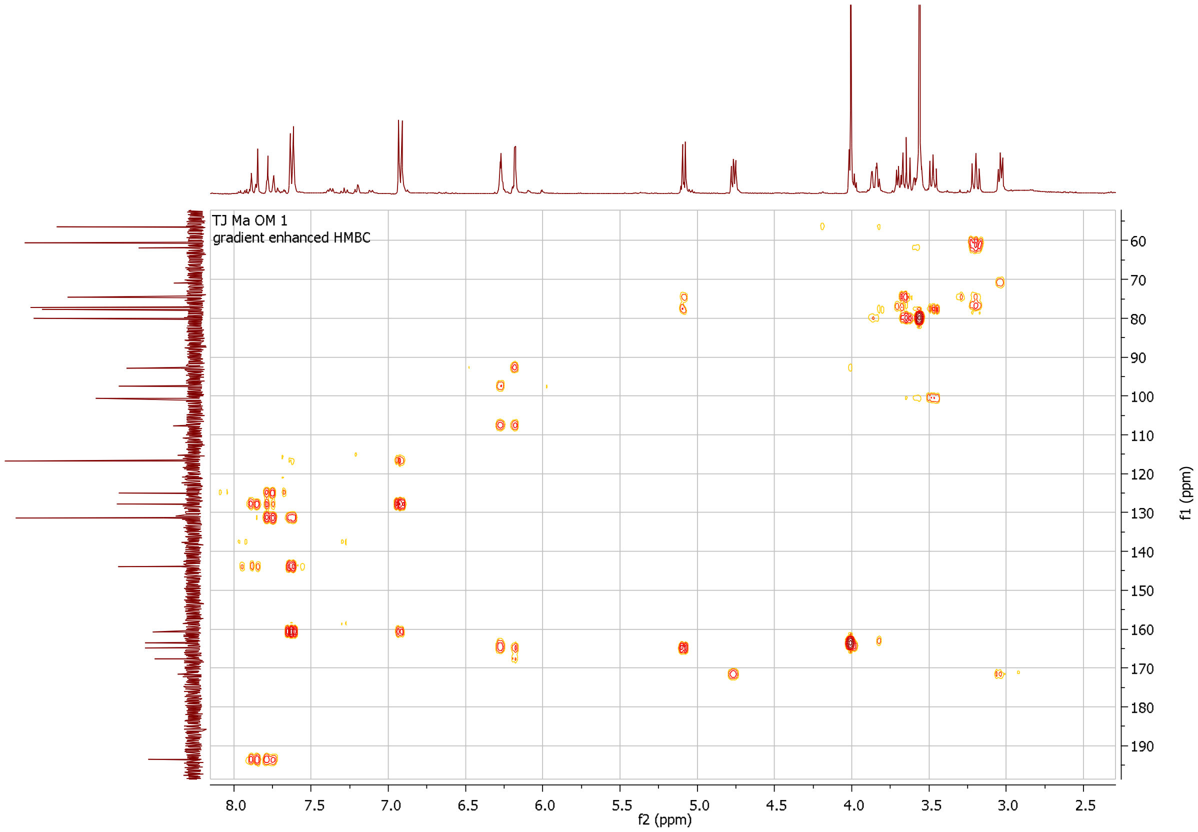

Figure S40. Predicted Boiled-Egg plot from swissADME online web tool for 1-(4’-*O*-β-D-(4’”-*O*-methylglucopiranosyl)-2’-hydroxy-6’-methoxyphenyl)-3-(4”-hydoxyphenyl)-prop-2-en-1-on - 4’-*O*-β-D-(4″-*O*-methylglucopyranosyl)-4”-hydroxycardamonin (**FB5**)

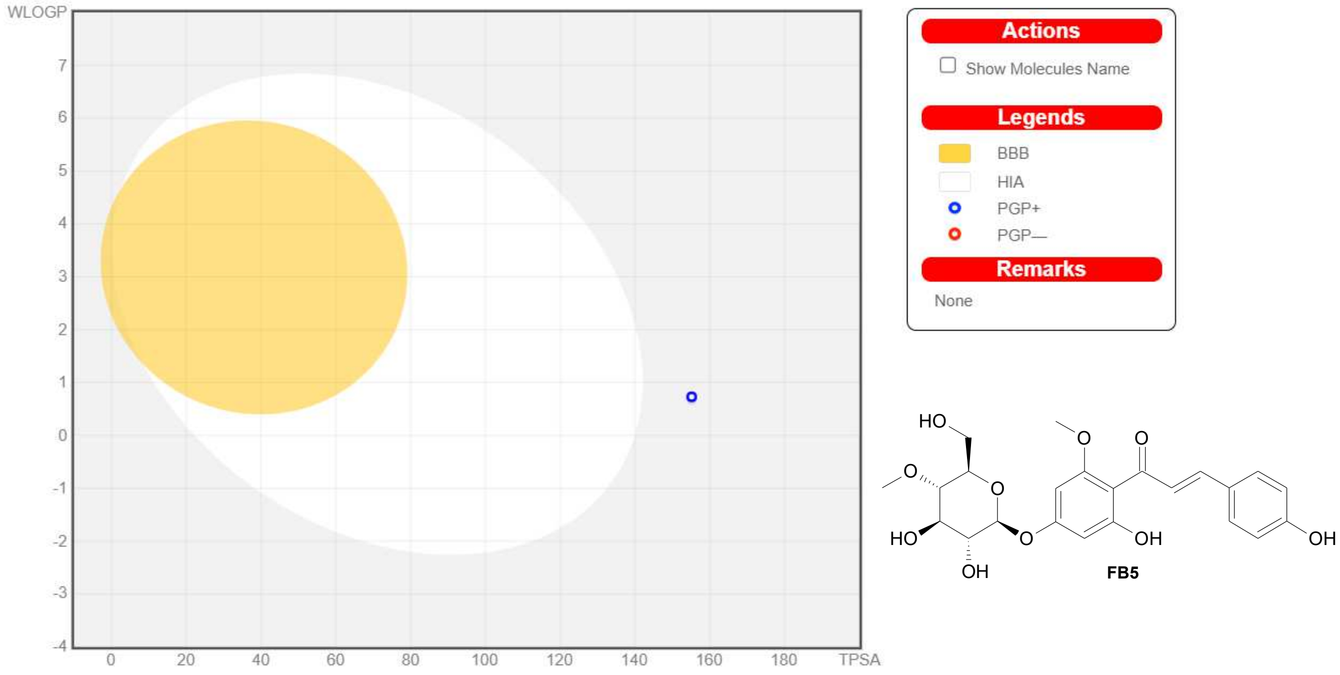

Figure S41. 1-(4'-*O*-β-D-(4'''-*O*-methylglucopiranosyl)-2'-hydroxy-6'-methoxyphenyl)-3-(4''-hydoxyphenyl)-prop-2-en-1-on - 4'-*O*-β-D-(4″-*O*-methylglucopyranosyl)-4''-hydroxycardamonin (**FB5**) physicochemical and ADME parameters prediction using the SwissADME modelling

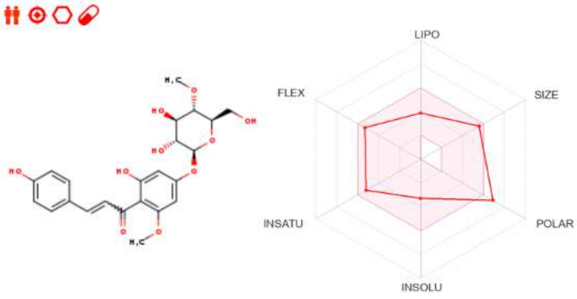

SMILES	CO[C@@H]1[C@@H](CO)O[C@H]([C@@H]([C@H]1O)O)Oc1cc(O)c(c(c1)OC)C(=O)/C=C/c1ccc(cc1)O

Physicochemical Properties	
Formula	C23H26O10
Molecular weight	462.45 g/mol
Num. heavy atoms	33
Num. arom. heavy atoms	12
Fraction Csp3	0.35
Num. rotatable bonds	8
Num. H-bond acceptors	10
Num. H-bond donors	5
Molar Refractivity	115.66
TPSA	155.14 Å²

Lipophilicity	
Log $P_{o/w}$ (iLOGP)	2.11
Log $P_{o/w}$ (XLOGP3)	1.33
Log $P_{o/w}$ (WLOGP)	0.73
Log $P_{o/w}$ (MLOGP)	-1.06
Log $P_{o/w}$ (SILICOS-IT)	1.05
Consensus Log $P_{o/w}$	0.83

Water Solubility	
Log *S* (ESOL)	-3.29
Solubility	2.39e-01 mg/ml ; 5.17e-04 mol/l
Class	Soluble
Log *S* (Ali)	-4.19
Solubility	2.99e-02 mg/ml ; 6.47e-05 mol/l
Class	Moderately soluble
Log *S* (SILICOS-IT)	-2.32
Solubility	2.22e+00 mg/ml ; 4.81e-03 mol/l
Class	Soluble

Pharmacokinetics	
GI absorption	Low
BBB permeant	No
P-gp substrate	Yes
CYP1A2 inhibitor	No
CYP2C19 inhibitor	No
CYP2C9 inhibitor	No
CYP2D6 inhibitor	No
CYP3A4 inhibitor	No
Log K_p (skin permeation)	-8.18 cm/s

Druglikeness	
Lipinski	Yes; 0 violation
Ghose	Yes
Veber	No; 1 violation: TPSA>140
Egan	No; 1 violation: TPSA>131.6
Muegge	No; 1 violation: TPSA>150
Bioavailability Score	0.55

Medicinal Chemistry	
PAINS	0 alert
Brenk	1 alert: michael_acceptor_1
Leadlikeness	No; 2 violations: MW>350, Rotors>7
Synthetic accessibility	5.21

Figure S42. The UV absorption maxima of 1-(2'-hydroxy-4',6'-dimethoxyphenyl)-3-(3''-hydroxyphenyl)-prop-2-en-1-on - 3''-hydroxyflavokawain B (**FB6**)

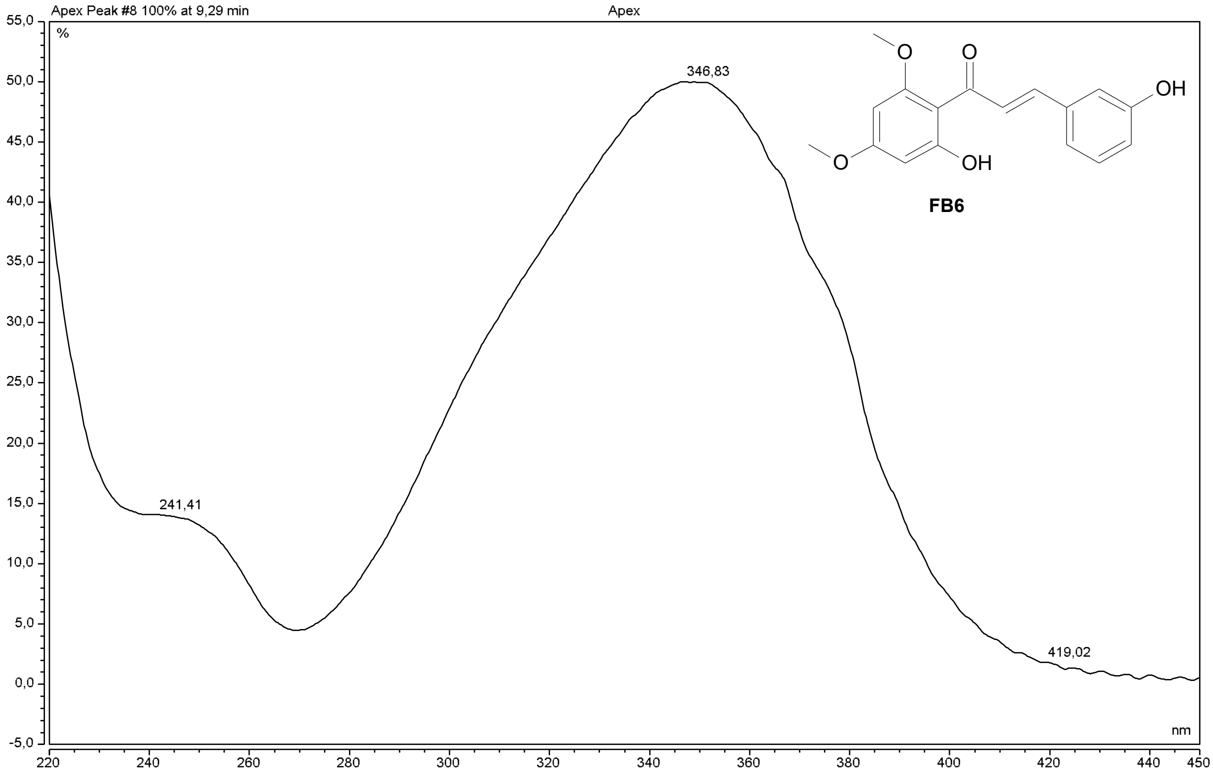

Figure S43. ^{1}H NMR spectra of 1-(2'-hydroxy-4',6'-dimethoxyphenyl)-3-(3''-hydroxyphenyl)-prop-2-en-1-on - 3''-hydroxyflavokawain B (**FB6**) (CDCl$_3$, 600 MHz)

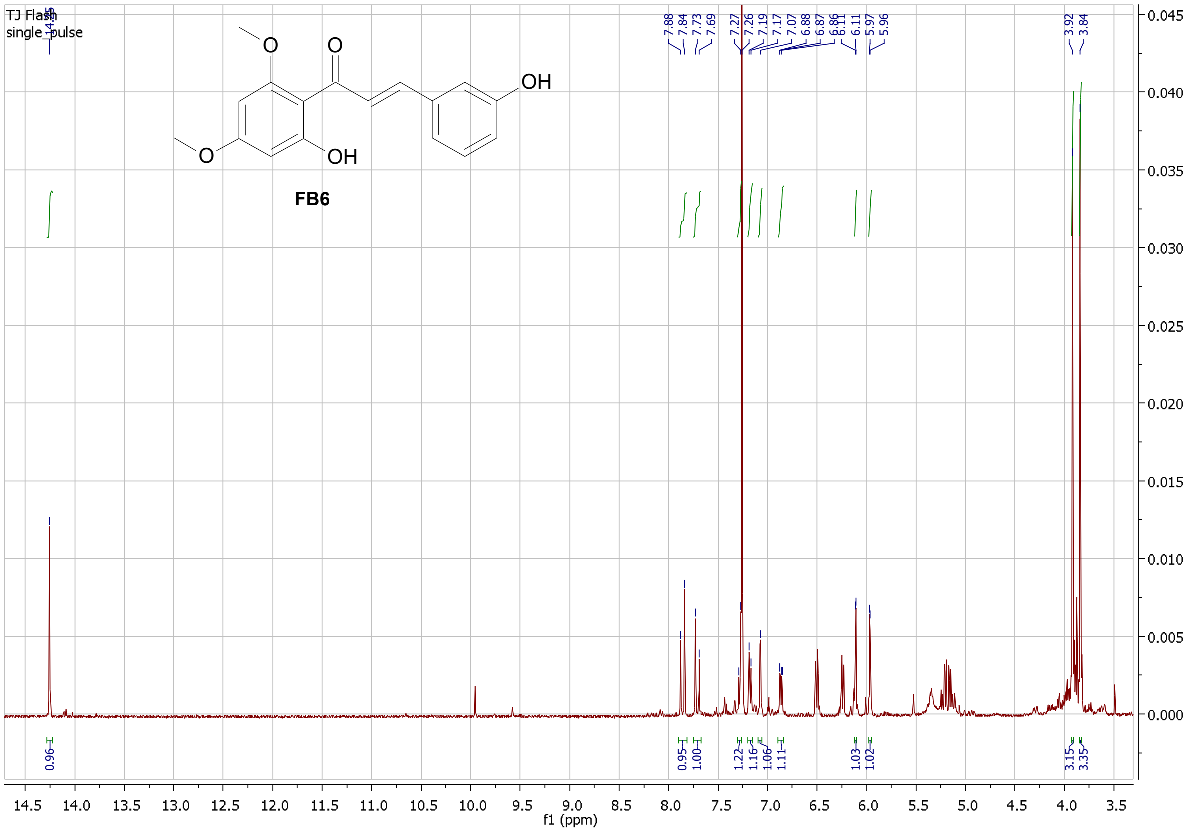

Figure S44. Flavone part of the ^{1}H NMR spectral 1-(2'-hydroxy-4',6'-dimethoxyphenyl)-3-(3''-hydroxyphenyl)-prop-2-en-1-on - 3''-hydroxyflavokawain B (**FB6**) (CDCl$_3$, 600 MHz)

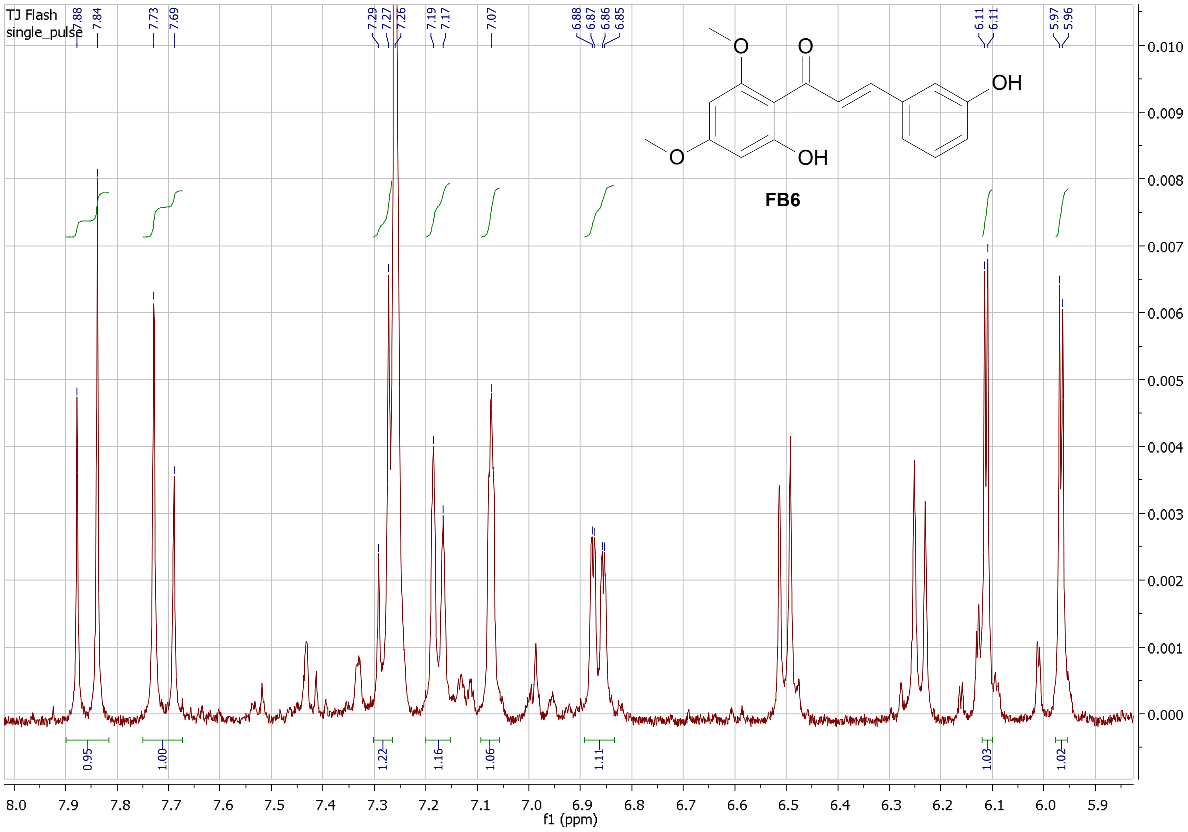

Figure S45. Predicted Boiled-Egg plot from swissADME online web tool for 1-(2'-hydroxy-4',6'-dimethoxyphenyl)-3-(3''-hydroxyphenyl)-prop-2-en-1-on - 3''-hydroxyflavokawain B (**FB6**)

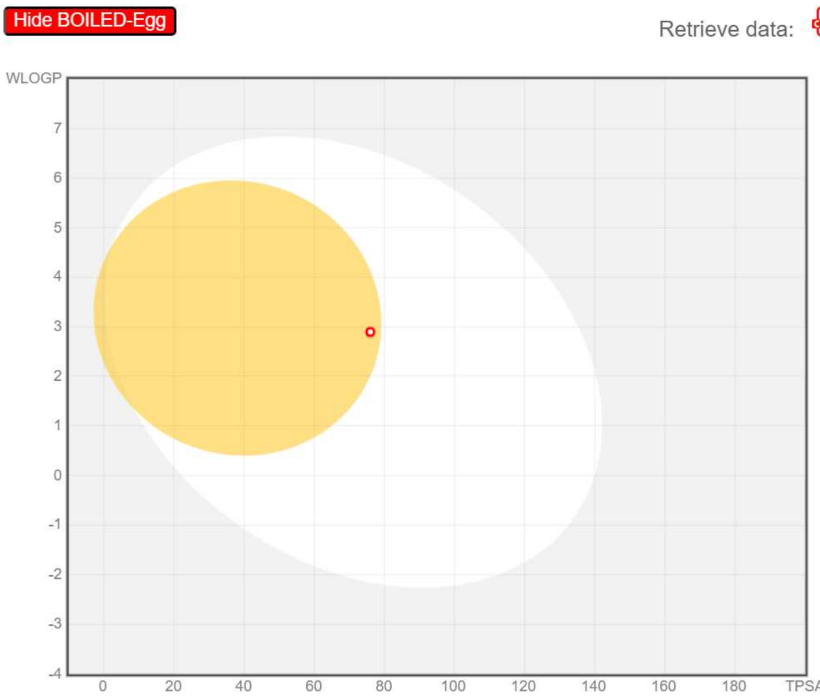

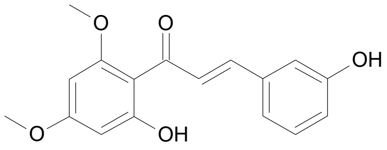

FB6

Figure S46. 1-(2'-hydroxy-4',6'-dimethoxyphenyl)-3-(3''-hydroxyphenyl)-prop-2-en-1-on - 3''-hydroxyflavokawain B (**FB6**) physicochemical and ADME parameters prediction using the SwissADME modelling

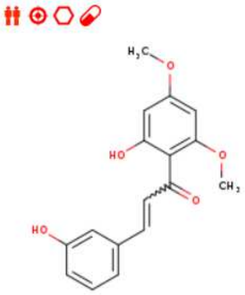

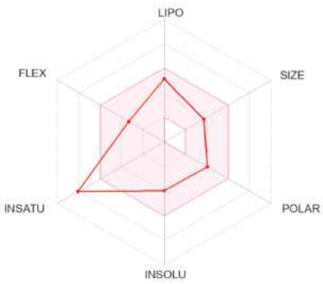

SMILES COc1cc(O)c(c(c1)OC)C(=O)/C=C/c1cccc(c1)O

Physicochemical Properties	
Formula	C17H16O5
Molecular weight	300.31 g/mol
Num. heavy atoms	22
Num. arom. heavy atoms	12
Fraction Csp3	0.12
Num. rotatable bonds	5
Num. H-bond acceptors	5
Num. H-bond donors	2
Molar Refractivity	83.28
TPSA	75.99 Å²
**Lipophilicity**	
Log $P_{o/w}$ (iLOGP)	2.43
Log $P_{o/w}$ (XLOGP3)	3.47
Log $P_{o/w}$ (WLOGP)	2.90
Log $P_{o/w}$ (MLOGP)	1.52
Log $P_{o/w}$ (SILICOS-IT)	3.06
Consensus Log $P_{o/w}$	2.68

Water Solubility	
Log *S* (ESOL)	-3.96
Solubility	3.28e-02 mg/ml ; 1.09e-04 mol/l
Class	Soluble
Log *S* (Ali)	-4.75
Solubility	5.37e-03 mg/ml ; 1.79e-05 mol/l
Class	Moderately soluble
Log *S* (SILICOS-IT)	-4.05
Solubility	2.69e-02 mg/ml ; 8.95e-05 mol/l
Class	Moderately soluble
**Pharmacokinetics**	
GI absorption	High
BBB permeant	Yes
P-gp substrate	No
CYP1A2 inhibitor	Yes
CYP2C19 inhibitor	No
CYP2C9 inhibitor	Yes
CYP2D6 inhibitor	No
CYP3A4 inhibitor	Yes
Log K_p (skin permeation)	-5.67 cm/s
**Druglikeness**	
Lipinski	Yes; 0 violation
Ghose	Yes
Veber	Yes
Egan	Yes
Muegge	Yes
Bioavailability Score	0.55
**Medicinal Chemistry**	
PAINS	0 alert
Brenk	1 alert: michael_acceptor_1
Leadlikeness	Yes
Synthetic accessibility	2.82

Figure S47. The UV absorption maxima of 1-(2’-hydroxy-4’,6’-dimethoxyphenyl)-3-(3’’-*O*-β-D-(4’’’-*O*-methylglucopiranosyl)-phenyl)-prop-2-en-1-on - 3’-*O*-β-D-(4″-*O*-methylglucopyranosyl)-flavokawain B (**FB7**)

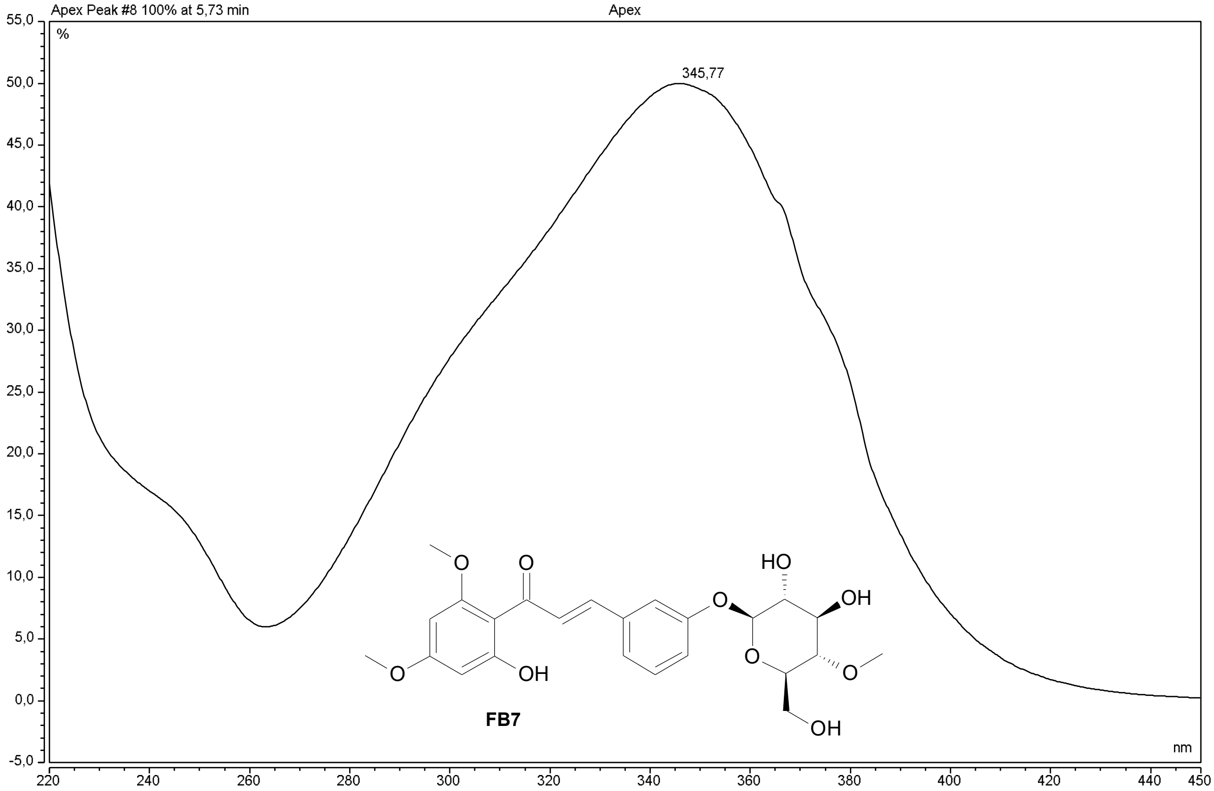

Figure S48. ^{1}H NMR spectra of 1-(2’-hydroxy-4’,6’-dimethoxyphenyl)-3-(3’’-*O*-β-D-(4’’’-*O*-methylglucopiranosyl)-phenyl)-prop-2-en-1-on - 3’-*O*-β-D-(4″-*O*-methylglucopyranosyl)-flavokawain B (**FB7**) (DMSO-d_6, 600 MHz)

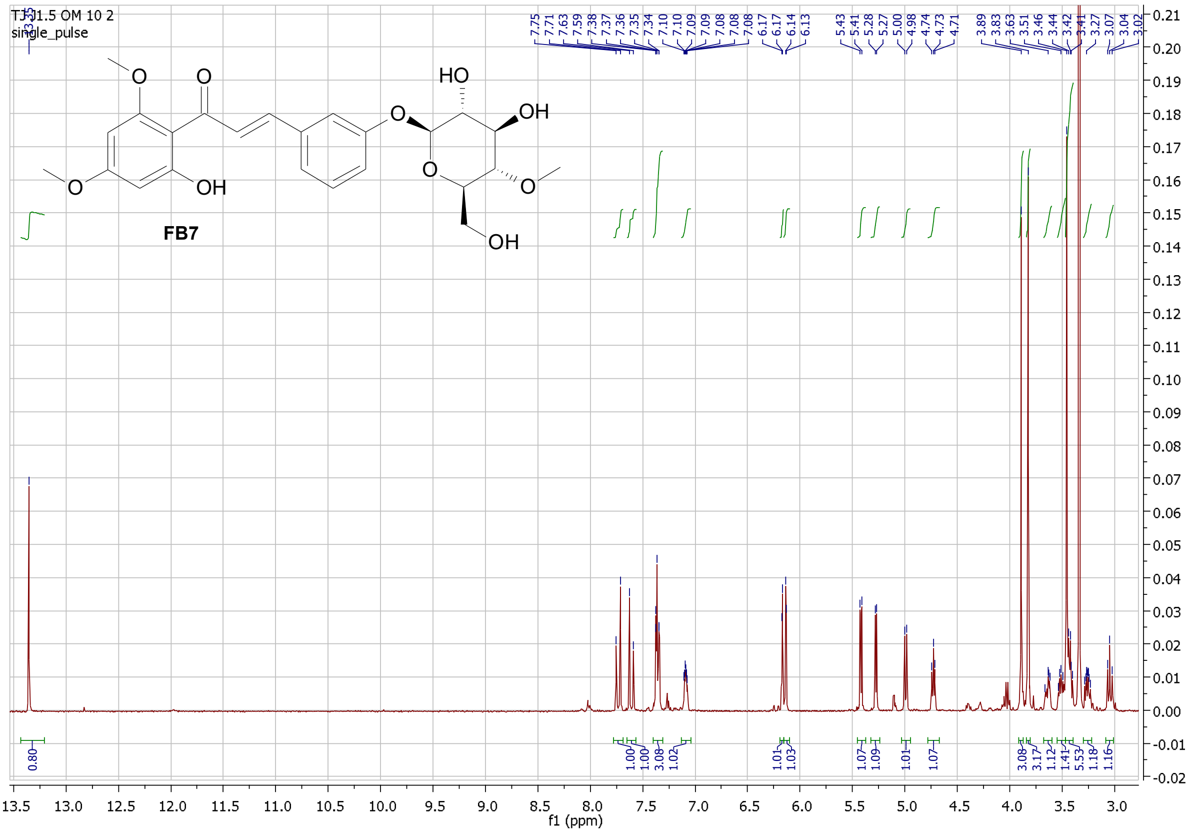

Figure S49. Flavone part of the ^{1}H NMR spectral 1-(2’-hydroxy-4’,6’-dimethoxyphenyl)-3-(3”-*O*-β-D-(4’”-*O*-methylglucopiranosyl)-phenyl)-prop-2-en-1-on - 3’-*O*-β-D-(4″-*O*-methylglucopyranosyl)-flavokawain B (**FB7**) (DMSO-*d*$_6$, 600 MHz)

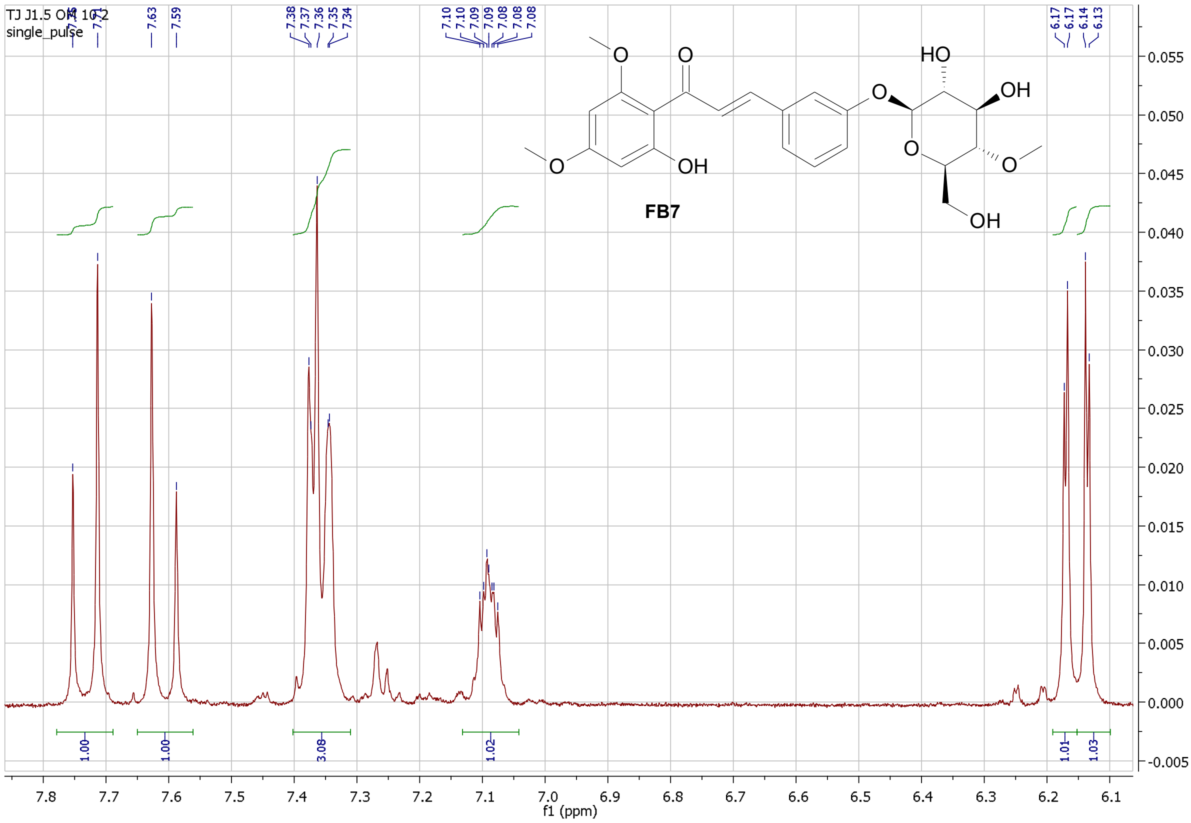

Figure S50. ^{13}C NMR spectra of 1-(2’-hydroxy-4’,6’-dimethoxyphenyl)-3-(3”-*O*-β-D-(4’”-*O*-methylglucopiranosyl)-phenyl)-prop-2-en-1-on - 3’-*O*-β-D-(4″-*O*-methylglucopyranosyl)-flavokawain B (**FB7**) (DMSO-*d*$_6$, 151 MHz)

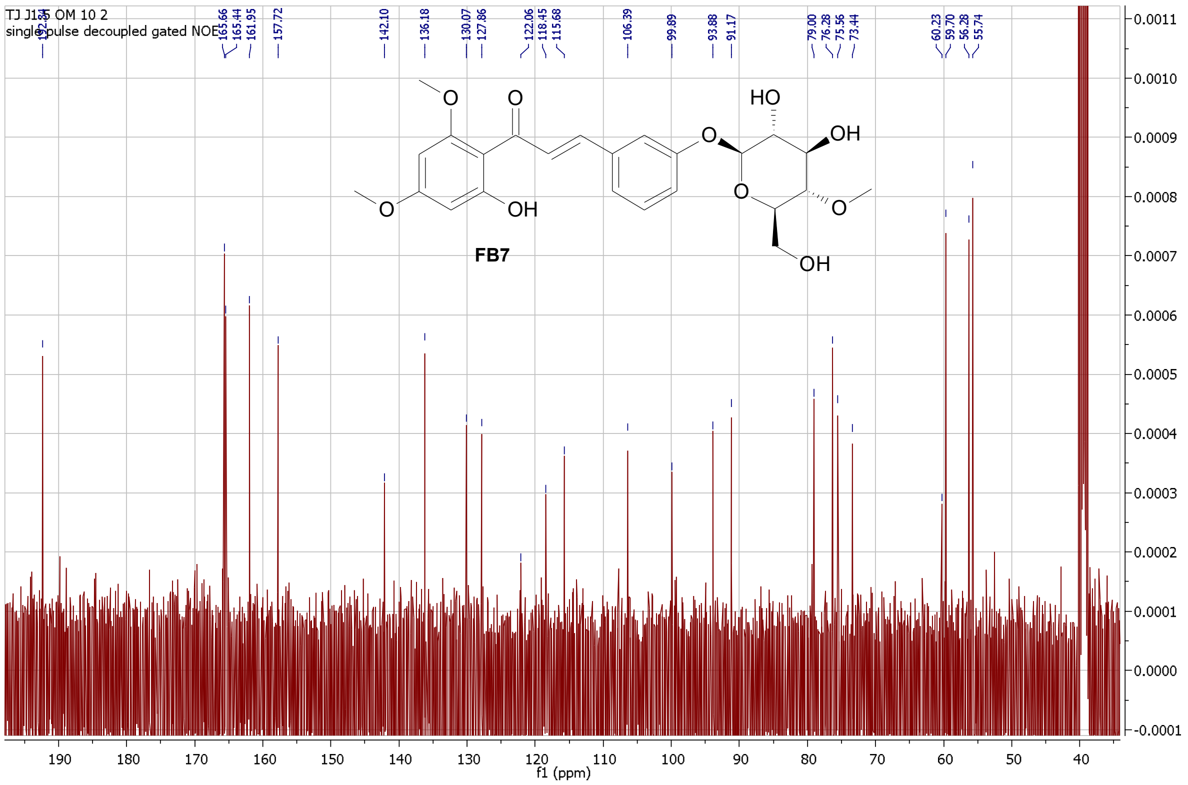

Figure S51. COSY spectrum of 1-(2'-hydroxy-4',6'-dimethoxyphenyl)-3-(3''-*O*-β-D-(4'''-*O*-methylglucopiranosyl)-phenyl)-prop-2-en-1-on - 3'-*O*-β-D-(4″-*O*-methylglucopyranosyl)-flavokawain B (**FB7**) (DMSO-d_6, 600 MHz)

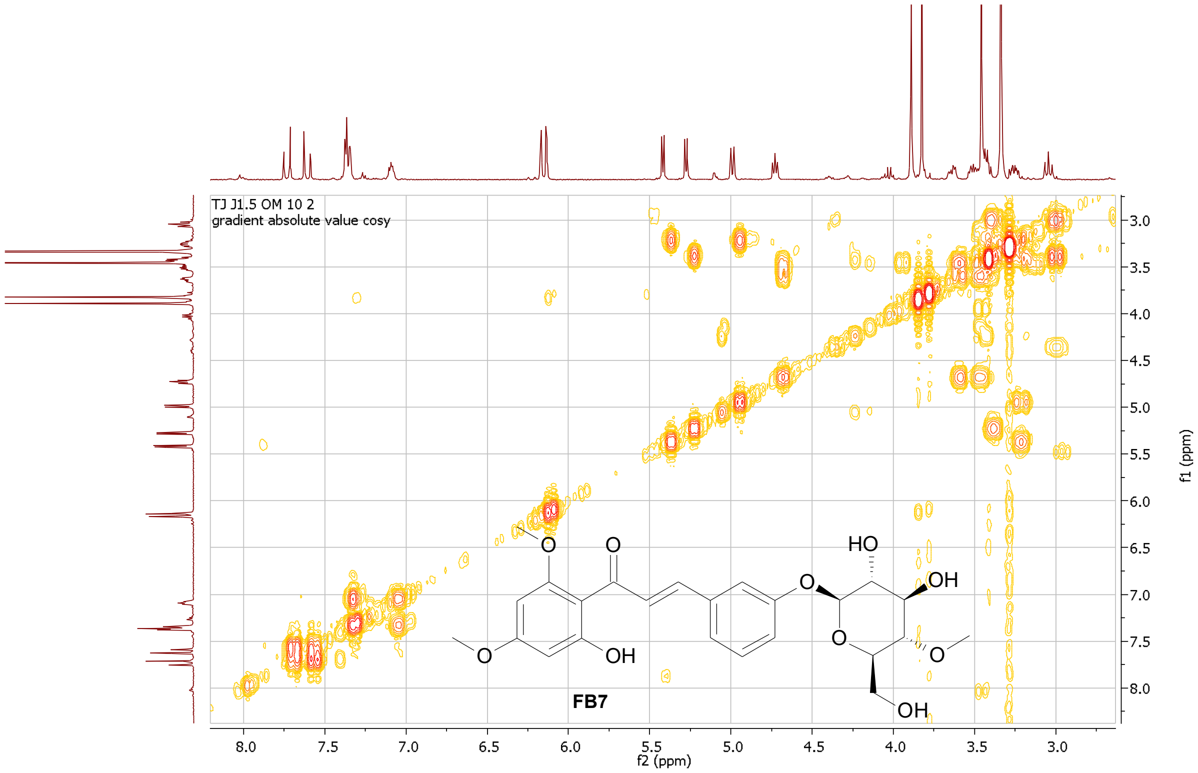

Figure S52. HSQC spectrum of 1-(2'-hydroxy-4',6'-dimethoxyphenyl)-3-(3''-*O*-β-D-(4'''-*O*-methylglucopiranosyl)-phenyl)-prop-2-en-1-on - 3'-*O*-β-D-(4″-*O*-methylglucopyranosyl)-flavokawain B (**FB7**) (DMSO-d_6, 600/151 MHz)

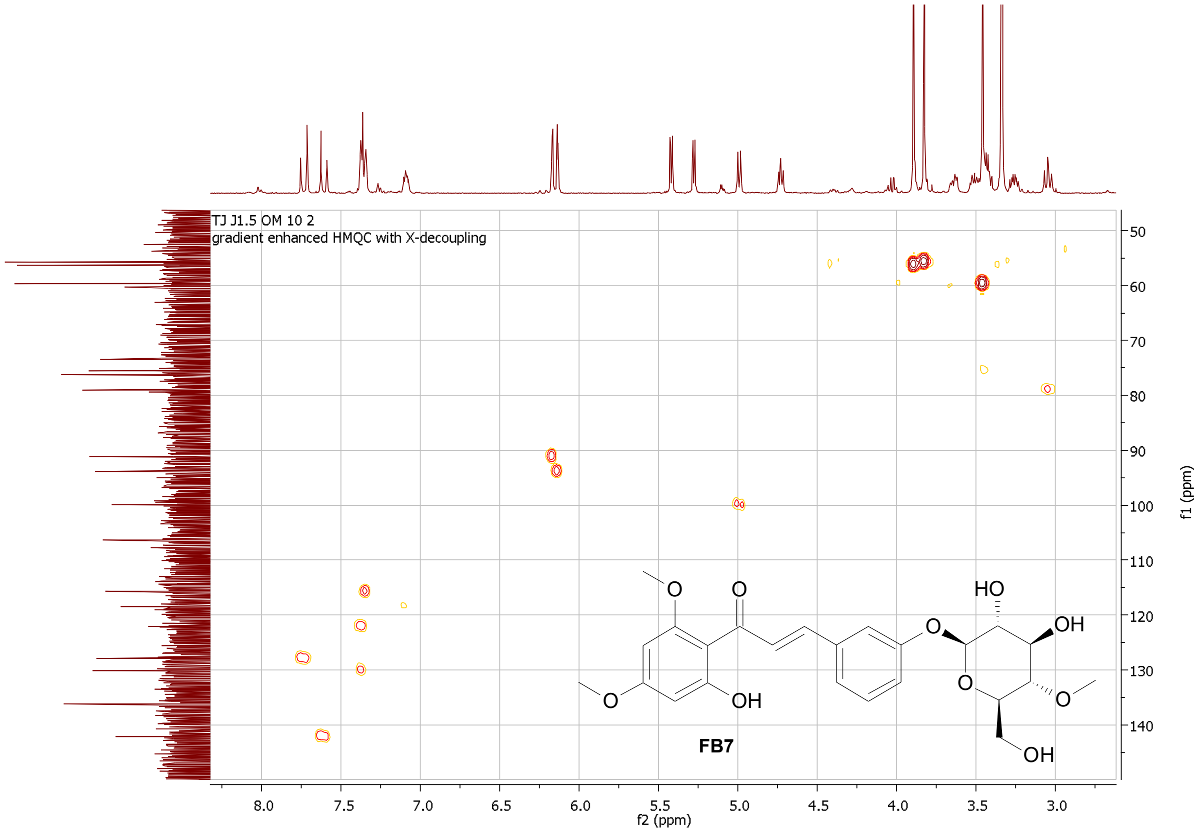

Figure S53. HMBC spectrum of 1-(2’-hydroxy-4’,6’-dimethoxyphenyl)-3-(3’’-*O*-β-D-(4’’’-*O*-methylglucopiranosyl)-phenyl)-prop-2-en-1-on - 3’-*O*-β-D-(4″-*O*-methylglucopyranosyl)-flavokawain B (**FB7**) (DMSO-d_6, 600/151 MHz)

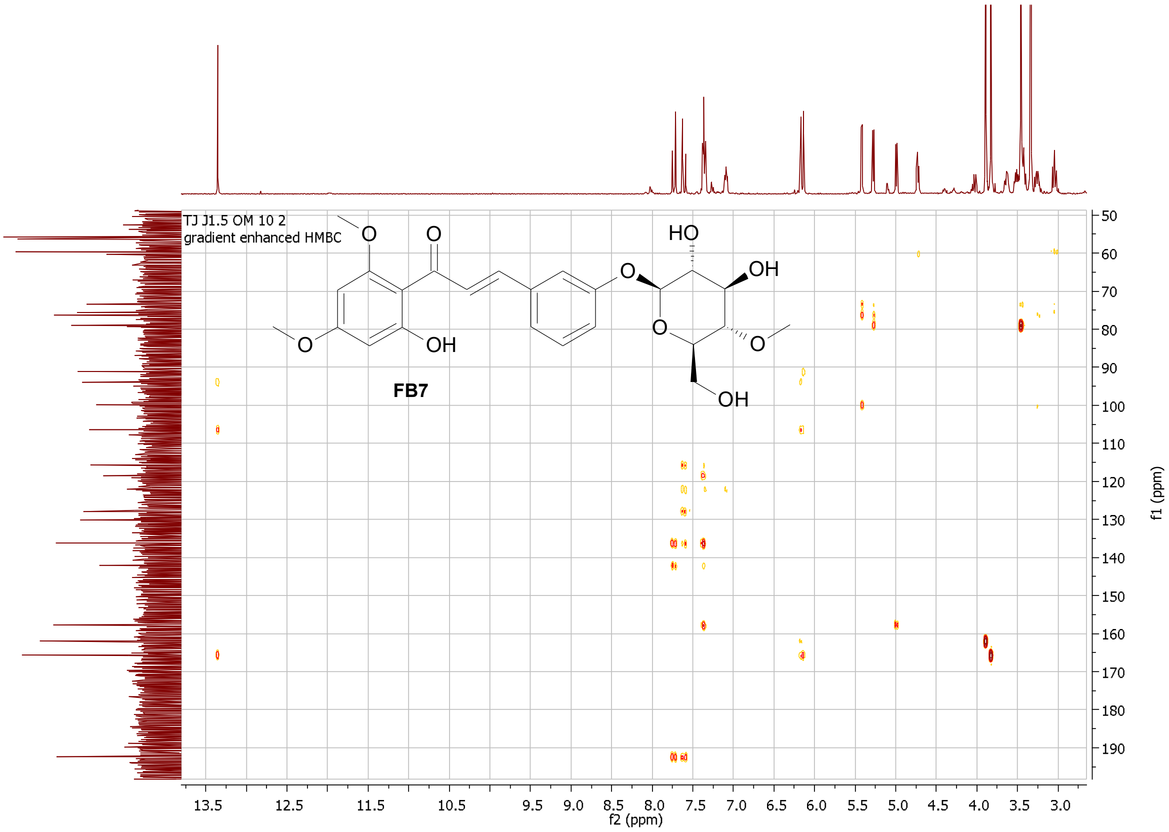

Figure S54. Predicted Boiled-Egg plot from swissADME online web tool for 1-(2’-hydroxy-4’,6’-dimethoxyphenyl)-3-(3’’-*O*-β-D-(4’’’-*O*-methylglucopiranosyl)-phenyl)-prop-2-en-1-on - 3’-*O*-β-D-(4″-*O*-methylglucopyranosyl)-flavokawain B (**FB7**)

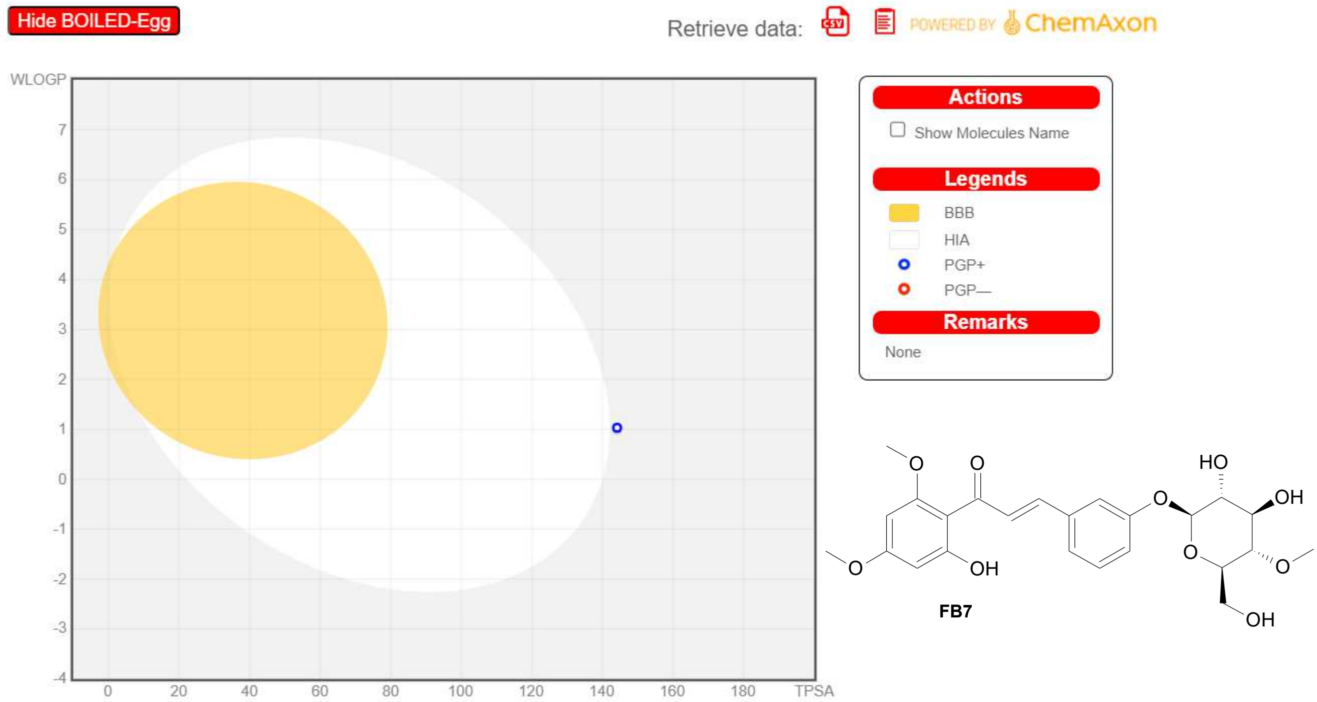

Figure S55. 1-(2’-hydroxy-4’,6’-dimethoxyphenyl)-3-(3’’-*O*-β-D-(4’’’-*O*-methylglucopiranosyl)-phenyl)-prop-2-en-1-on - 3’-*O*-β-D-(4″-*O*-methylglucopyranosyl)-flavokawain B (**FB7**) physicochemical and ADME parameters prediction using the SwissADME modelling

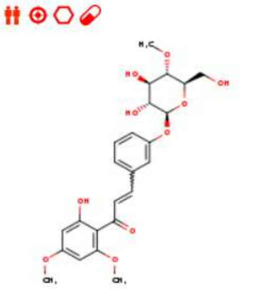

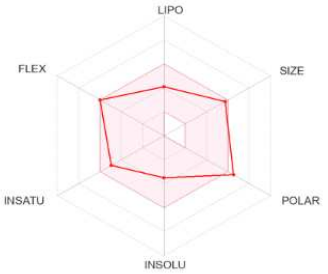

SMILES CO[C@@H]1[C@@H](CO)O[C@H]([C@@H]([C@H]1O)O)Oc1cccc(c1)/C=C/C(=O)c1c(O)cc(cc1OC)OC

Physicochemical Properties	
Formula	C24H28O10
Molecular weight	476.47 g/mol
Num. heavy atoms	34
Num. arom. heavy atoms	12
Fraction Csp3	0.38
Num. rotatable bonds	9
Num. H-bond acceptors	10
Num. H-bond donors	4
Molar Refractivity	120.13
TPSA	144.14 Å²
**Lipophilicity**	
Log $P_{o/w}$ (iLOGP)	3.03
Log $P_{o/w}$ (XLOGP3)	1.65
Log $P_{o/w}$ (WLOGP)	1.03
Log $P_{o/w}$ (MLOGP)	-0.85
Log $P_{o/w}$ (SILICOS-IT)	1.60
Consensus Log $P_{o/w}$	1.29

Water Solubility	
Log *S* (ESOL)	-3.50
Solubility	1.50e-01 mg/ml ; 3.16e-04 mol/l
Class	Soluble
Log *S* (Ali)	-4.29
Solubility	2.44e-02 mg/ml ; 5.12e-05 mol/l
Class	Moderately soluble
Log *S* (SILICOS-IT)	-3.01
Solubility	4.70e-01 mg/ml ; 9.86e-04 mol/l
Class	Soluble
**Pharmacokinetics**	
GI absorption	Low
BBB permeant	No
P-gp substrate	Yes
CYP1A2 inhibitor	No
CYP2C19 inhibitor	No
CYP2C9 inhibitor	No
CYP2D6 inhibitor	No
CYP3A4 inhibitor	No
Log K_p (skin permeation)	-8.03 cm/s
**Druglikeness**	
Lipinski	Yes; 0 violation
Ghose	Yes
Veber	No; 1 violation: TPSA>140
Egan	No; 1 violation: TPSA>131.6
Muegge	Yes
Bioavailability Score	0.55
**Medicinal Chemistry**	
PAINS	0 alert
Brenk	1 alert: michael_acceptor_1
Leadlikeness	No; 2 violations: MW>350, Rotors>7
Synthetic accessibility	5.33

Figure S56. The UV absorption maxima of 1-(2’-hydroxy-4’,6’-dimethoxyphenyl)-3-(3’’-*O*-β-D-(4’’’-*O*-methylglucopiranosyl)-4’’-hydroxyphenyl)-prop-2-en-1-on - 3’-*O*-β-D-(4″-*O*-methylglucopyranosyl)-4’’-hydroxyflavokawain B (**FB8**)

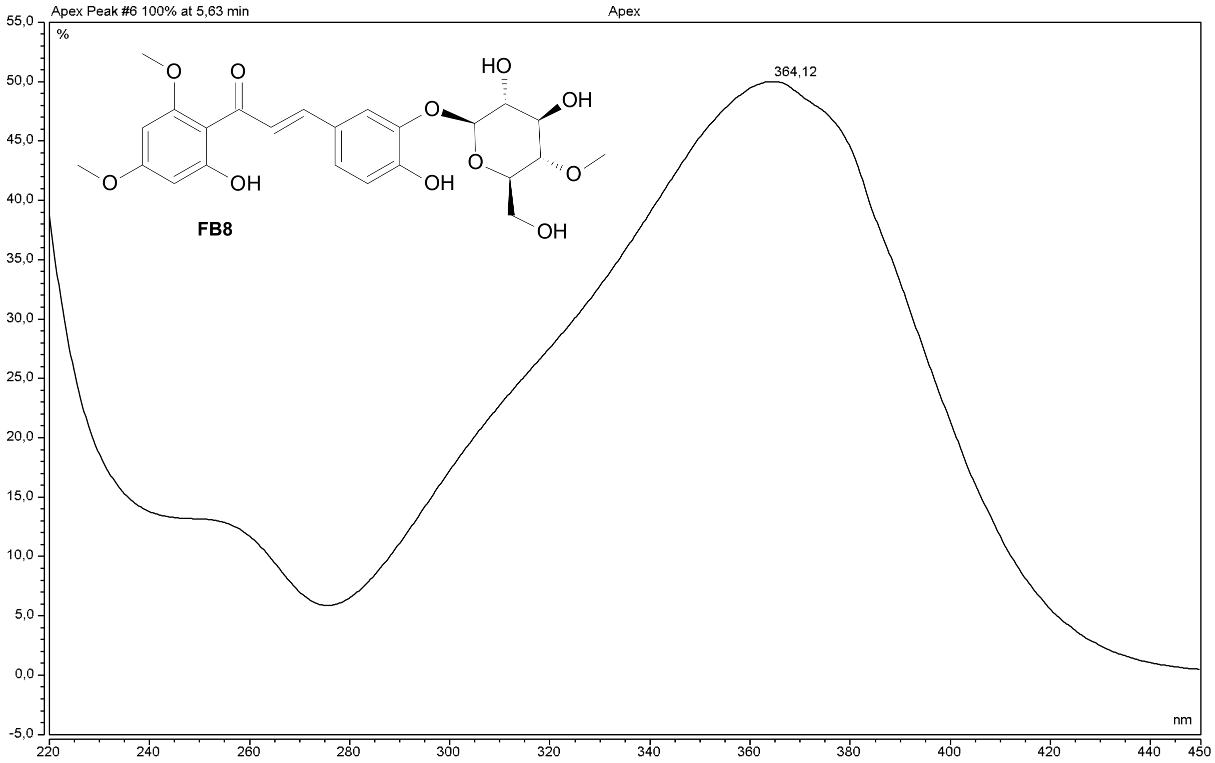

Figure S57. ^{1}H NMR spectra of 1-(2’-hydroxy-4’,6’-dimethoxyphenyl)-3-(3”-*O*-β-D-(4’”-*O*-methylglucopiranosyl)-4”-hydroxyphenyl)-prop-2-en-1-on - 3’-*O*-β-D-(4″-*O*-methylglucopyranosyl)-4’’-hydroxyflavokawain B (**FB8**) (DMSO-d_6, 600 MHz)

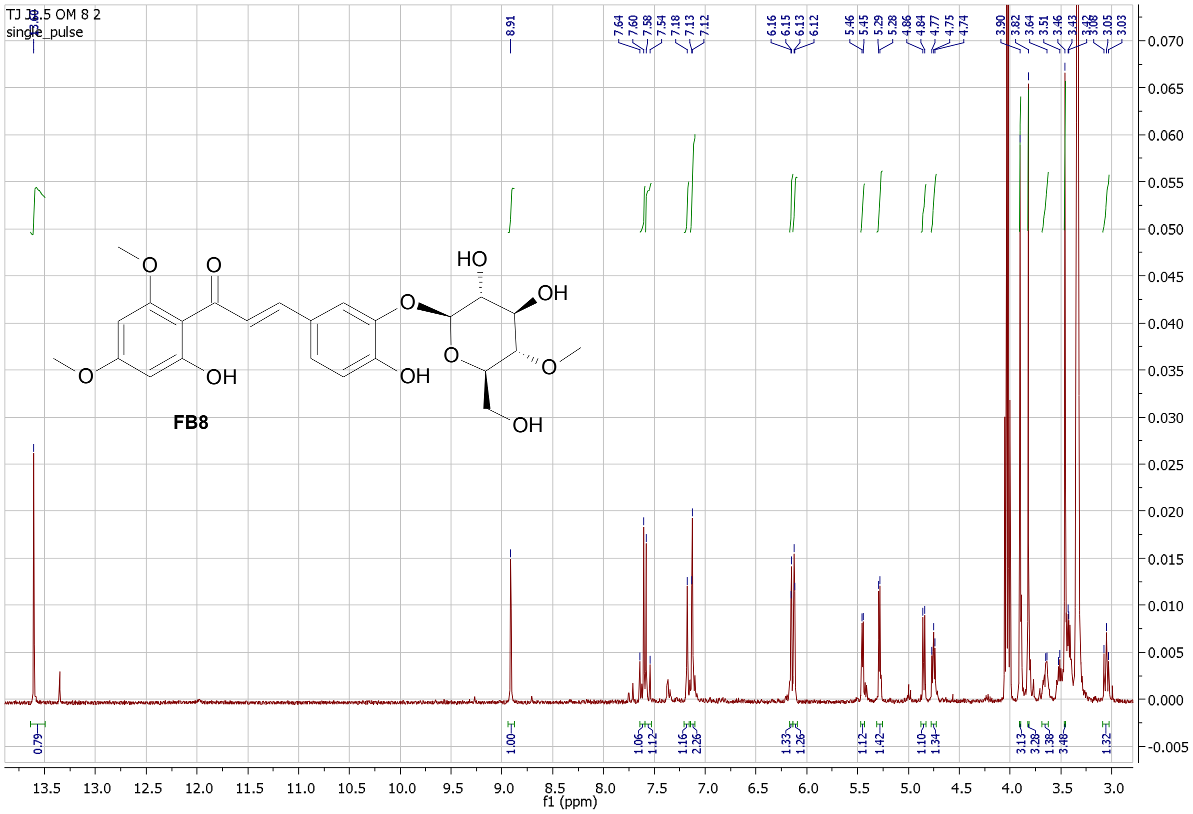

Figure S58. Flavone part of the ^{1}H NMR spectral 1-(2’-hydroxy-4’,6’-dimethoxyphenyl)-3-(3”-*O*-β-D-(4’”-*O*-methylglucopiranosyl)-4”-hydroxyphenyl)-prop-2-en-1-on - 3’-*O*-β-D-(4″-*O*-methylglucopyranosyl)-4’’-hydroxyflavokawain B (**FB8**) (DMSO-d_6, 600 MHz)

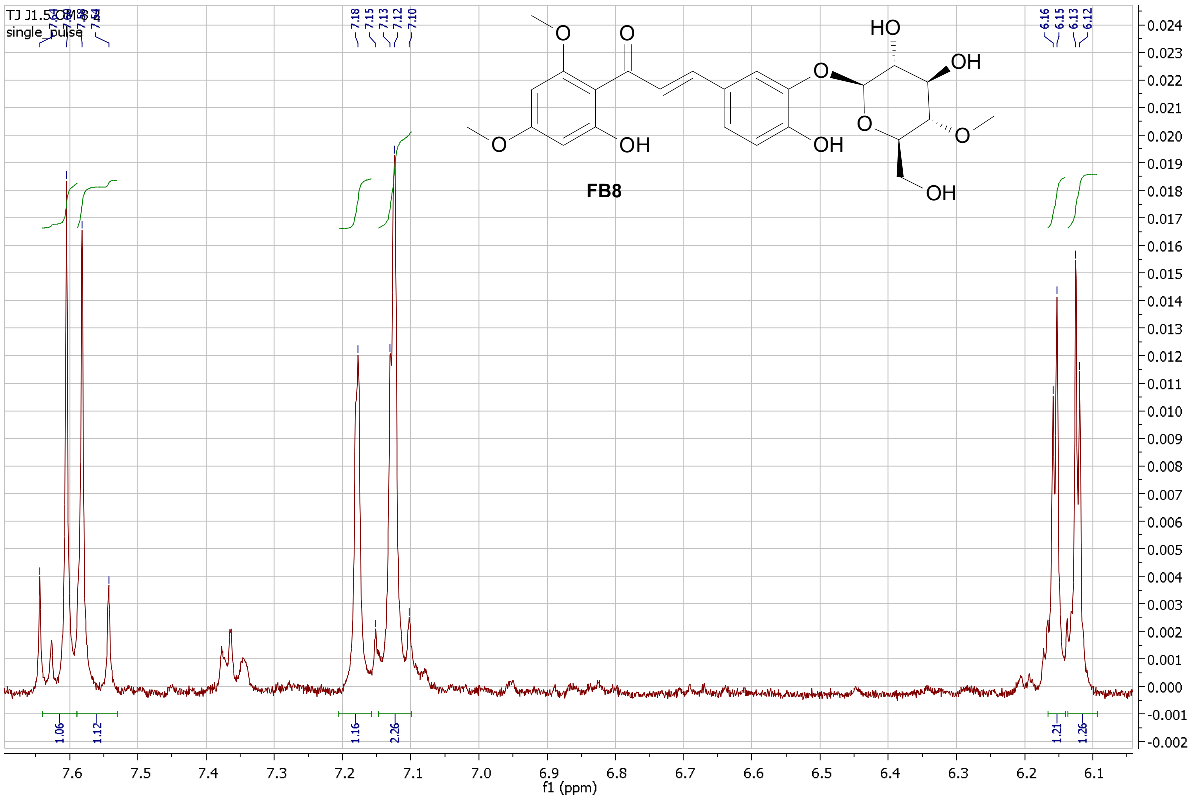

Figure S59. ^{13}C NMR spectra of 1-(2'-hydroxy-4',6'-dimethoxyphenyl)-3-(3''-*O*-β-D-(4'''-*O*-methylglucopiranosyl)-4''-hydroxyphenyl)-prop-2-en-1-on - 3'-*O*-β-D-(4″-*O*-methylglucopyranosyl)-4''-hydroxyflavokawain B (**FB8**) (DMSO-d_6, 151 MHz)

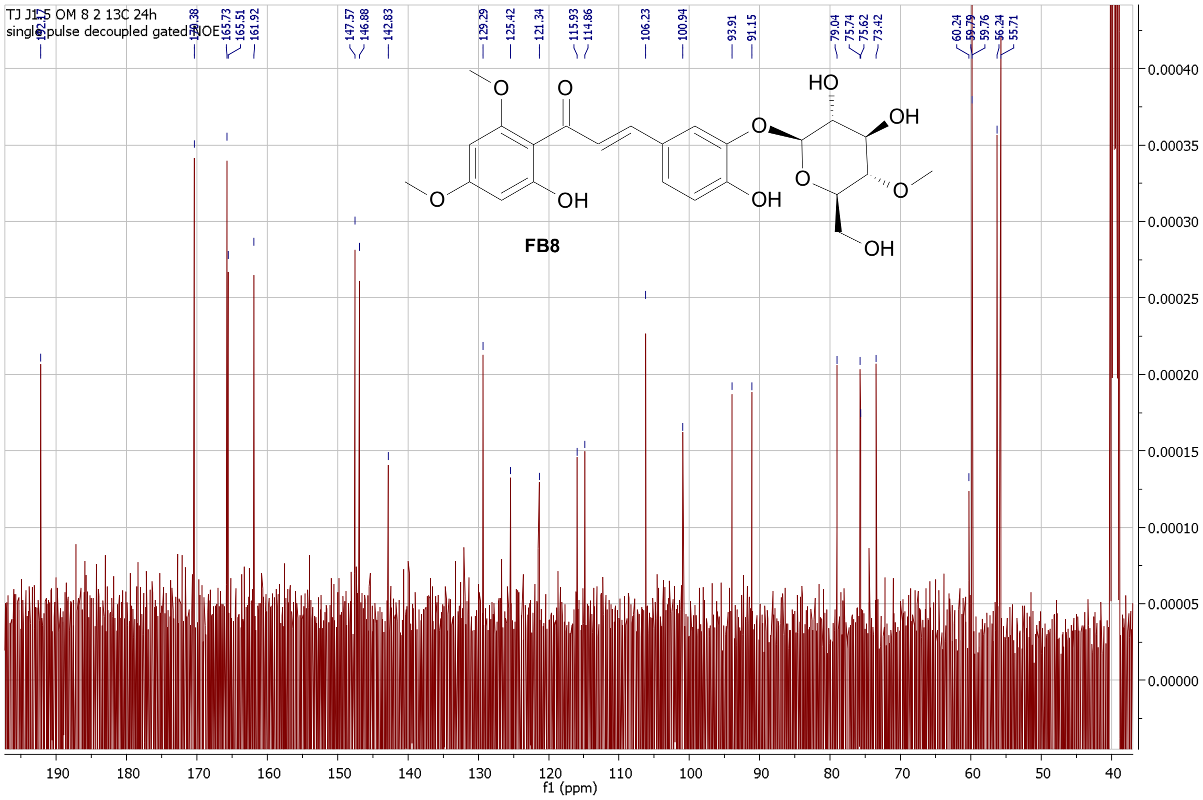

Figure S60. COSY spectrum of 1-(2'-hydroxy-4',6'-dimethoxyphenyl)-3-(3''-*O*-β-D-(4'''-*O*-methylglucopiranosyl)-4''-hydroxyphenyl)-prop-2-en-1-on - 3'-*O*-β-D-(4″-*O*-methylglucopyranosyl)-4''-hydroxyflavokawain B (**FB8**) (DMSO-d_6, 600 MHz)

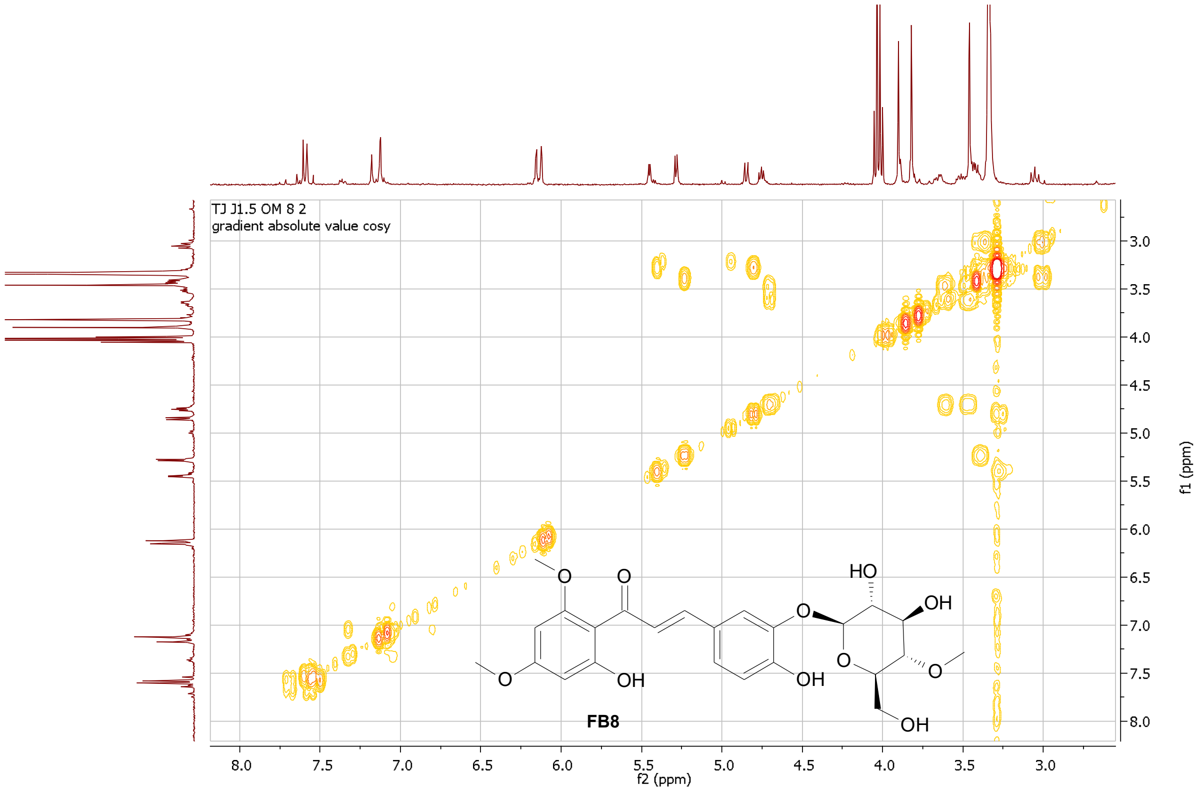

Figure S61. HSQC spectrum of 1-(2'-hydroxy-4',6'-dimethoxyphenyl)-3-(3''-*O*-β-D-(4'''-*O*-methylglucopiranosyl)-4''-hydroxyphenyl)-prop-2-en-1-on - 3'-*O*-β-D-(4″-*O*-methylglucopyranosyl)-4''-hydroxyflavokawain B (**FB8**) (DMSO-d_6, 600/151 MHz)

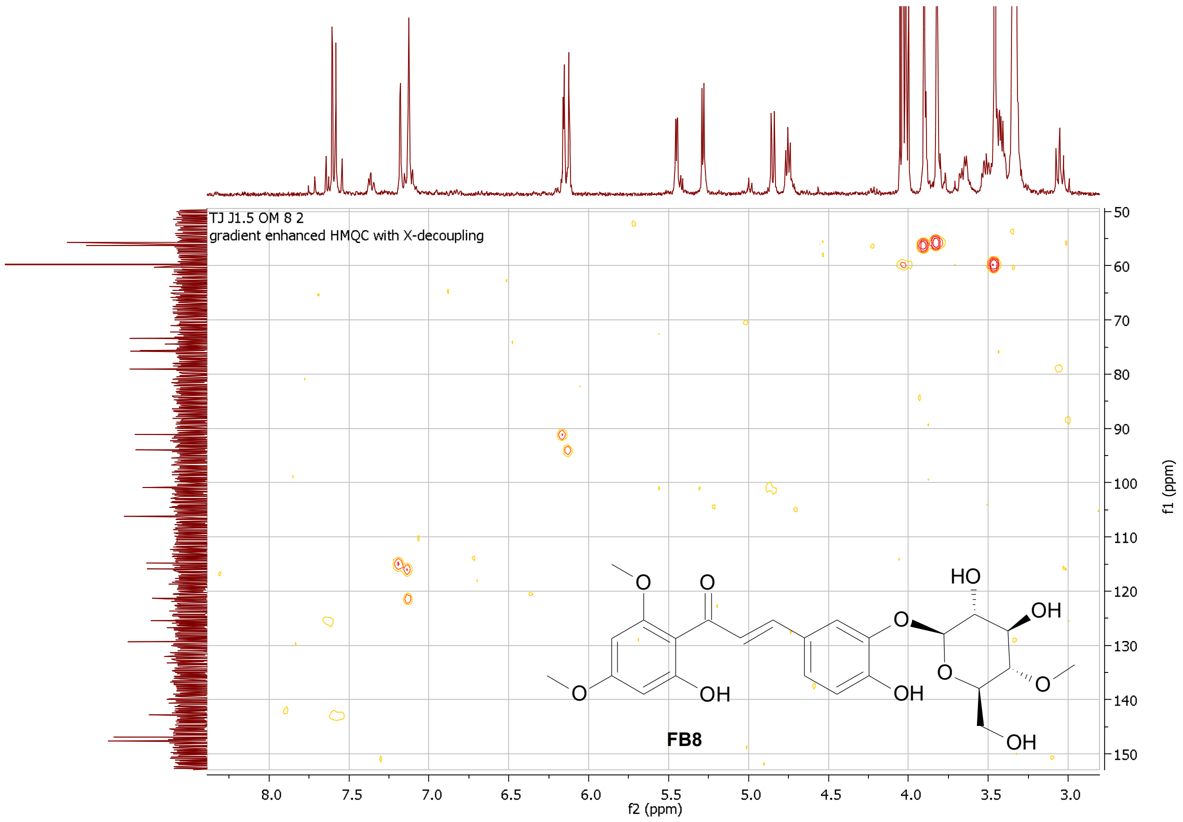

Figure S62. HMBC spectrum of 1-(2'-hydroxy-4',6'-dimethoxyphenyl)-3-(3''-*O*-β-D-(4'''-*O*-methylglucopiranosyl)-4''-hydroxyphenyl)-prop-2-en-1-on - 3'-*O*-β-D-(4″-*O*-methylglucopyranosyl)-4''-hydroxyflavokawain B (**FB8**) (DMSO-d_6, 600/151 MHz)

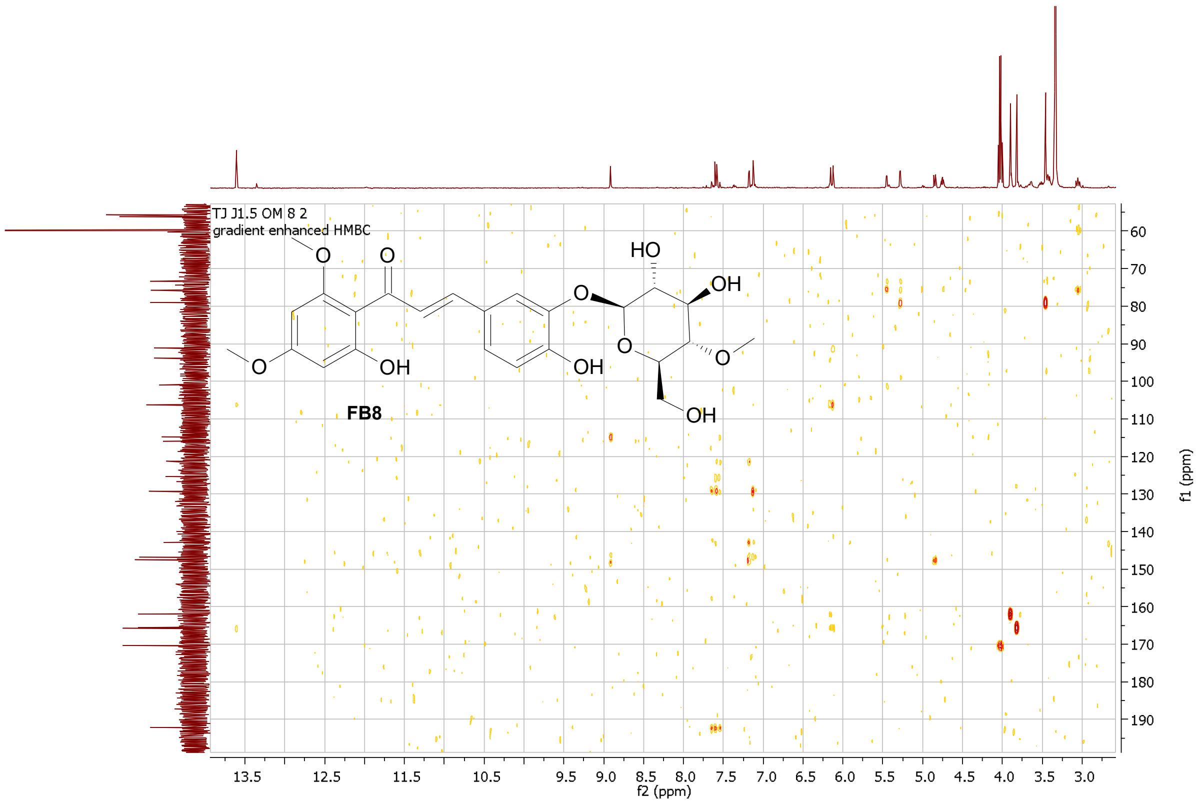

Figure S63. Predicted Boiled-Egg plot from swissADME online web tool for 1-(2’-hydroxy-4’,6’-dimethoxyphenyl)-3-(3’’-*O*-β-D-(4’’’-*O*-methylglucopiranosyl)-4’’-hydroxyphenyl)-prop-2-en-1-on - 3’-*O*-β-D-(4″-*O*-methylglucopyranosyl)-4’’-hydroxyflavokawain B (**FB8**)

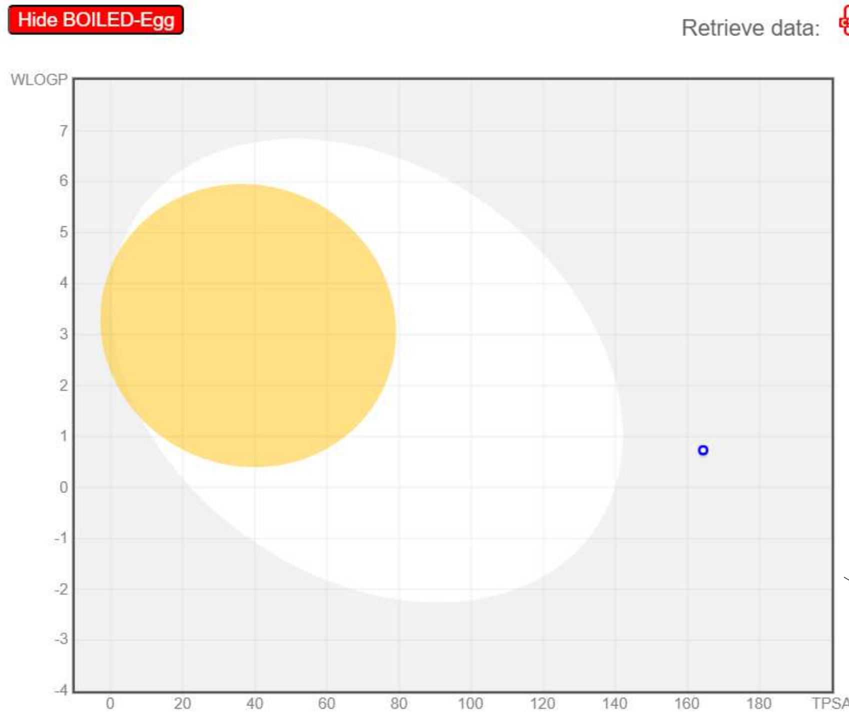

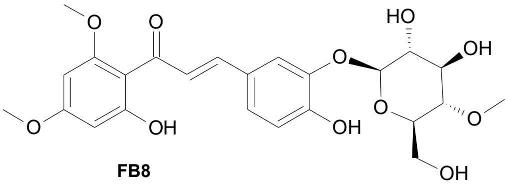

FB8

Figure S64. 1-(2’-hydroxy-4’,6’-dimethoxyphenyl)-3-(3’’-*O*-β-D-(4’’’-*O*-methylglucopiranosyl)-4’’-hydroxyphenyl)-prop-2-en-1-on - 3’-*O*-β-D-(4″-*O*-methylglucopyranosyl)-4’’-hydroxyflavokawain B (**FB8**) physicochemical and ADME parameters prediction using the SwissADME modelling

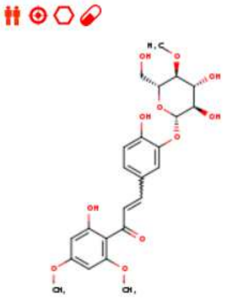

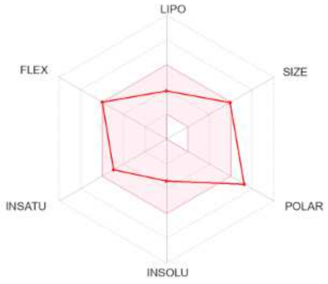

SMILES CO[C@@H]1[C@@H](CO)O[C@H]([C@@H]([C@H]1O)O)Oc1cc(ccc1O)/C=C/C(=O)c1c(O)cc(cc1OC)OC

Physicochemical Properties	
Formula	C24H28O11
Molecular weight	492.47 g/mol
Num. heavy atoms	35
Num. arom. heavy atoms	12
Fraction Csp3	0.38
Num. rotatable bonds	9
Num. H-bond acceptors	11
Num. H-bond donors	5
Molar Refractivity	122.15
TPSA	164.37 Å²

Lipophilicity	
Log $P_{o/w}$ (iLOGP)	2.85
Log $P_{o/w}$ (XLOGP3)	1.30
Log $P_{o/w}$ (WLOGP)	0.73
Log $P_{o/w}$ (MLOGP)	-1.35
Log $P_{o/w}$ (SILICOS-IT)	1.14
Consensus Log $P_{o/w}$	0.94

Water Solubility	
Log *S* (ESOL)	-3.37
Solubility	2.09e-01 mg/ml ; 4.25e-04 mol/l
Class	Soluble
Log *S* (Ali)	-4.35
Solubility	2.19e-02 mg/ml ; 4.45e-05 mol/l
Class	Moderately soluble
Log *S* (SILICOS-IT)	-2.41
Solubility	1.90e+00 mg/ml ; 3.86e-03 mol/l
Class	Soluble

Pharmacokinetics	
GI absorption	Low
BBB permeant	No
P-gp substrate	Yes
CYP1A2 inhibitor	No
CYP2C19 inhibitor	No
CYP2C9 inhibitor	No
CYP2D6 inhibitor	No
CYP3A4 inhibitor	No
Log K_p (skin permeation)	-8.38 cm/s

Druglikeness	
Lipinski	Yes; 1 violation: NorO>10
Ghose	No; 1 violation: MW>480
Veber	No; 1 violation: TPSA>140
Egan	No; 1 violation: TPSA>131.6
Muegge	No; 2 violations: TPSA>150, H-acc>10
Bioavailability Score	0.55

Medicinal Chemistry	
PAINS	0 alert
Brenk	1 alert: michael_acceptor_1
Leadlikeness	No; 2 violations: MW>350, Rotors>7
Synthetic accessibility	5.38
